# Barriers and enablers of community-based health insurance enrollment in the Sidama national regional state, Southern Ethiopia, 2024: A qualitative study

**DOI:** 10.1371/journal.pgph.0004310

**Published:** 2025-09-11

**Authors:** Kare Chawicha Debessa, Keneni Gutema Negeri, Mesay Hailu Dangisso

**Affiliations:** 1 School of Public Health, College of Medicine and Health Sciences, Hawassa University, Hawassa, Ethiopia; 2 Ethiopia Public Health Institute, Addis Ababa, Ethiopia; Makerere University School of Public Health, UGANDA

## Abstract

The universal health coverage (UHC) efforts in low- and middle-income countries such as Ethiopia encounter significant challenges in the implementation of community-based health insurance (CBHI) programs. This study investigated barriers and enablers influencing CBHI enrollment in Sidama National Regional State. Using purposive sampling, 48 participants were recruited, including four focus groups (12 participants each) and 20 key informant interviews. Participants comprised CBHI members (n = 5), non-members (n = 5), community leaders (n = 4), policymakers (n = 3), and healthcare providers (n = 3). Data collection occurred over three weeks in May 2024, with thematic analysis employed following phenomenological principles. Ethical considerations such as informed consent, confidentiality, and data protection were strictly observed. Findings indicate that awareness of CBHI has improved across Sidama; however, persistent misconceptions remain, including beliefs that CBHI is exclusively for the poor or is synonymous with preparing for illness. Several structural barriers were identified, notably ambiguous governance frameworks, a lack of accountability, and inadequate human resources. Issues in service delivery further undermine program effectiveness, characterized by discrimination against CBHI members, frequent shortages of essential medications and laboratory services, and delays in payment processes. Financial management challenges, lack of transparency, and the absence of grievance mechanisms were also found to erode community trust in the CBHI system. Despite these challenges, CBHI is recognized as valuable for reducing healthcare costs and enhancing access, particularly among women and low-income households, empowering women’s autonomy in health decisions. Community-based health insurance (CBHI) in the Sidama Region has improved awareness and access to healthcare. However, sustainability is challenged by misconceptions, governance challenges, and service delivery issues, which undermine trust and reduce enrollment and retention. Addressing governance, transparency, equity, and community involvement is crucial to strengthening CBHI and ensuring equitable healthcare access.

## Introduction

The global pursuit of universal health coverage (UHC) has intensified as nations strive to ensure equitable healthcare access for all citizens [1]. In this effort, community-based health insurance (CBHI) has emerged as a vital strategy for addressing health financing challenges, especially within low- and middle-income countries (LMICs). By protecting vulnerable populations from the financial risks associated with healthcare utilization and promoting preventive care, CBHI helps mitigate the burden of out-of-pocket expenditures [2]. In Africa, where health systems are strained by high disease prevalence, funding limitations, and infrastructural inadequacies, CBHI implementation represents a critical step toward achieving health equity [3]. Sub-Saharan Africa particularly contends with immense barriers to healthcare access; over 400 million people in the region lack essential services, many of whom face impoverishment due to direct healthcare costs [4].

Within East Africa, socioeconomic disparities have prompted increasing adoption of CBHI as a mechanism for enhancing equitable healthcare access [5]. Ethiopia, characterized by a large and diverse population, offers a salient context for examining CBHI dynamics given its ongoing health challenges. The Sidama region, in particular, provides a critical case study due to its unique socio-economic and cultural attributes that influence CBHI enrollment. Despite the recognized value of CBHI as a tool against catastrophic health expenditures, enrollment rates in Sidama remain low, hindered by an array of factors, including distrust in scheme management and poor dissemination of information [6]. Socioeconomic barriers such as poverty and unemployment, combined with informational deficits and misconceptions, perpetuate low uptake, underscoring the need for a detailed understanding of local contexts to inform effective enrollment strategies [6].

The Theory of Planned Behavior (TPB) provides a valuable theoretical lens to explore the determinants of CBHI enrollment in Sidama. According to Ajzen (2020), behavioral intentions are shaped by attitudes toward the behavior, subjective norms, and perceived behavioral control [7]. Positive attitudes toward CBHI - recognizing its benefits in financial protection and healthcare access - can motivate enrollment, while negative perceptions deter participation [8]. Subjective norms, involving influences from family, peers, and community leaders, further impact enrollment decisions; endorsement by respected figures can encourage uptake, whereas skepticism reduces it [9]. Perceived behavioral control reflects individuals’ confidence in their ability to enroll, influenced by economic conditions and access to essential information, highlighting the importance of minimizing financial and informational barriers to promote enrollment [10].

Empirical evidence underscores the multifaceted nature of CBHI enrollment barriers, encompassing economic constraints such as premium affordability and indirect costs related to healthcare usage [11,12], as well as cultural factors like healthcare beliefs and skepticism about insurance schemes. Qualitative findings reveal a pervasive lack of awareness about CBHI benefits, which sustains low participation rates [13]. Conversely, community engagement and targeted communication strategies have shown promise in enhancing enrollment [14]. Despite these insights, there remains a deficiency of qualitative research focusing on lived experiences and localized socio-cultural determinants of CBHI enrollment, particularly within southern Ethiopia and the Sidama region. This gap limits contextualized understanding, vital for designing culturally sensitive and effective interventions [15–17].

This study sought to address these gaps by qualitatively examining the barriers and enablers influencing CBHI enrollment and retention in the Sidama region of Ethiopia. The elucidation of socio-cultural and economic factors shaping enrollment decisions aims to inform public health policy and community-based initiatives that enhance sustainable CBHI uptake and retention. Such efforts align with the broader United Nations Sustainable Development Goals by reducing catastrophic health expenditures and improving health outcomes within vulnerable populations, such as women. Insights from this investigation are intended to inform community-centered interventions applicable to similar socio-economic contexts in Ethiopia and beyond.

## Methods

### Ethics statement

The ethical framework guiding this qualitative study was rigorously aligned with the ethical standards delineated in the Declaration of Helsinki. The study protocol underwent a thorough review and received approval from the Institutional Review Board (IRB) of the College of Medicine and Health Sciences at Hawassa University, which was documented under the reference number IRB/02/16. Additionally, the study team secured a letter of support from the School of Public Health and the Sidama Regional Health Bureau, which was disseminated to local district health offices, healthcare facility leaders, and kebele administrators before initiating data collection.

Before participation, all study participants were provided with detailed disclosures concerning the purpose and scope of the study, the techniques employed for data collection, privacy considerations, voluntary participation, potential benefits of engagement, and any associated risks. After this comprehensive informational briefing, written informed consent was solicited from each study participant before commencing any data collection activities. Throughout the study process, the utmost care was taken to maintain participant confidentiality. Interviews were conducted in private settings tailored to participants’ preferences, thereby respecting individual privacy and comfort levels. Specific personal identifiers were deliberately excluded from documentation throughout the data collection, storage, and transcription phases, thereby fostering comprehensive participant anonymity. Furthermore, all recorded audio files were anonymized during transcription, with access strictly restricted to designated members of the research team to further safeguard participants’ identities.

### Study setting

The study was conducted in the Sidama Region of Ethiopia. Ethiopia, known for its ethnic diversity, is administratively divided into twelve regions and two city administrations [18]. The Sidama region, one of these national and regional states, was newly established on June 18, 2020. This region is situated in the southern part of the country, approximately 273 kilometers south of the capital city, Addis Ababa. The Sidama region is composed of four zones: northern, southern, central, and eastern, along with one city administration [19].

The Sidama region contains 37 districts and 636 kebeles, which are the smallest administrative units in Ethiopia [20]. Based on data from the Sidama Region Health Bureau, the total population in the region was 4,873,216. The healthcare infrastructure in the region includes 551 health posts, 138 health centers, five general hospitals, and 17 primary hospitals. As of last year, data indicated that the overall coverage of community-based health insurance (CBHI) in the region reached 415,835 individuals, representing 69.6% of the total population [21].

### Study design and period

This qualitative study utilized a phenomenological study design, with data collection taking place from May 8 to May 30, 2024. This manuscript was prepared according to the COREQ checklist (S1 Checklist).

### Study population and eligibility criteria

This qualitative study was designed to encompass a diverse array of stakeholders across various administrative levels and community settings within the Sidama region of Ethiopia. The study population comprised adult individuals aged 18 years and older, including those enrolled in the community-based health insurance (CBHI) scheme as well as individuals who were not members.

Participants were identified based on their roles, which included household heads associated with either CBHI membership or non-membership, regular premium contributors, individuals who had previously dropped out from the scheme, and administrators at the kebele and woreda levels, in addition to leaders affiliated with health facilities, the Ethiopia health insurance services, Ethiopia pharmaceuticals supply services, and the regional health bureau.

The eligibility criteria were meticulously delineated to include both male and female adults who met the aforementioned roles and responsibilities within the community and at different administrative levels. Moreover, the sampling specifically targeted *kebeles* with the highest and lowest rates of CBHI enrollment, which was instrumental in identifying critical barriers and enablers impacting its enrollment decisions. To uphold the credibility and validity of participant responses, particularly among community leaders and healthcare providers, a residency requirement of at least twelve months before data collection was established. Consequently, this criterion was pivotal in ensuring that participants possessed a sufficient depth of knowledge and experiential understanding of the CBHI scheme.

Individuals engaged in CBHI-related activities for less than twelve months were intentionally excluded from key informant interviews (KIIs) to minimize potential biases associated with limited exposure. Furthermore, to prevent information bias, participants involved in the quantitative component of the study were excluded from participation in both KIIs and focus group discussions (FGDs). Additionally, individuals with severe physical or mental health conditions were excluded from the study, recognizing that such circumstances could adversely affect their capacity to engage meaningfully and navigate the informed consent process.

### Participants recruitment

Study participants’ recruitment for this study was guided by a comprehensive, multi-layered strategy that prioritized the inclusion of a diverse and representative sample. The process began with close collaboration between the study team and key local stakeholders, such as health extension workers, woreda and city health offices, *kebele* administrators, and community leaders. These partnerships were instrumental in both identifying and reaching out to individuals who were eligible for participation in Focus Group Discussions (FGDs) and Key Informant Interviews (KIIs). Health extension workers played a central role in sharing information about the study within their respective communities, while community leaders assisted in pinpointing individuals who met the study’s eligibility requirements.

To enhance the credibility and rigor of the sampling process, the study established explicit inclusion and exclusion criteria for both FGDs and KIIs, moving beyond purposive sampling alone. For FGDs, eligible participants were adults aged 18 years or older who had resided in the study area for at least one year. They were current or recent beneficiaries of community-based health insurance (CBHI). In addition, non-CBHI members were also included, such as the dropouts of the schemes. The selection process also aimed to ensure diversity across gender, age groups, and socio-economic backgrounds. Individuals with cognitive or communication impairments were excluded to maintain the integrity of community perspectives.

For KIIs, the recruitment focused on individuals occupying leadership or decision-making roles relevant to CBHI implementation. This included *kebele* leaders, woreda and city health officials, as well as representatives from institutions such as the Ethiopian Pharmaceutical Supply Services, Ethiopian Health Insurance Services, and the Regional Health Bureau. Senior staff from health centers, general hospitals, and referral hospitals serving CBHI beneficiaries were also included, provided they had at least one year of experience in their current role and demonstrated substantial knowledge of CBHI operations. Those with less than one year of experience or with direct involvement in the study’s design or implementation were excluded.

The recruitment process combined direct outreach, referrals from local stakeholders, and broader community engagement activities. Information about the study’s objectives, methodology, and significance was communicated both verbally and in writing, ensuring that all potential participants were well-informed and able to make considered decisions regarding their involvement. This approach, underpinned by clear eligibility criteria and systematic procedures, was designed to maximize the diversity, depth, and validity of the data collected, thereby strengthening the overall credibility of the study’s findings.

### Sampling technique and sample size

The study implemented a purposive sampling technique utilizing a maximum variance sampling approach, often recognized in the literature as heterogeneous sampling. This methodological choice was designed to deliberately select participants capable of providing detailed and comprehensive insights [22] into the barriers and enablers associated with CBHI enrollment. The investigators employed this sampling technique to obtain a diverse array of perspectives reflective of various demographic characteristics, CBHI membership statuses, socioeconomic backgrounds, and community roles consequential to the CBHI framework.

Ultimately, the study encompassed a total of four FGDs, each consisting of 12 participants drawn from four distinct *kebeles*, two sessions per *kebele*, resulting in a total of 48 FGD participants. In addition to this, a series of 20 key informant interviews was conducted, drawn from the region to *kebele* level, categorizing participants as follows: CBHI members (n = 5), non-members (n = 5), community leaders (n = 4), policymakers (n = 3), and healthcare providers (n = 3). This structured approach to sampling aimed to create a rich narrative surrounding the CBHI enrollment landscape and the surrounding systemic dynamics.

### Data collection procedures and instruments

The qualitative data collection process for this study employed two primary techniques: focus group discussions (FGDs) and key informant interviews (KIIs). The FGDs were intentionally designed to facilitate collaborative dialogue among community members [23], fostering an environment conducive to sharing experiences, perspectives, and insights concerning the multifaceted barriers and enabling factors linked with CBHI enrollment. Each FGD typically included 12 participants and had a duration of approximately 90 minutes, thereby allowing ample time for in-depth discussions and the exploration of diverse viewpoints.

Conversely, the KIIs were structured to engage prominent leaders and officials operating at *kebele*, woreda, and regional administrative levels. This individual interview format provided a private and confidential setting that enabled the exploration of personal experiences, reflections, and detailed anecdotes [24] related to the barriers and enablers of CBHI enrollment. For data collection, an open-ended interview guide was adapted from extant studies [25,26], which served as the foundational instrument utilized throughout the study (refer to S3 Text). All the FGDs & KIIs sessions were audio recorded with the consent of study participants.

This guide was specifically designed to enhance the validity of the study by incorporating items derived from previously validated tools relevant to comparable populations and constructs. The initial version of the guide was drafted in English and subsequently translated into the local dialect, *Sidaamu Afoo*. This dual translation process emphasized cultural relevance and aimed to ensure study participants’ comprehension of the content presented.

To maintain the integrity and fidelity of meaning throughout the translation process, rigorous quality assurance measures were adopted [27]. Two bilingual experts facilitated both the translation and back-translation processes, ensuring that the original intent of the questions was preserved while employing language deemed culturally appropriate. Importantly, all data collection was executed in the local dialect, *Sidaamu Afoo*, with data collectors fluent in this language, thereby optimizing effective communication throughout the data collection activities.

Before initiating the actual data collection process, a pre-test of the translated questionnaire was conducted with a sample of 5% of selected individuals from *Hitata kebele*, *Tabor* sub-city, and Hawassa city. Feedback gathered from both participants and data collectors proved instrumental in refining the guide, thus enhancing clarity, addressing potential coding errors, and confirming cultural sensitivity. This iterative pre-testing process ensured the final instrumentation was well-adapted and relevant to the study’s objectives.

Each FGD and KII session was moderated by three females & four Master of Public Health (MPH) experts, who have an academic rank of lecturer and assistant professor, complemented by trained note-takers who were fluent in *Sidaamu Afoo*. Data collectors with expertise in qualitative research were engaged by the local community, thus enhancing community representation and insight throughout the data collection process. Note-taking served to provide documentation of discussions, complementing audio recordings. Probing questions were employed to elicit deeper insights into complex themes under discussion. Overall, conducting the FGDs and key informant interviews in the local dialect not only enhanced participant engagement but also enriched the qualitative data collected, allowing for more robust analyses. Data collection continued until thematic saturation was reached, which was determined when no new codes or themes emerged from consecutive interviews. This approach ensured that the data comprehensively captured the range of study participant perspectives and narratives, consistent with established qualitative research approaches [28].

### The rigor of the study

To ensure the credibility and rigor of the qualitative data collection and analytical processes, the study team established a comprehensive framework of measures designed to enhance the quality, reliability, and trustworthiness of the findings. These efforts were anchored in established best practices within qualitative study [29]. A pivotal facet of this endeavor involved fostering proactive communication among the study team members through regular meetings. These collaborative sessions provided a vital platform to evaluate important themes, discuss potential enhancements to the data collection guides and topics, and identify strategies for refining the probing techniques employed by interviewers. The team yielded richer and more insightful qualitative data through consistent reflection on the data collection process and implementation of necessary adaptations.

Additionally, the study team placed a strong emphasis on developing probing skills among investigators, enabling deeper exploration of participant responses and clarification of any ambiguities. This approach served to reduce the risk of misinterpretation and contributed to a more comprehensive understanding of the participants’ perspectives. Furthermore, the practice of respondent validation constituted another critical step implemented throughout the data collection [30]. Data collectors actively engaged in clarifying participants’ statements and ensuring accurate comprehension and interpretation of their words, thereby minimizing the likelihood of misrepresentation and ensuring that the viewpoints articulated by participants were faithfully captured.

The rigor of qualitative analysis was further bolstered through meticulous review and comprehensive readings of transcripts by the principal investigator. This process enhanced the accuracy and reliability of the data, laying a solid foundation for subsequent coding and thematic analysis [29]. To maintain quality control during the transcription phase, a four-tier review process was established. This involved self-review conducted by transcribers, peer review, and cross-checking of the transcripts against original audio recordings undertaken by a dedicated quality assurance team.

The data analysis was conducted using ATLAS.ti software, which enabled systematic organization and coding of qualitative data. The study team implemented a collaborative coding technique to enhance coding consistency. To maintain coding consistency among the study team, the team members independently coded a subset of transcripts, followed by joint review sessions to compare coding decisions and resolve any discrepancies through discussion. This iterative process ensured inter-coder reliability and reinforced the methodological rigor of the analysis [31]. Throughout the coding and analytical processes, the principal investigator continuously monitored activities to ensure fidelity was maintained to the meanings articulated by participants.

The study team held meetings together to talk about developing themes, making sure that different viewpoints were included in the analysis. Further, the study team devoted attention to meticulous documentation and established feedback mechanisms to uphold transparency while enabling continuous refinements based on findings presented at various stages of the study. This iterative approach facilitated the prompt identification and resolution of any emerging issues, further enhancing the overall quality of the research endeavor.

### Data analysis

The analysis of qualitative data adhered to a rigorous thematic analysis framework, guided by established best practices within qualitative research. The analysis protocol commenced with the verbatim transcription of all audio recordings from the FGDs and KIIs (see S3 Text). Following transcription, these records in the local language (*Sidaamu Afoo*) were subsequently translated into English to enhance accessibility for international audiences. This dual transcription and translation process was integral in preserving the original linguistic integrity while facilitating the study team’s engagement with the data [32].

After transcription, the analysis proceeded through systematic coding of the transcripts employing both deductive and inductive coding strategies. The deductive component involved the application of predetermined codes aligned with conceptual categories derived from the initial study framework and objectives [33]. On the other hand, inductive coding allowed for the spontaneous emergence of new codes directly from the transcribed data, thereby ensuring that the analysts remained receptive to unanticipated themes and insights [33]. The use of ATLAS.ti qualitative data analysis software proved to be instrumental in facilitating the precision surrounding the coding and analytical processes. This software equipped the study team with the tools necessary for efficient organization, management, and reassembly of the data, thereby allowing for a thorough examination of codes and the identification of patterns across various data sources.

The deeper analytical activities further involved scrutinizing the frequency of specific codes within and across data sources, illuminating salient themes, and discerning relationships among codes, which revealed connections or contradictions within the emerging themes [31]. Throughout this analytical journey, the study team actively engaged in continuous reflection upon and discussion concerning the coded data excerpts, meticulously examining, contrasting, and interpreting these excerpts to derive synthesized meanings and construct richly descriptive accounts reflecting the complexities of the CBHI enrollment landscape.

### Inclusivity in global research

Global research inclusivity mandates rigorous ethical, cultural, and scientific considerations to ensure equitable representation and respect for participating communities. Ethical imperatives involve securing appropriate approvals from relevant stakeholders, maintaining integrity in authorship and research practices, and avoiding exploitative actions such as extractive research and ethical dumping. Employing culturally sensitive methodologies and community-centered frameworks is essential to uphold scientific validity, build trust, and advance social justice by inclusively engaging historically marginalized and underrepresented groups throughout the study process. Additional information is included in the Supporting Information (S4 Checklist).

## Results

### Socio-demographic characteristics of the study participants

The socio-demographic characteristics of study participants involved in community-based health insurance (CBHI) implementation in the Sidama region of Ethiopia are presented in the table below. Among the key informants, 11 out of 20 (55%) were over 35 years old, indicating a focus on experienced professionals. Conversely, the focus group discussants showed a wider age distribution: 20 out of 48 (41.7%) were over 45 years, while 16 out of 48 (33.3%) were under 35 years (Table 1).

**Table 1:** The socio-demographic characteristics of the key informants’ interviews and focus group discussants in Sidama National Regional State, Ethiopia, 2024.

Characteristics	Categories	Key informants interview (N = 20)		Focus groups discussion (N = 48)	
		Frequency	Percentage	Frequency	Percentage
Age	<35 years	9	45	16	33.3
	35-45 years			12	25
	>45 years	11	55	20	41.7
Educational level	No formal education			7	14.5
	Primary			28	58.3
	Secondary			13	27.1
	Diploma	8	40		
		Masters	12	60	
	Experience	5-10 years	4	20	
		>10 years	16	80	
	Current position	Kebele Leader	4	20	
		Hospital CEO	3	15	
		Woreda/city administration/WrHo heads	3	15	
		CBHI agency head/CBHI focal	3	15	
		Others	7	35	

*Others include community representatives, RHB, EPSA, Abt Associates, and PHCU.

In terms of educational background, 12 out of 20 key informants (60%) held master’s degrees, which reflects a substantial level of expertise relevant to CBHI. On the other hand, the focus group discussants possessed a variety of educational credentials: 7 out of 48 (14.6%) had not attended formal education, and 28 out of 48 (58.3%) had completed only primary education.

Regarding professional experience, 16 out of 20 key informants (80%) reported having over 10 years of experience in their roles, showcasing their understanding of community-based health insurance and the dynamics of the health system. While the focus group discussants did not provide data on their professional experience, their input was crucial for capturing community perspectives.

The key informants held diverse positions within the health system, including 4 *Kebele* leaders (20%) and 3 hospital CEOs (15%), thereby enriching insights into the implementation of CBH. The focus group discussants, lacking defined roles in the health system, primarily contributed as community members. Overall, these findings underscore the involvement of both seasoned professionals and community members in the exploration of CBHI implementation, highlighting various perspectives on its challenges and status.

#### Summary of main themes.

The study of community-based health insurance (CBHI) enrollment in Ethiopia’s Sidama region identified key barriers and facilitators. Despite increasing awareness, persistent misconceptions and stigma limited uptake. Structural weaknesses, including ambiguous governance, resource constraints, and weak accountability, undermined sustainability. Poor service delivery, perceived inequities, payment delays, and coercive enrollment practices reduced trust and retention. Financial management challenges and low service quality further discouraged enrollment. Nevertheless, CBHI improved healthcare access, financial protection, and empowerment of vulnerable groups, particularly women and low-income households (Table 2).

**Table 2:** The overview of themes, subthemes, categories, type, and illustrative quotes of barriers & enablers of CBHI enrollment in Sidama region, Ethiopia, 2024.

Theme	Subtheme	Category	Type	Sample illustrative quotes
1. Community knowledge & perception of CBHI	1.1. Awareness of CBHI	Improved awareness	Enabler	“*We know that it is useful, & we know who enrolls & who can’t.”* (FGD, women from the community)
	1.2. Misconceptions	1.1. Misunderstanding & insurance nature	Barrier	*“Thinking that I am going to be sick is planning for myself to get sick.” (*FGD, community member)
	1.3. Necessity of the program	Indispensability	Enabler	*“CBHI has been very useful for us & will continue to be useful in the future.”* (FGD, CBHI member)
2. CBHI structure & governance	2.1. Governance structure	Confusion over the governance structure	Barrier	*“The CBHI is the political agenda. It is led by regional & zonal party leaders.”* (KII, PHCU informant)
	2.2. Human resources	Lack of personnel at all levels	Barrier	*“The current CBHI structure lacks sufficient structure.”* (KII, an informant from RHB)
	2.3. Accountability	Lack of clarity in accountability	Barrier	*“This complex arrangement contributed to false payment claims and accountability problems within the health system.” (KII, Abt Associates)*
3. Enrollment & Retention	3.1. Barriers to enrollment	Service unavailability	Barrier	*“They do not treat those who say ‘I am a CBHI member ‘& those who can’t pay in cash.”* (FGD, CBHI member)
	3.2. Payment delays	Service disruption due to financial issues	Barrier	*“Health facilities frequently withhold services from CBHI beneficiaries during short periods of financial processing.” (KII, city administration health office)*
	3.3. Coercive enrollment practices	Involuntary enrollment	Barrier	*“Different people use different ways that they think are efficient, and this goes out of the voluntarism lane.”* (KII, participant from hospital)
4. Service delivery	4.1. Access to services	Unequal treatment between paying & CBHI members	Barrier	*“If medications must be there, then they need to provide those drugs.”* (FGD, CBHI member)
	4.2. Quality of care	Conditional promotion of the program	Barrier	*“How can a person who has enrolled but doesn’t have service encourage others to join?”* (FGD, CBHI member)
5. Financial management	5.1. Mobilization issues	Delayed payments & financial strain	Barrier	*“The main problem of CBHI is the payment system.”* (KII, informant from hospital)
	5.2. Lack of transparency	Poor understanding of where payments go	Barrier	*“We do not know where it goes or how it functions?”* (FGD, CBHI member)
6. CBHI outcomes	6.1. Health & economic outcomes	Improved access to healthcare	Enabler	*“Rich people can pay whatever is asked, but those who cannot afford it are happy because they can receive healthcare.”* (KII, community leader)
	6.2. Women’s autonomy	Empowerment in healthcare decisions	Enabler	*“Any woman having a CBHI ID can visit a health facility without the will of anybody.”* (KII, CBHI focal)
7. Suggestions for improvement	7.1. Health system suggestions	Improved financial systems & accountability	Enabler	*“Financial management should be improved. Starting from the premium collection, it should be digitalized.” (KII, EPS participant)*
	7.2. CBHI scheme suggestions	Continuous awareness creation	Enabler	*“Awareness creation should be with information & examples.”* (KII, EPS Participant)

#### Main themes and categories of community-based health insurance enrollment.

***Community knowledge and perceptions of CBHI*:** Three subthemes emerged: awareness, misconceptions, and the perceived necessity of CBHI. Most community members reported increased awareness, but misconceptions remained prevalent.

***Awareness and misconceptions of CBHI***: Nine of twelve focus group participants noted that awareness of CBHI had improved. Most were familiar with eligibility criteria and benefits, but nearly all lacked knowledge of the legal and governance framework.

*“We know that it is useful, and we know who enrolls and who can’t.”* (FGD, 45-year-old women)

Misconceptions included beliefs that CBHI is solely for the poor or that participation implied planning for illness.

*“Some respondents equate CBHI with illness planning, assumed it is exclusively for the poor, and believed that non-usage equates to a loss in value.”* (FGD, 50-year-old woman CBHI member)

Factors driving misconceptions included misinformation, limited transparency, poor communication, unrealistic expectations, and the campaign-based nature of CBHI initiatives. Community awareness efforts employed forums, religious institutions, and door-to-door campaigns.

*“CBHI has a lot of benefits for those who don’t have much money, but it’s not in a good picture right now in our kebele.”* (FGD, 55-year-old male CBHI member)

Historical perceptions of public facilities contributed to negative attitudes, with poorer and chronically ill populations disproportionately enrolled.

*“Why are only poor and chronically ill people members of CBHI? It is because public hospitals are viewed as unclean and poorly equipped, meant for the underprivileged, compared to private facilities.”* (KII, 46-year-old)

*“Sometimes they think that if they have money, then joining CBHI is unnecessary. This stems from awareness problems, as they consider CBHI to be meant for the poor.”* (KII, 39-year-old male)

Misconceptions also extended to health facility staff, who sometimes perceived CBHI members as free-service users, increasing the risk of dropout.

*“There is a belief that CBHI members are free users or perceived as donation beneficiaries... they prioritize cash payers and treat CBHI members as if they receive free services. Such disparities in treatment can increase dropout rates.”* (KII, 44-year-old male)

***Perceived Necessity of CBHI***: Despite misconceptions, nearly all participants recognized CBHI as essential for reducing the financial burden of healthcare, improving timely access, and reducing reliance on home remedies.

*“CBHI has been very useful for us and will continue to be useful in the future.”* (FGD, 58-year-old male)

*“Since 2013, we have been beneficiaries. Visiting a private health facility would cost us a lot, but since CBHI started, we only pay once annually and can use health services for free whenever we need them.”* (FGD, 62-year-old male)

#### CBHI program design and implementation.

Four subthemes emerged: structure and governance, service delivery, enrollment and retention, and financial management.

***Structure and governance***: Participants reported an inconsistent understanding of CBHI governance, reflecting ambiguity and limited transparency. Some viewed it as a government political initiative; others attributed oversight to the Woreda administration or regional health bureau. All acknowledged the presence of a board of directors.

*“The CBHI is a political agenda led by regional and zonal party leaders.”* (KII, 35-year-old male)

*“This complex arrangement contributes to erroneous claims and accountability issues within the health system.”* (KII, 40-year-old male)

***Accountability mechanisms***: Accountability was limited by infrequent board meetings and insufficient staffing at the kebele and community levels. The dual role of the health sector as service provider and purchaser further undermined efficiency.

*“The current CBHI structure lacks sufficient staffing, especially at the kebele level.”* (KII, 43-year-old male)

*“Currently, it employs three staff at the woreda level: a coordinator, a finance expert, and a data clerk. However, the organizational structure lacked extension into the community and struggled with insufficient human resources.”* (KII, 37-year-old male)

*“The health sector also both provides and purchases health services, leading to inefficiencies and a lack of accountability.”* (KII, 34-year-old male)

***Enrollment and retention***: Enrollment barriers included unequal treatment at health facilities, poor drug and lab service availability, exempted services, payment delays, and coercive enrollment practices.

*“They don’t treat people who identify as CBHI members the same as cash payers. Medicines for CBHI users are sourced from private pharmacies when they should be available at health facilities. The issue is not the government’s fault but lies at the health facility level.”* (FGD, 54-year-old male)

*“Everyone has their methods for what they perceive to be effective, steering away from the principles of voluntary enrollment. Their main focus is fulfilling the expected enrollment quotas.”* (KII, 44-year-old male)

Moral hazard concerns, such as the sale of CBHI-provided medications, were reported.

*“While working in outpatient care, a patient I treated five days prior returned, falsely claiming a relapse of the disease to obtain more medication. Finally, we discovered that he was selling his medication as a source of income.”* (KII, 48-year-old male)

***Service delivery***: Most participants reported poor service delivery, though some had positive experiences. Dissatisfaction was common among those accessing basic primary care without adequate drugs or lab services, whereas chronic patients using referral services were more satisfied.

*“Community members will admire the system if they beneficially utilize it. Conversely, they could amplify negative sentiments if they do not receive satisfactory services.”* (KII, 53-year-old male)

*“For instance, a man diagnosed with benign prostatic hyperplasia (BPH) was admitted for surgery for thirty days under the CBHI. However, he purchased medications costing 4,000 birr, after which he disseminated misinformation as if CBHI failed to cover his cost.”* (KII, 34-year-old male)

***Financial mobilization and management***: Financial management challenges included delayed fund transfers, misappropriation of CBHI payments, and a lack of transparency, which impacted access to services.

*“The management of CBHI finance is poor, including delays in fund transfers to health facilities and misappropriation of CBHI payments.”* (FGD, 59-year-old male)

*“Payments were delayed, with some woredas taking six months to a year to settle, impacting the availability of medication, laboratory reagents, and services provided.”* (KII, 44-year-old male)

*“Even with an expectation of a 25% profit on loans, many health facilities struggle with defaults due to non-payment from woredas or regions.”* (KII, 39-year-old male)

***Grievance mechanisms***: Over a quarter of users reported a lack of grievance mechanisms or unawareness of complaint procedures.

*“We couldn’t locate any responsible person to voice our complaints.”* (FGD, 57-year-old male)

*“There is no designated individual accountable for the management of the scheme, leaving us without recourse.”* (FGD, 58-year-old male)

#### CBHI outcomes.

***Health and economic benefits***: Participants reported improved healthcare access, reduced out-of-pocket expenditures, and increased utilization, particularly among women and low-income households.

*“CBHI relieves the economic burden associated with illnesses by providing financial protection.”* (FGD, 56-year-old female)

*“Since 2013, we have been beneficiaries. Would it have cost us a fortune to seek treatment elsewhere? Thanks to CBHI, we contribute once and receive services without additional charges throughout the year. I had a child with a broken leg who spent 15 days in the hospital, and we incurred no additional expenses.”* (FGD, 61-year-old community-leader)

***Women’s autonomy***: CBHI enhanced women’s decision-making for health-seeking, allowing them to access services independently.

*“Before joining CBHI, we had to ask our husbands for money whenever our children or we got sick; now, we can just grab our CBHI card and go to the health facility.”* (FGD, 53-year-old female)

*“With a CBHI ID, any woman can visit a health facility without relying on anyone else... In rural areas, husbands may have multiple wives, but CBHI allows them to seek care independently.”* (KII, 54-year-old female)

*“CBHI provides women with the freedom to access medical services without waiting for approval from husbands or others since their expenses are already covered.”* (FGD, 46-year-old female)

#### Key barriers to community-based health insurance enrollment in the Sidama Region of Ethiopia in 2024.

Table 3 outlines the key barriers to community-based health insurance (CBHI) enrollment in the Sidama region of Ethiopia in 2024. First, misconceptions about CBHI being exclusively for the poor discourage broader enrollment and perpetuate stigma. Second, governance issues and a lack of clear structures at the *kebele* level confused roles and accountability, undermining the program’s efficacy (Table 3).

**Table 3:** Summary table of key barriers identified on CBHI enrollment in Sidama region, Ethiopia, 2024.

Barrier	Description
Misconceptions regarding CBHI	Many believe it’s only for the poor & signals illness.
Governance & structural issues	Confusion over governance structure & lack of accountability.
Discrimination in service delivery	Increased priority is given to cash-paying patients.
Service delivery inadequacies	Unavailability of required drugs & services.
Payment delays	Payment delays erode trust & disrupt services.
Lack of transparency	Decisions are not engaging communities at a local level
Unequal treatment of between paying & CBHI members	Paying patients receive priority at the point of encounter
Lack of clarity in accountability	Poor understanding of where payments go.

Third, discrimination in service delivery, manifesting as preferential treatment for cash-paying patients, exacerbates inequity and dissuades CBHI utilization. Fourth, inadequacies in service delivery, particularly the lack of essential drugs and laboratory services, reduce trust in the healthcare system. Lastly, payment delays created barriers to access, further eroding trust. Collectively, these barriers highlighted the urgent need for targeted interventions focused on education, structural reform, equitable service delivery, and efficient financial management to enhance the effectiveness and accessibility of CBHI in the region.

#### Key enablers to community-based health insurance enrollment in the Sidama Region of Ethiopia, 2024.

Table 4 details critical enablers facilitating the implementation and uptake of Community-Based Health Insurance (CBHI) in the Sidama region of Ethiopia. Each enabler enhances the program’s effectiveness and its impact on the community. First, an increase in awareness surrounding CBHI indicated a boost in community knowledge about its structure and benefits, which is essential for capable healthcare decisions. This expanded awareness is likely to enhance enrollment and utilization while addressing misconceptions and reinforcing trust (Table 4).

**Table 4:** Summary table of key enablers identified on CBHI enrollment in Sidama region, Ethiopia, 2024.

Enabler	Description
Enhanced awareness of CBHI	Improved community knowledge about the benefits of CBHI.
Recognized the necessity of the program	Acknowledgement that CBHI is essential for most families.
Empowerment of women	CBHI allows women to seek healthcare independently.
Positive health outcomes	Reduced financial burden & increased access to healthcare.
Ongoing community engagement	Suggested continuous awareness efforts to boost enrollment

Additionally, the recognized necessity of the program highlighted the community’s perception of CBHI as a vital resource, enhancing healthcare access and financial security. Recognizing CBHI’s crucial role in mitigating healthcare costs, particularly in scenarios where out-of-pocket payments pose challenges, is pivotal. Furthermore, the empowerment of women signifies a positive transformation in gender dynamics, allowing women to seek healthcare independently as formal consent from male figures becomes unnecessary, consequently improving health outcomes for themselves and their families.

Moreover, positive health outcomes associated with CBHI enrollment illustrated reduced financial burdens and heightened healthcare access. This financial protection allows the timely utilization of health services without the anxiety of incurring excessive debt, promoting an overall improvement in health. Finally, the focus on continuous community engagement emphasizes the necessity of sustained outreach initiatives to elevate and maintain CBHI enrollment. Regular engagement ensures that community comprehension of CBHI remains pertinent, addressing misconceptions while reinforcing program benefits.

## Discussion

The findings from this study elucidated various barriers and enablers affecting community-based health insurance (CBHI) enrollment in the Sidama region of Ethiopia while drawing parallels with global experiences. The discussion can benefit from a more analytical lens that connects the findings to established frameworks such as health system strengthening and behavioral change theories, notably the Theory of Planned Behavior (TPB). The framework of health system strengthening, defined as the process of enhancing the overall performance of health systems to ensure the delivery of quality and accessible healthcare, serves as a foundation for improving health outcomes [34] it can better comprehend the barriers to CBHI enrollment observed in the Sidama region, Ethiopia. Barriers such as governance failures, inequitable treatment of CBHI members, and recurrent medication stockouts serve as indicators of a compromised health system. These inefficiencies hinder the operations of CBHI programs, as evidenced by the low enrollment rates and dissatisfaction expressed by study participants.

A barrier identified in this study was the misconception that CBHI primarily serves economically disadvantaged populations, which appears to limit enrollment across diverse socioeconomic strata. This phenomenon is consistent with findings from Kenya, where associations between community-based health insurance and poverty deter higher-income individuals from enrolling, thus perpetuating health inequities [35]. Conversely, in countries like Ghana, targeted community campaigns successfully shifted perceptions, leading to increased enrollment across various income groups [36]. Such comparative evidence suggests that addressing societal perceptions of community-based health insurance schemes is critical for promoting inclusivity. Furthermore, governance and accountability issues surfaced as critical barriers in Sidama, Ethiopia, mirroring challenges documented in Uganda, where poor governance undermines public trust in enrollment in community-based health insurance schemes [37]. This comparative evidence underscores the importance of addressing societal perceptions to promote inclusivity in CBHI schemes.

In the Sidama region, study participants also expressed confusion regarding the roles of stakeholders, which resonates with findings from Uganda, where community-based health insurance members reported uncertainty regarding the roles of local community-based insurance committees [38]. Meanwhile, in Ghana, robust governance structures have been linked to improved stakeholder confidence and enhanced program efficacy. Thus, the contrasting experiences highlight that clear governance can serve as a key enabler in some contexts, while a lack of it proves to be a barrier in others [39]. In addition to governance issues, inequitable treatment of CBHI members within health facilities emerged as another barrier. Study participants in Sidama indicated that cash-paying patients often received preferential treatment, which aligns with findings from South Africa, where disparities in service delivery negatively impact trust in community-based health insurance schemes [40]. In contrast, successful models in countries like Rwanda, where community-based health insurance has been widely embraced, have demonstrated better integration of CBHI members into the healthcare system, leading to improved service delivery outcomes [41]. Thus, while inequitable treatment constitutes a barrier in some contexts, effective implementation of CBHI can facilitate trust and better treatment outcomes in others [42].

Another important barrier is the existence of chronic medication, laboratory services stockouts, and reimbursement delays for providers within the Ethiopian CBHI, which also resonates with the challenges faced in community-based health programs in many African nations [43,44]. Reports of stockouts and delays can compromise the sustainability of community-based health insurance schemes [45], whereas evidence from Mexico suggests that efficient supply chain management and timely reimbursements positively influence patient satisfaction and retention within community-based health insurance programs [46]. Therefore, the disparities in experiences related to operational efficiency underscore the need for context-specific solutions to enhance CBHI efficacy.

Simultaneously, the Theory of Planned Behavior (TPB) provides a valuable lens for understanding the enablers of CBHI enrollment, including heightened awareness of the benefits of CBHI and the financial protection it offers. According to the TPB, the intention to engage in a specific behavior is influenced by an individual’s attitudes, subjective norms, and perceived behavioral control [47]. In the context of CBHI enrollment, increased awareness and the perception of financial protection can foster a more favorable attitude towards participating in CBHI, subsequently influencing an individual’s intention to enroll. Thus, interventions aimed at enhancing awareness and the perception of financial security possess the potential to effectively promote CBHI enrollment when viewed through the framework of TPB.

In line with this, several enablers facilitating CBHI enrollment were identified, with increased awareness around CBHI benefits being a crucial factor. This finding is consistent with studies in Tanzania, which demonstrated that community education can dismantle misconceptions about community-based health insurance, leading to higher enrollment rates [48]. Educational initiatives that have been successful in countries like India have also demonstrated that increasing understanding of the utility of community-based health insurance can impact enrollment [49]. Additionally, the financial protection afforded by CBHI emerged as an essential enabler, echoing findings from Ghana and the Philippines, where respondents recognized the role of community-based health insurance in alleviating substantial out-of-pocket healthcare costs [50,51]. In Brazil, a well-established public health insurance system has similarly shown that effective financial protection significantly improved healthcare access and reduced the economic burden on families [52]. Consequently, such comparative evidence illustrates the critical connection between financial security and health service utilization across diverse global contexts.

Another notable enabler is the empowerment experienced by women through CBHI enrollment, which aligns with research from South Africa. Study participants reported a greater ability to access healthcare without needing spousal consent, thus fostering improved health-seeking behaviors [53]. In contrast, in many low- and middle-income countries where women experience barriers to accessing healthcare, the lack of community-based health insurance coverage exacerbates gender disparities in health outcomes [54]. Thus, while CBHI programs may promote female agency in some contexts, they may also highlight the continued gender inequities in access to healthcare in others. Additionally, the evidence of positive health outcomes linked with CBHI enrollment points to another vital enabler [55].

Study participants in Sidama articulated that enrollment led to reductions in out-of-pocket expenditures and increased access to healthcare services. This resonates with findings from Ghana, where community-based health insurance enrollment positively influenced healthcare access [56]. Moreover, in Rwanda, the successful integration of community-based health insurance has contributed to increased healthcare utilization and better health indicators, suggesting that enrollment can yield tangible benefits when programs are effectively executed. Thus, the contrasting experiences underlining the role of CBHI in enhancing access and outcomes emphasized the importance of optimizing program execution to achieve desired health impacts [41].

Finally, ongoing community engagement and education emerged as critical enablers for sustaining CBHI enrollment. The findings suggested that regular interaction through forums, religious gatherings, and door-to-door outreach fosters trust and responsiveness. A study from Kenya reinforces this observation, demonstrating that community involvement enhances the success of community-based health insurance initiatives [57]. In contrast, in settings where community voices are not integrated into decision-making processes, community-based health insurance programs often fail to achieve their intended objectives. Thus, while community engagement is a vital enabler in some contexts, its absence may serve as a barrier in others [58,59].

### Limitations of the study

This study explored the barriers and enablers of community-based health insurance (CBHI) enrollment in the Sidama National Regional State has several inherent limitations that must be acknowledged to contextualize the findings. Firstly, the dependence on qualitative methodologies, including interviews and focus group discussions, may introduce various biases, particularly self-reporting bias. Individuals may alter their responses due to social desirability or perceived expectations from researchers, potentially resulting in an overrepresentation of favorable perspectives [60] on CBHI and an underreporting of existing barriers.

Secondly, the geographical specificity of this study to the Sidama region limits the generalizability of the findings to other regions within Ethiopia or other low- and middle-income countries (LMICs) [61]. The socio-cultural and economic conditions particular to Sidama may not reflect the experiences of communities in other areas, thus constricting the broader applicability of the results to different contexts.

Additionally, the purposive sampling method employed may have introduced selection bias [62]. Participants who are more positively inclined toward or engaged with CBHI may have been overrepresented, potentially neglecting the voices of those who face considerable barriers or hold negative perceptions of CBHI. This limitation constrains the diversity of perspectives captured through this study.

Moreover, the cross-sectional design of the study confines the analysis to a specific point in time [63], restricting the ability to observe changes in attitudes or behaviors regarding CBHI enrollment over time. Longitudinal studies could provide deeper insights into how evolving community dynamics and external factors influence perceptions and participation in CBHI initiatives. Furthermore, the potential for cognitive biases and misunderstandings regarding the complexities of community-based health insurance mechanisms may have impacted the reliability of participants’ responses. Variability in individual interpretations of interview questions about CBHI could lead to discrepancies in the data collected, thereby affecting the overall findings [64].

In addition to these limitations, it should be acknowledged that language barriers may have influenced the data collection process, particularly when participants lacked full fluency in the language employed during interviews or focus group discussions. Such barriers may have compromised the depth and clarity of responses [65]. Finally, interviewer bias represents a further methodological concern, as the perspectives or expectations of interviewers could have inadvertently shaped participants’ responses or the interpretation of qualitative data. Transparent acknowledgment of these issues strengthens the trustworthiness and credibility of the study’s findings [60].

### Strengths of the study

Despite the aforementioned limitations, this study possesses distinct strengths that contribute to its scholarly value. The qualitative approach allowed for an in-depth exploration of community perceptions regarding the barriers and enablers of CBHI enrollment, capturing detailed experiences and insights that quantitative methodologies might overlook. The involvement of a diverse range of study participants, including CBHI members, non-members, community leaders, and healthcare providers, strengthened the quality of the data collected. This approach provided a comprehensive understanding of the issue under investigation.

The application of the Theory of Planned Behavior as a conceptual framework further enhances the robustness of the study. This theoretical lens elucidates how attitudes, perceived norms, and perceived behavioral control inform individual decision-making related to CBHI enrollment, thereby offering actionable insights for policymakers to design targeted interventions aimed at increasing enrollment rates.

Additionally, the study’s focus on local cultural dynamics and specific community experiences adds significant value to the existing literature on community-based health insurance in LMICs. By highlighting localized barriers and enablers to CBHI enrollment, this study enriches the understanding of the unique factors influencing community-based health insurance uptake in similar contexts.

## Conclusion

This study identified key barriers and enablers to community-based health insurance (CBHI) enrollment in Sidama National Regional State, Ethiopia. The study participants acknowledged the importance of CBHI in providing financial protection and enhancing access to healthcare. However, misconceptions, governance challenges, and poor service delivery limited enrollment. Therefore, targeted community education, transparent governance, and improved service quality are essential. Moreover, these findings offer valuable insights for similar low-income settings. Therefore, context-specific strategies are needed to strengthen health systems and increase CBHI enrollment and retention.

## Supporting information

S1 Checklist The COREQ checklist. (PDF)

S2 File The open-ended focus groups discussion (FGDs) and key informants’ interview (KIIs) guides. (DOCX)

S3 Text The verbatim transcription of all audio recordings from the FGDs and KIIs. (DOCX)

S4 Checklist The Inclusivity in Global Research format (template). (DOCX)
