# Supplementary material for: Barriers and enablers of community-based health insurance enrollment in the Sidama national regional state, Southern Ethiopia, 2024: A qualitative study
Source: PLOS Glob Public Health. 2025 Sep 11;5(9):e0004310. doi: 10.1371/journal.pgph.0004310 (PMC12425181; doi:10.1371/journal.pgph.0004310)
Supplement: S2 File — (DOCX) [file pgph.0004310.s002.docx]

**Annexes**

1. **Focus group discussion guide (English version)**

**Moderator Introduction:** Hello, my name is [NAME], and I am [POSITION]. Thank you for participating in this interview today. This study aims to identify the barriers and enablers of CBHI from stakeholders from the Sidama region, Ethiopia, with the ultimate goal of enhancing their health status by facilitating access to and utilization of healthcare services. I am seeking your opinions and views on the implementation status of community-based health insurance in the Sidama region.

The information and data collected will be used for study purposes only.

You are selected as a potential participant in this study as a subject.

**Potential risks:** There will be no potential risks that may cause any harm to study participants.

**Benefits:** There are no financial benefits associated with this study. However, by taking part in this study, you may contribute to improving the performance of community-based health insurance among women's groups.

**Confidentiality:** All information given by you will be kept strictly confidential. Your participation shall be voluntary, and not obligated to answer any question that you do not wish to answer. If you feel any discomfort with the questionnaire, please feel free to drop it at any time. This questionnaire will take approximately 60 to 90 minutes.

Are you willing to participate voluntarily? (1) Yes, (0) No. If you agree, I will use the audio recording. (1) Okay, (0) No.

**Written consent from participants**

Hawassa University

I have understood all about the objective and the process of the study. My participation is voluntary and not obligated to answer any question that I do not know or do not wish to answer. I also understood that all information given to me will be kept strictly confidential. Therefore, I am willing to participate in this study.

Study participant sign _____________________________ date _____________

Data collector name: _________________________________________Signature _________

Date of interview ----------- Month --------------- /2016 E. C.

Supervisor’s Name ___________________________________________ Signature________

Checked date________________ /2016 E.C.

Contact Address of the Principal Investigator

Name: XXXX

E-Mail: [kare.debessa@gmail.com](mailto:abigiatenaw@gmail.com)

1.1. Identification of Focus group discussants (English version)

| I | Section I: Identification | |
| --- | --- | --- |
| 1 | Questionnaire ID | ____________________________ |
| 2 | Name of Woreda/City | ____________________________ |
| 3 | Name of Kebele | ____________________________________ |
| 4 | Name and signature of a moderator | _______________________________ |
| 5 | Name and signature of note-taker | _________________________________ |
| 6 | Date of discussion | _______________________________ |
| 7 | Start time: | ______________ |
| 8 | End time: | __________ |

- 1. Participant demographic intake sheet

| FGD Participant’s identification code | | Demographic information | | | | | |
| --- | --- | --- | --- | --- | --- | --- | --- |
|  |  | Age | Religion | Marital status | Are you employed? (Yes/No) | Educational level | Remarks |
| 1 | A1 |  |  |  |  |  |  |
| 2 | A2 |  |  |  |  |  |  |
| 3 | A3 |  |  |  |  |  |  |
| 4 | A4 |  |  |  |  |  |  |
| 5 | A5 |  |  |  |  |  |  |
| 6 | A6 |  |  |  |  |  |  |
| 7 | A7 |  |  |  |  |  |  |
| 8 | A8 |  |  |  |  |  |  |
| 9 | A9 |  |  |  |  |  |  |
| 10 | A10 |  |  |  |  |  |  |
| 11 | A11 |  |  |  |  |  |  |
| 12 | A12 |  |  |  |  |  |  |

Focus group discussion guide for CBHI awareness, perception of affordability of the premium renewals for CBHI, the role of leadership, and quality of healthcare

1. Could you tell us what you know about CBHI?

Probes:

- CBHI enrollment,
- dropouts
- Premium contribution and its management
- Moral hazard (the tendency of insured individuals to overconsume healthcare services because the costs are covered by the CBHI insurance) How does this affect the CBHI?
- Adverse selection (high-risk individuals being more likely to enroll, leading to higher costs and premiums for the CBHI pool). How does this affect the CBHI?
- How about specifics related to women?

1. How does the community perceive the benefits of community-based health insurance?

Probe: What is the community’s perception of CBHI benefits, specifically concerning women?

1. What are the factors that you think can facilitate household enrollment and continued membership in community-based health insurance?

Probe: What about among women of childbearing age?

1. What are the barriers to enrollment in community-based health insurance?

Probe: What about among women of childbearing age?

1. What strategies could be implemented to increase enrollment in community-based health insurance?

Probe: Are there any strategies specific to women?

1. Can you describe the organizational structure of the CBHI in your community?

Probe: How are decisions made within this structure? Who holds the most influence or power?

1. How does the hierarchy within the CBHI affect its operations and effectiveness?

Probe: Are there any challenges or benefits associated with this hierarchy?

1. What are your thoughts on the leadership of the CBHI?

Probe: How does the leadership impact the functioning and success of the CBHI?

1. Is there anything you would like to add?

Thank you for your active participation!!!

**II: For the key informants' interview Outlines (English version)**

**Interviewer Introduction:** Hello, my name is [NAME], and I am [POSITION]. Thank you for participating in this interview today. I am seeking your opinions and views on the implementation status of community-based health insurance in the Sidama region. The information and data collected will be used for study purposes only.

You are selected as a potential participant in this study as a subject.

**Potential risks:** There will be no potential risks that may cause any harm to study participants.

**Benefits:** There are no financial benefits associated with this study. However, by taking part in this study, you may contribute to improving the performance of community-based health insurance among women's groups.

**Confidentiality:** All information given by you will be kept strictly confidential. Your participation shall be voluntary, and not obligated to answer any question that you do not wish to answer. If you feel any discomfort with the questionnaire, please feel free to drop it at any time. This questionnaire will take approximately 60 to 90 minutes.

Are you willing to participate voluntarily? (1) Yes, (0) No. If you agree, I will use the audio recording. (1) Okay, (0) No.

**Written consent from participants**

Hawassa University

I have understood all about the objective and the process of the study. My participation is voluntary and not obligated to answer any question that I do not know or do not wish to answer. I also understood that all information given to me will be kept strictly confidential. Therefore, I am willing to participate in this study.

Study participant sign _____________________________ date _____________

Data collector name: _________________________________________Signature _________

Date of interview ----------- Month --------------- /2016 E. C.

Supervisor’s Name ___________________________________________ Signature________

Checked date________________ /2016 E.C.

Contact Address of the Principal Investigator

Name: XXXX

E-Mail: [kare.debessa@gmail.com](mailto:abigiatenaw@gmail.com)

V: Key informants interview identification (English version)

| I | Section I: Identification | |
| --- | --- | --- |
| 1 | Questionnaire ID | ____________________________ |
| 2 | Name of Woreda/City | ____________________________ |
| 3 | Name of Kebele | ____________________________________ |
| 4 | Name and signature of a moderator | _______________________________ |
| 5 | Name and signature of note-taker | _________________________________ |
| 6 | Date of discussion | _______________________________ |
| 7 | Start time: | ______:________ |
| 8 | End time: | ____:______ |

VI: Participant demographic intake sheet

| KIIs identification code | | Demographic information | | | | | | |
| --- | --- | --- | --- | --- | --- | --- | --- | --- |
|  |  | Age | Religion | Marital status | Educational level | Are you employed? (Yes/No) | Role/ position | Remarks |
| 1 | KII1 |  |  |  |  |  |  |  |
| 2 | KII2 |  |  |  |  |  |  |  |
| 3 | KII3 |  |  |  |  |  |  |  |
| 4 | KII4 |  |  |  |  |  |  |  |
| 5 | KII5 |  |  |  |  |  |  |  |
| 6 | KII6 |  |  |  |  |  |  |  |
| 7 | KII7 |  |  |  |  |  |  |  |
| 8 | KII8 |  |  |  |  |  |  |  |
| 9 | KII9 |  |  |  |  |  |  |  |
| 10 | KII10 |  |  |  |  |  |  |  |
| 11 | KII11 |  |  |  |  |  |  |  |
| 12 | KII12 |  |  |  |  |  |  |  |
| 13 | KII13 |  |  |  |  |  |  |  |
| 14 | KII14 |  |  |  |  |  |  |  |
| 15 | KII15 |  |  |  |  |  |  | |
| 16 | KII16 |  |  |  |  |  |  | |
| 17 | KII17 |  |  |  |  |  |  | |
| 18 | KII18 |  |  |  |  |  |  | |
| 19 | KII19 |  |  |  |  |  |  | |
| 20 | KII20 |  |  |  |  |  |  | |

**Questions for *kebele*/health facility, woreda, city, or regional level leaders:**

.

1. Please tell me about the sector you work in.

A. Government at the regional, zonal, woreda, or city administration level or health facility

B. International non-governmental organizations.

1. Could you tell me what you know about CBHI?

Probes:

- CBHI enrollment, dropout
- Premium and its management
- Moral hazard (the tendency of insured individuals to overconsume healthcare services because the costs are covered by the CBHI insurance)
- Adverse selection (high-risk individuals being more likely to enroll, leading to higher costs and premiums for the CBHI pool)
- How about specifics related to women?

1. How does the community perceive the benefits of community-based health insurance?

Probe: What is the community’s perception of CBHI, specifically concerning women?

1. What are the factors that you think can facilitate household enrollment and continued membership in community-based health insurance?

Probe: What about among women of childbearing age?

1. What are the barriers to enrollment in community-based health insurance?

Probe: What about among women of childbearing age?

1. What strategies could be implemented to increase enrollment in community-based health insurance?

Probe: Are there any strategies specific to women?

1. Can you describe the organizational structure of the CBHI in your community?

Probe: How are decisions made within this structure? Who holds the most influence or power?

1. How does the hierarchy within the CBHI affect its operations and effectiveness?

Probe: Are there any challenges or benefits associated with this hierarchy?

1. What are your thoughts on the leadership of the CBHI?

Probe: How does the leadership impact the functioning and success of the CBHI?

1. Is there anything would you like to add?

**For nonmembers and dropped-out individuals**

1. Tell me what do you know about CBHI?
2. Have ever dropped out from the CBHI? Tell me the main reasons that made you drop out.
3. would you like to rejoin the CBHI again? If not, tell me the main reasons that hinder you not to re-join CBHI.
4. Tell me the main barriers that hinder you not to enrolling in CBHI.

How about specifics related to women?

1. Do you have anything to add?

Thank you for your participation!

List of interviewees

| KII Code | Institutions or individuals to be interviewed |
| --- | --- |
| KII1 | International non-governmental organization (NGO) |
| KII2 | Health Insurance Agency, Sidama regional branch |
| KII3 | Sidama National Regional Health |
| KII4 | Sidama National Regional Health |
| KII5 | Sidama National Regional Health |
| KII6 | Dale woreda administration |
| KII7 | Dale woreda administration |
| KII8 | Yirgalem city administration |
| KII9 | Yirgalem city administration |
| KII10 | Best performing kebele leader |
| KII11 | Best performing kebele leader |
| KII12 | Least performing kebele leader |
| KII13 | Least performing kebele leader |
| KII14 | Best performing kebele community leader |
| KII15 | Least performing kebele community leader |
| KII16 | Dropped out household head |
| KII17 | Dropped out household head |

*KII- key informants’ interview
