# Supplementary material for: Barriers and enablers of community-based health insurance enrollment in the Sidama national regional state, Southern Ethiopia, 2024: A qualitative study
Source: PLOS Glob Public Health. 2025 Sep 11;5(9):e0004310. doi: 10.1371/journal.pgph.0004310 (PMC12425181; doi:10.1371/journal.pgph.0004310)
Supplement: S3 Text — (DOCX) [file pgph.0004310.s003.docx]

**FGD1: Focus Group Discussion One**

**M:** For transcription purposes, we’ll be recording our conversation, is that OK?

**D:** Yes, it is OK. No problem.

**M:** Thank you for joining us today. We're eager to delve into the intricate workings of Community-Based Health Insurance (CBHI) with your expertise. Firstly, could you shed some light on CBHI enrollment trends and the challenges associated with dropouts from the program? Moving on to the financial aspect, could you elaborate on premium contribution mechanisms and how they're managed within the CBHI framework? We've heard discussions regarding moral hazard, wherein insured individuals may overconsume healthcare due to covered costs; could you outline its impact on CBHI operations? Additionally, how does adverse selection, where high-risk individuals are more inclined to enroll, affect the CBHI pool and its sustainability? Lastly, could you provide insights into specific considerations related to women within the CBHI context? Your expertise in navigating these nuanced aspects would greatly enrich our understanding.

**D2:** Since 2013, we have been beneficiaries. For medical treatment, going to another place would cost us a lot of money, but since CBHI came, we pay 600 birr and after we pay once, we can always use it for free around year. The one-time expense we would have incurred and the one we go to another place for, here, we can use it all year round. I had a child who had a broken leg, without spending any money; he was bedridden for 15 days, including all expenses. I have encouraged others to use it. People in this area have a good attitude towards CBHI.

**M: Others?**

**D1:** Many people used CBHI. However, I am not a CBHI beneficiary because I am a government employee; I thought it would be good if government employees were seen. Even if we add a little money, we also become beneficiaries. In addition, a lot of money has been added this year. It started with 400 birr, then immediately entered 600 birr. In the end, those called government poor people will want to use it; CBHI was only being given to the designated person in the district.

**M: Others?**

**D3:** As I said earlier, there is nothing else: if a person who has enrolled and uses CBHI, has to encourage others to enroll in CBHI, how can a person who has enrolled and does not get the service and has money issues encourage others?

**M:** Could you provide insights into how the community views the advantages of CBHI overall? Additionally, we're keen to understand specifically how women within the community perceive the benefits of CBHI. Their perspectives are vital for understanding the nuanced impact of CBHI initiatives on women's health and well-being. Your expertise in elucidating these perceptions will undoubtedly contribute to our understanding of the broader implications of CBHI on community health outcomes.

**D4:** When the CBHI started in this kebele, I was also a beneficiary here. The other one is because of the increase in money, we are forced to go to other places and get treated with our, before this, we could only get treated at health centers and hospitals here. The service is good and the community has accepted it, it is being used widely. The previous contribution has been increased by 200 birr.

**D8:** it is particularly important for women and children because they are vulnerable to many health problems.

**D9:** kebele leaders and health extension workers are encouraging the community to become members of CBHI but the community is not giving due attention to this important scheme.

**M:** Could you share your insights on the key elements that encourage households to enroll and maintain their membership in CBHI programs? Additionally, we're particularly interested in understanding these factors within the context of women of childbearing age. Their unique healthcare needs and roles within households underscore the importance of tailored strategies to ensure their active participation and continued coverage within CBHI schemes. Your expertise in identifying these facilitators will greatly inform efforts to enhance CBHI uptake and retention, particularly among this demographic.

**D9:** as I told you earlier, kebele leaders and health extension workers are encouraging the community to become members of CBHI but the community is not giving due attention to this important scheme.

**D4:** the service our neighbors obtained from CBHI motivates us to enroll.

**D5:** It’s a benefit

**M:** Could you please share your insights on the barriers to enrollment in community-based health insurance? Specifically, could you elaborate on the challenges faced by women of childbearing age in accessing and enrolling in such insurance programs?

**D1:** one thing is a restriction of government employees not to become members.

**D7:** discrimination during service provision for members and those who pay out of their pocket.

**M:** What strategies could be implemented to increase enrollment in community-based health insurance? Additionally, are there any strategies that could be specifically targeted toward increasing enrollment among women?

**D5:** When it started, it was a fair contribution of money and people used it happily. As it is known, the cost of living is also increasing. When the people have a problem, when they tell the government about the problem, it will be fixed immediately. The government has established a new system called the “Safety Net program”, where one person without money can hold 6 people so that everyone in that household can get services.

**D6:** CBHI has many benefits. However, the health professionals said that there is no medication in health centers, but now the government has solved that problem and is in a good situation. We are also encouraging many people to enroll. People say that it is a good desire and understanding that even if there is a shortage of medicine, it is a big thing to cover only the cost of treatment. But what is becoming a problem for new arrivals is that when they go to the health center for treatment, the health center officials say that their money has not been transferred and we will not provide services until it is transferred.

**M:** You said that the money is not properly collected and transferred to health facilities in a timely manner so where are the problems?

**D7:** In the past, we used a lot for 450 birr, but recently I renewed it for 650 birr, and when I went to a health center to be treated when I feel the pain they said that the money had not been transferred into our account from your kebele, so I was told to wait until it transferred in. due to this reason our kebele community is delaying in utilizing service.

**D8:** I used to get treatment from other organizations because my children were in the “compassion”, but recently my children have reached the age and then left the compassion, so I was saying that I will pay CBHI. Even if CBHI service is good some people who did not get the medication, drugs, and laboratory service come back home and say bad things about CBHI.

**M:** Do you know the organizational structure of CBHI? where does the money collected from you go? Who is responsible for doing this?

**D8:** We pay our money yearly here (at *Kebele*) but we don’t know where it goes and how it functions.

**D9:** I am a CBHI user of my family, although it is difficult to pay 600 birr for one person at a time, the benefits are many, so I use it happily. If we want treatment, we get free medications for the whole family. By using CBHI we have a chance to treat the children we are raising other than our biological children. So CBHI saved a lot of money and it has many benefits.

**D10:** CBHI has many benefits but some people have a little lack of awareness. Many people do not want to hear about it before anything happens to them and want to enroll when their family members or they become sick. My complaint is that this year, poor people are returning with their CBHI books and being told that they will not be given services unless they contribute from their pocket. Think over how this group will contribute to the CBHI from their pocket because these people are very poor and previously government contributed for them but did not know. Besides that CBHI has many benefits. It is used by almost any person in this area

**M:** Do you mean the government stopped to pay for them?

**D10:** The donation is for everyone according to our previous knowledge. So this means everyone is benefiting from CBHI. But currently this year, the poor are not allowed to use it.

**M:** Are there CBHI members who go to health facilities without getting sick in this community?

**D3:** No, even if it's a small/minor pain, they will go to the pharmacy to get medicine, but no one goes there without being sick.

**D10:** If it's a minor illness, a person goes to a private hospital and uses it, but even if they go to a government hospital, they will get treated.

**M:** Okay, if there's anything else you'd like to add?

**D3:** Currently, the government's efforts to improve it are good, but if people don't work, they can't earn money. So a person needs to know that there is no free service without paying any cents.

**D11:** What I would like to add on the things that need to improve CBHI is that if we can pay in three or four terms, it would be good for the community to deposit money around September and October when they get money because at that time almost all people have money.

**D3:** I mean if they must be transferring our money on time and we can get treatment and medication on time.

**D4:** If the government thinks about stopping what is given to the poor, and providing the same treatment or service for CBHI users and non-CBHI users.

**D10:** The contribution is for everyone, so based on the agreement, everyone is using it. Now, the poor have been notified not to use it. Except for that, everyone is a beneficiary.

**M: Anything you want to add?**

**D12:** The district and regional level manager will negotiate with us to increase CBHI membership and service utilization but the problem is at the health center level.

**M:** Is there anything would you like to add?

**D4:** At first, they should train health professionals to provide a good service.

**D3:** Many people ask us, but no one does anything practical, thank you.

**M: any critical things that you would like to add?**

**D12:** No, thank you.

**M:** Thank you for your active participation!!!

**FGD2: Focus Group Discussion two**

**M:** For transcription purposes, I’ll be recording our conversation, is that OK?

**P:** Yes, it is OK. No problem.

**M:** Thank you for joining us today. We're eager to delve into the intricate workings of Community-Based Health Insurance (CBHI) with your expertise. Firstly, could you shed some light on CBHI enrollment trends and the challenges associated with dropouts from the program? Moving on to the financial aspect, could you elaborate on premium contribution mechanisms and how they're managed within the CBHI framework? We've heard discussions regarding moral hazard, wherein insured individuals may overconsume healthcare due to covered costs; could you outline its impact on CBHI operations? Additionally, how does adverse selection, where high-risk individuals are more inclined to enroll, affect the CBHI pool and its sustainability? Lastly, could you provide insights into specific considerations related to women within the CBHI context? Your expertise in navigating these nuanced aspects would greatly enrich our understanding.

**P2:** Since 2013, we have been beneficiaries. For medical treatment, going to another place would cost us a lot of money, but since CBHI came, we have paid 600 birr and after we pay once, we can always use it for free around year. The one-time expense we would have incurred and the one we go to another place for, here, we can use it all year round. I had a child who had a broken leg, without spending any money; he was bedridden for 15 days, including all expenses. I have encouraged others to use it. People in this area have a good attitude towards CBHI.

**M: Others?**

**P1:** Many people used CBHI. However, I am not a CBHI beneficiary because I am a government employee; I thought it would be good if government employees were seen. Even if we add a little money and we also become beneficiaries. In addition, a lot of money has been added this year. It started with 400 birr, then immediately entered 600 birr. In the end, those called government poor people will want to use it; CBHI was only being given to the designated person in the district.

**M: Others?**

**P3:** As I said earlier, there is nothing else: if a person who has enrolled and uses CBHI, has to encourage others to enroll in CBHI, how can a person who has enrolled and does not get the service and has money issues encourage others?

**M:** Could you provide insights into how the community views the advantages of CBHI overall? Additionally, we're keen to understand specifically how women within the community perceive the benefits of CBHI. Their perspectives are vital for understanding the nuanced impact of CBHI initiatives on women's health and well-being. Your expertise in elucidating these perceptions will undoubtedly contribute to our understanding of the broader implications of CBHI on community health outcomes.

**P4:** When the CBHI started in this kebele, I was also a beneficiary here. The other one is because of the increase in money, we are forced to go to other places and get treated with our, before this, we could only get treated at health centers and hospitals here. The service is good and the community has accepted it, it is being used widely. The previous contribution has been increased by 200 birr.

**P8:** it is particularly important for women and children because they are vulnerable to many health problems.

**P9:** kebele leaders and health extension workers are encouraging the community to become members of CBHI but the community is not giving due attention to this important scheme.

**M:** Could you share your insights on the key elements that encourage households to enroll and maintain their membership in CBHI programs? Additionally, we're particularly interested in understanding these factors within the context of women of childbearing age. Their unique healthcare needs and roles within households underscore the importance of tailored strategies to ensure their active participation and continued coverage within CBHI schemes. Your expertise in identifying these facilitators will greatly inform efforts to enhance CBHI uptake and retention, particularly among this demographic.

**P9:** as I told you earlier, kebele leaders and health extension workers are encouraging the community to become members of CBHI but the community is not giving due attention to this important scheme.

**P4:** the service our neighbors obtained from CBHI motivates us to enroll.

**P5:** It’s a benefit

**M:** Could you please share your insights on the barriers to enrollment in community-based health insurance? Specifically, could you elaborate on the challenges faced by women of childbearing age in accessing and enrolling in such insurance programs?

**P1:** one thing is a restriction of government employees not to become members.

**P7:** discrimination during service provision for members and those who pay out of their pocket.

**M:** What strategies could be implemented to increase enrollment in community-based health insurance? Additionally, are there any strategies that could be specifically targeted toward increasing enrollment among women?

**P5:** When it started, it was a fair contribution of money and people used it happily. As it is known, the cost of living is also increasing. When the people have a problem, when they tell the government about the problem, it will be fixed immediately. The government has established a new system called the “Safety Net program”, where one person without money can hold 6 people so that everyone in that household can get services.

**P6:** CBHI has many benefits. However, the health professionals said that there is no medication in health centers, but now the government has solved that problem and is in a good situation. We are also encouraging many people to enroll. People say that it is a good desire and understanding that even if there is a shortage of medicine, it is a big thing to cover only the cost of treatment. But what is becoming a problem for new arrivals is that when they go to the health center for treatment, the health center officials say that their money has not been transferred and we will not provide services until it is transferred.

**M:** You said that the money is not properly collected and transferred to health facilities on time so where is the problem?

**P7:** In the past, we used a lot for 450 birr, but recently I renewed it for 650 birr and when I went to a health center to be treated when I feel the pain they said that the money had not been transferred to our account from your *kebele*, so I was told to wait until it transferred in. due to this reason our kebele community is delaying in utilizing service.

**P8:** I used to get treatment from other organizations because my children were in the “compassion”, but recently my children have reached the age and then left the compassion, so I was saying that I will pay CBHI. Even if CBHI service is good some people who did not get the medication, drugs, and laboratory service come back home and say bad things about CBHI.

**M:** Do you know the organizational structure of CBHI? where does the money collected from you go? Who is responsible for doing this?

**P8:** We pay our money yearly in here (at kebele) but we don’t know where it goes and how it functions.

**P9:** I am a CBHI user of my family, although it is difficult to pay 600 birr for one person at a time, the benefits are many, so I use it happily. If we want treatment, we get free medications for the whole family. By using CBHI we have a chance to treat the children we are raising other than our biological children. So CBHI saved a lot of money and it has many benefits.

**P10:** CBHI has many benefits but some people have a little lack of awareness. Many people do not want to hear about it before anything happens to them and want to enroll when their family members or they become sick. My complaint is that this year, poor people are returning with their CBHI books and being told that they will not be given services unless they contribute from their pocket. Think over how this group will contribute to the CBHI from their pocket because these people are very poor and previously government contributed for them but did not know. Besides that CBHI has many benefits. It is used by almost any person in this area

**M:** Do you mean the government stopped to pay for them?

**P10:** The donation is for everyone according to our previous knowledge. So this means everyone is benefiting from CBHI. But currently this year, the poor are not allowed to use it.

**M:** Are there CBHI members who go to health facilities without getting sick in this community?

**P3:** No, even if it's a small/minor pain, they will go to the pharmacy to get medicine, but no one goes there without being sick.

**P10:** If it's a minor illness, a person goes to a private hospital and uses it, but even if they go to a government hospital, they will get treated.

**M:** Okay, if there's anything else you'd like to add?

**P3:** Currently, the government's efforts to improve it are good, but if people don't work, they can't earn money. So a person needs to know that there is no free service without paying any cents.

**P11:** What I would like to add on the things that need to improve CBHI is that if we can pay in three or four terms, it would be good for the community to deposit money around September and October when they get money because at that time almost all people have money.

**P3:** I mean if they must be transferring our money on time and we can get treatment and medication on time.

**P4:** If the government thinks about stopping what is given to the poor, and providing the same treatment or service for CBHI users and non-CBHI users.

**P10:** The contribution is for everyone, so based on the agreement, everyone is using it. Now, the poor have been notified not to use it. Except for that, everyone is a beneficiary.

**M: Anything you want to add?**

**P12:** The district and regional level manager will negotiate with us to increase CBHI membership and service utilization but the problem is at the health center level.

**M:** Is there anything would you like to add?

**P4:** At first, they should train health professionals to provide a good service.

**P3:** Many people ask us, but no one does anything practical, thank you.

**M: any critical things that you would like to add?**

**P12:** No, thank you.

**M:** Thank you for your active participation!!!

**FGD3: Focus Group Discussion three**

**Moderator:** For transcription purposes, I’ll be recording our conversation, is that OK?

**Discussants:** Yes, it is OK. No problem.

**M:** Thank you for joining us today. We're eager to delve into the intricate workings of Community-Based Health Insurance (CBHI) with your expertise. Firstly, could you shed some light on CBHI enrollment trends and the challenges associated with dropouts from the program? Moving on to the financial aspect, could you elaborate on premium contribution mechanisms and how they're managed within the CBHI framework? We've heard discussions regarding moral hazard, wherein insured individuals may overconsume healthcare due to covered costs; could you outline its impact on CBHI operations? Additionally, how does adverse selection, where high-risk individuals are more inclined to enroll, affect the CBHI pool and its sustainability? Lastly, could you provide insights into specific considerations related to women within the CBHI context? Your expertise in navigating these nuanced aspects would greatly enrich our understanding.

**D9:** Thanks, CBHI has a lot of benefits for those who don't have much money, but it's not in a good picture right now in our kebele. We know that it is useful. I went to the health center in October this year, but when I went there, there were no health professionals in the health center. They told me to wait. I waited until 8:30 local time in the health center. Then a health professional came and asked us why we came. Then, he saw all of us, the CBHI book holder, then he said that if it was not urgent and acute abdominal pain go home and come back tomorrow. Then I asked him to ask him a question. Then I said is this a health center or a court? I said what is the appointment here? I was come for health problem why you appoint me to tomorrow while I am seeking.

After that, without finding any solution, I went to a primary hospital in our district and treated him with my own money. They don't treat those who are CBHI members and those who are paid equally. For CBHI users, medicine is ordered to buy from private pharmacies while drugs are available in health facilities. In general, the problem is not from the government, but at the health center level. Our kebele is on the border between X and Y districts and our catchment health center is very far away. The nearby health center which is located in the Y district is very close to us, so either we go there or we go to private health facilities to get treated.

**M:** Others?

**D8:** The CBHI has been very useful for us, and will be useful for us in the future. As my brother mentioned very well, we are located at the junction of X and Y districts, so we have a far distance to get health service from X district health center even if we are members of CBHI and we can't get medicine even if we go to health center. Because health professionals told us they haven’t drugs.

**M:** Others?

**D6:** When we have become members for the first time, we used the service properly for a certain period as per information from *Kebele*, we were informed that it would help us. However, when we go now, they don't treat us in a proper manner.

**M:** Others?

**D4:** CBHI is very useful for us and it is a hope for our children because we are poor and can’t afford the cost of treatment if we are sick. One day my son was sick and I took him to the health center for treatment. The guardian person told me that the health professionals did not come in and he told me to wait for health professionals. One of the patients was pregnant at the time, so she didn’t get any services because of health professionals’ absence. The other critical problem in our kebele to become members of CBHI is our health center is far away from us and very hard to get service from it.

**M:** How many people are members of CBHI in this kebele?

**D9:** In 2014 E.C., about 25% of people were CBHI members and utilized the service. However, we have a distance issue not becoming full members in CBHI; we told the concerned bodies that we need to change our service obtaining center to Y district regarding CBHI. The government bodies said if you become 50% or more members, you will change your service outlet to Y district.

However, they didn’t keep their word. Since then, many people have gone to Y and Leku town to become members of CBHI, and the number of people who used to be around 60% has decreased to 14%. Now people's hope has also decreased. Only about 17 people have registered this year. On top of this, health professionals in our health center enter to workplace lately around at 4:30 local time and stay only until 9:00. Due to this reason most pregnant women are not giving birth at health facilities. By the rough estimation, only 11% of pregnant women give birth at health facilities, and the rest of pregnant mothers go to other places to give birth.

**M:** Others?

**D7:** The pharmacists are not providing all prescribed drugs for CBHI members or medicines given to CBHI users in decreased numbers. Another thing is that my daughter was sick with her eyes and was treated; they sent her to a private pharmacy for medicine. Those who work in pharmacy have a linkage with private pharmacy. The reason why people don't become members of CBHI is by looking at the availability of the services, so people shouldn't say why they didn't get examined with their own money.

**M:** Does anyone decline their membership because of lack of money?

**D9**: Yes, there is an increase in money to become members of CBHI every time. As you know the life cost is increasing as well as the cost for CBHI is increasing, so several poor people are forced to decline from membership.

**M:** Do you think that the collected money is properly spent on intended activities?

**D8:** No, it won't, we asked the concerned body but the health center professional says that we haven’t received money so you will not get service. I know people who told me to leave the membership of CBHI because the health professional said money didn't come with them and not receive service due to this reason.

**M:** Others?

**D9:** a service provided for CBHI members is not round year even if the membership payment is for a year. Most of the time, in the middle of 4 months the CBHI services will be cut off without using it properly. If we go in October, the service will not be provided at that time. They say please renew it to get the services. When we renew it, the service will start in February or April. We spend our money for a year, but the service is only a short time.

**D6:** My husband was sick, he was very sick and he went to be treated, but when we took my son to be treated, we were told to add more money for the children. Another thing is that one of my sons was sick and when he went to the health center, they gave him the medicine that was given to my husband before, even though the disease was different, there was no laboratory test.

When the children's photos and names were on the book, we were told to bring more money, so we didn't go there again and stopped using it.

**D7:** CBHI beneficiary father and mother, if their child is over 18 years old, they will not be stamped and will not be given services unless they pay extra money.

I am not a CBHI user, but it has been seen in other places that people get a lot of benefits, with us, the number of people who need it has decreased because CBHI users are not treated equally compared to those who are not thirsty. If we are asked for our right to get the services, they say go and file a lawsuit, and we don't have that ability. Therefore, a person has sold his property with his money and started treating it in other hospitals.

**D11:** I signed up for CBHI saying that I am very sick and have no money. Then one day my children took me. Then there was no professional at a health post. After a long time of waiting, one of them came and without examining me, he looked at me and gave me 10 tablets of medicine. Then a nurse said why you don’t treat her. He came and gave me medicine. Since then, I have never been there, so kept my CBHI book at home.

**D12:** I know that I went and got treated, but they gave us medicines, my son was sick and they said there was no medicine, so we bought it from outside.

**M:** Is there anyone who has CBHI members who visit health facilities without getting sick?

**D9:** No, no one goes there without getting sick because the place is far away. We took the mother who was about to give birth to the primary hospital in our district, the way we were going, and she was delivered there.

**M:** Do you know the legal framework or structure of CBHI governance?

**D8:** No

**D10:** No

**M:** Who makes the main decision in the CBHI structure?

**D9:** Is the district manager, they discuss with us, but the problem is from the health center

M: Others?

**D7:** The problem is with the professionals, but the management is good.

**M:** What do you think is the role of CBHI in maternal health service?

**D7:** it is very important.

**D8:** it is very vital for all categories of population.

**M:** Others?

**D9:** it is vital for maternal health services because a pregnant mother needs special support as compared to the general population.

**M:** Anything you suggest to improve CBHI membership and service utilization?

**D8:** First of all, consult the health providers with high-level decision-makers to become ethical and to provide good service for the community.

**D9:** I think several people will become members of CBHI if the health professionals become ethical to serve the community and if we can utilize the service from Y district because it is too near for us.

**D7:** We have discussed our problems with many people before, but there is nothing we can do about it, so let's take action.

**D4:** If there is an improvement in payment, if the road is fixed, I would like it and the community will be motivated to enroll in CBHI.

**D8:** We don't have a health center but only a health post in *Kebele*, so if a health center is built for us, I think that can solve our problem.

**M:** What should the improved from the community side?

**D9:** Community either rich or poor, everyone will be a member of CBHI if the above-mentioned problems are solved.

**D7:** If the problems mentioned by the society are solved, there will be no problem; the society will cooperate in everything

**M:** I have finished from my side, is there anything you would like to raise?

**D8:** There are many people who ask us on different issues, but no one implements our request and that is it, we are grateful for being asked.

**M:** Thank you very much for your time

**FGD4: Focus Group Discussion Four**

**Key: -**

**M**: Moderator

**P: Participant**

Focus group discussion guide for CBHI awareness, perception of affordability of the premium renewals for CBHI, the role of leadership, and quality of healthcare

**M: Could you tell us what you know about CBHI?**

**P 5**: The government planned this scheme for the benefit of poor people in particular and all community members in general. Our community needs healthcare services. We have contributed our share but the CBHI membership card has been delayed for a long time. In this delay period, many people couldn’t benefit from the services, even some died of untreated illness because lack of money. For this reason, many members of the community have decided not to be engaged.

**M: how about CBHI enrolment?**

**P 3**: We are engaged in CBHI membership. But we are not beneficiaries as expected before. We don’t get healthcare services except for blood pressure measurements. No medicines at health facilities. We visit health facilities taking our card, but no services at all. This is a pushing factor for the members. We believe that there is no justice in this regard. Moreover, we are using private health facilities.

**M: Is there a Dropout?**

**P 1**: we the members are proposing to leave the membership because we are dissatisfied with the services given.

**M: what about Premium contribution and its management?**

P 5: But the implementation after the membership and contribution is not attractive.

**M: Moral hazard (the tendency of insured individuals to overconsume healthcare services because the costs are covered by the CBHI insurance) How does this affect the CBHI?**

**P 5**: I took my wife to get medicines for her sickness. One of the workers asked, “Do you have money at hand? If no money, no service with a CBHI card. So, go to private clinics and get treated there”. Look! Now we have no sense to stay in membership. No one has the intention to utilize it excessively. Where is the service first to utilize?

**M: Adverse selection (high-risk individuals being more likely to enroll, leading to higher costs and premiums for the CBHI pool) How does this affect the CBHI?**

P 4: Awareness was created among the community on the scheme. Service absence is one of the factors for engagement.

There is a high-risk group selection approach, but nobody got their card at the time. Poor and high-risk group engagement doesn’t affect CBHI

**M: How about specifics related to women?**

**P 6**: We don’t get services without payment as a member.

**P 7**: No medications there

**M: How does the community perceive the benefits of community-based health insurance?**

P 5: We believe the CBHI initiative is good.

**P 1**: I am a diabetic patient; at the same time, I am a member of CBHI. I need anti-diabetic medicines, but no drugs at all. They refer me with prescription paper to private pharmacies. The cost is scary. We have a huge problem in this case. Look this is prescribed anti-diabetic medicine which is not found in our hospital. Diabetes is a killer and dangerous disease but I have been living for more than a week without medicine. I recommend that the Government control the implementation process.

**P 4:** Health workers of the facility steal medicines from the store and transfer them to private drug vendors. Anti-malarial like Quantum is being sold at private pharmacies. At our hospital, there are many advanced medical machines, but they are not functional.

**M: What is the community’s perception of CBHI benefits, specifically concerning women?**

**P 6**: The scheme could be very important if it served as planned. But no service that can satisfy members

**M: What are the factors that you think can facilitate household enrolment and continued membership in community-based health insurance?**

**P 3**: Service should be available like medicines and investigations. There should be a controlling and monitoring body assigned by the government.

**M: What about among women of childbearing age?**

**M: What are the barriers to enrolment in community-based health insurance?**

**P 5:** Health facilities prioritize non-members as they pay in cash for the services given. They prescribe drugs out of stock or don’t volunteer to deliver to CBHI members without cash payment. The hospital also refers us to buy medicines from private vendors.

**P 9**: I have a chronic illness. So, I visited the health facility regularly and dispensers said “You are receiving it many times, why you don’t buy it once? Why do you need treatment always?”. This is shocking for me. What should I do? I am trying to control my illness as per the physician’s order. How should I manage this doubled problem of disease and professional complaints? Should I die of illness? Even sometimes there is no anti-pain. There are no investigations except the building of the facility. There are many problems.

**M: What about among women of childbearing age?**

**P 11:** We think there is no service delivery for both women and men. All have the same problem with getting medicines.

**M: What strategies could be implemented to increase enrolment in community-based health insurance? Are there any strategies specific to women?**

**P 4**: I think the government should assign responsible and accountable persons from health facilities to the regional level. There should be a control mechanism and continuous follow-up.

P: I believe service improvement is very important to attract new engagement.

**M: Can you describe the organizational structure of the CBHI in your community?**

**P 2**: The structure is not good. The system in health facilities lacks good governance. Hence the structure is not working well. The structure is not clear and ineffective.

Workers take medicines for themselves prescribing as if CBHI members received from the pharmacy. But CBHI members are suffering from a lack of drugs. I think this scheme paved the way to steal medical supplies from health facilities.

**P 9**: We couldn’t get any responsible person to reflect our complaints.

**M: How are decisions made within this structure? Who holds the most influence or power?**

**P 2**: There implementation problem. We are unclear on how private pharmacies can avail medicines while the government can’t access to its people. This is difficult for us to understand.

**P 4**: Kebele manager and health extension workers are facilitating the CBHI process. Health extension workers have a greater role in leading the scheme. But health extension workers couldn’t successfully exercise their responsibilities.

**M: How does the hierarchy within the CBHI affect its operations and effectiveness?**

P 1: At the *kebele* level they are working, but there is a huge problem at health facilities.

**P 4**: I have five family members registered on our household’s card. One day I gave it to the Hospital’s pharmacy professional. That person has separately registered the listed information of my children in his notebook. I think he has planned to take medicines in my children's name. We don’t trust them.

**M: Are there any challenges or benefits associated with this hierarchy?**

**P 4**: There is no responsible person to manage the scheme at the hospital and health center.

**M: What are your thoughts on the leadership of the CBHI?**

**P 11**: The government should assign a responsible and accountable person who leads CBHI-specific activities.

P 10: The leader of the CBHI scheme should not be among health professionals.

P 5: We don’t have any person to claim a grievance. There is no clear leadership and no person responsible and accountable for the management of the scheme.

**M: How does the leadership impact the functioning and success of the CBHI?**

P 7: No positive impact we saw. We are experiencing the challenges only. There is no known leadership to say something about the importance. We are facing service absence and we are not satisfied.

**M: Is there anything would you like to add?**

No one responded.

**Thank you for your active participation!!!**

**KKI1: Key informant interview 1**

**Interviewer: Hello good morning,**

R: Good morning

**Interviewer: For transcription purposes, I’ll be recording our conversation, is that OK?**

R: Yes, it is OK. No problem.

**I: I am a research assistant from Hawassa University. This research is about CBHI and we would love to ask you some questions. Thank you for agreeing to sit with me.**

R: Okay thank you.

**I: what can you tell me about CBHI?**

R: We have been engaging with woreda leaders and kebele, and raising awareness within the community through our visits. Four people visited each of the nine villages we have. As a result, the community is aware of its concept, application, and significance. Most communities in the woreda are members of CBHI. Due to our performance in 2014, we were ranked first. The payment increased to 600 Birr this year, and some people don't seem to be able to afford it. Regardless there are many users

**I: what about dropouts?**

R: Not much, aside from people who are unable to pay for it. Since it would have been extremely difficult to pay for the medical expenses without the CBHI. Here in our community, they educate each other about its significance. About 190 people receive financial assistance because they are unable to pay for the CBHI. All they would have to do is bring their photos, and the service would be free. From my experience, my brother is one of those who is advantageous from this program. He got sick recently and the only payment I made is for transportation. The rest was covered by the insurance.

**I: How long have you been using CBHI?**

R: It’s been four years.

**I: what are your thoughts about the payment system?**

R: When the money collected from members increases to a certain amount it will be transferred to the bank account that is set for this. And payment will be made accordingly

**I: have you ever noticed unfair use of the CBHI membership?**

R: No, we give awareness with the support of the leader. S there is no problem regarding that.

**I: Which group of the community uses it most?**

R: Rich people can pay whatever is asked for the service, but those who cannot afford it are content because they can receive care whenever they need it. Some people learned their lesson from selling their land and going into debt to pay for medical bills. Women, those who have inadequate incomes use it most in general.

**I: How does CBHI cater to the healthcare needs of women in your community?**

R: since women are incapable of going here and there for their incomes, they are happy to use the CBHI.

**I: What do you think are the reasons for non-enrollment?**

R: The inability to pay is the only explanation. This year, the payment was increased and they only had to pay it all at once, unlike previous years when it was small and was paid in three installments. As a result, the people who are not enrolled are those who cannot afford to pay it.

**I: What shall be done to increase the number of enrollments?**

R: Increasing the payment rounds, such as up to ¾ times annually. In this area, Meskerem is where most people get their money, so it could have been better if the payment had been made this month.

**I: What shall be done to increase the membership of women and mothers in CBHI?**

R: Although awareness trainings were initially provided, I believe they would have been beneficial if they had continued. They are informed at conferences and meetings that they need to set aside money for the CBHI in addition to their regular expenses for clothing, food, and other necessities.

**I: How are CBHI organization, decision-making, and memberships?**

R: The mandate includes Kebeles and the health centers. The leader attends training sessions and is summoned to meetings at the health centers. Since there are four of them for every village as I previously stated, they will educate the community about health issues. They advise them to set aside money from other expenses to save for their CBHI. The prompt payment of registered members will be emphasized.

**I: I have finished from my side, is there anything you would like to raise?**

R: I would like to propose that payment options be included, such as the addition of payment rounds and appropriate payment durations.

**I: Okay, thank you very much for your time**

R: Thank you.

**KII2: Key informant interview 2**

**I: Hello good morning,**

R: Good morning

**I: I am a research assistant from Hawassa University. This research is regarding CBHI and I would love to ask you some questions. Thank you for agreeing to sit with me.**

R: Okay thank you.

**I: Considering you are the leader of this health bureau; we anticipate hearing more details on CBHI from you. To get things started, what can you tell me about CBHI?**

R: Here in our city, CBHI was launched as a national pilot site. Afterward, it was broadened before being rolled out across the nation. Those in our society who are incapable of paying for healthcare receive government aid, while those who can pay the one-year down payment can acquire healthcare coverage. We observed several advantages. At First, health services would not be provided to someone who became ill and had no means to pay then. However, if a family member - including children - gets sick after the CBHI, they can visit the health facility and receive treatment by simply showing their membership ID.

**I: How many CBHI users are there in this Woreda?**

R: Since awareness is increasing each year, the performance differs every year. Currently, 21,000 households (71% of the residents) in this woreda are using the CBHI service. Those without stable incomes, such as farmers and merchants, make up the majority of the users

**I: okay… what is the dropout rate and what are the reasons for that?**

R: Some stop participating for various reasons after registering. When people stopped being members, they had complaints about the services the health facility provides and the availability of medications. Due to the current economic state, some members have been unable to pay the slightly increased charge. Even yet, there aren't many discontinuations in our woreda. Since both the service and awareness have been rising, it is less than <3% this year.

**I: what are the complaints regarding the health service**

R: One of the issues is the lack of medications and diagnostic instruments. When patients are dissatisfied with the services being provided, these issues will be encountered. Another issue is dissatisfaction with the healthcare personnel's approach to care. These are the issues we have identified.

**I: What are your thoughts on the premium payment process and its management?**

R: Last year, the fee for newly enrolled members was 450 Birr, while it was 400 Birr for renewing members. However, this year the fee has increased to 600 Birr for existing members and 650 Birr for new members. The amount varies between different locations. It is actually lower in our Woreda. The payment is collected from the months of October to November.

**I: How is payment made to the health facilities?**

R: The health centers provide department-based services by signing an agreement with us. Members use their IDs with their information to access the services. Every three months, health facilities request payment, stating the number of people who received the service. Upon receiving the request, 70% of the payment is made immediately. The remaining 25% is paid after an audit is conducted to confirm that the people indeed received the service, including lab investigations and medications.

**I: Since you mentioned payment-related issues are being raised? What are the questions?**

R: Yes, those who don't have a stable income are the ones included in this CBHI. This includes people like farmers and those who perform various types of work without consistent income. Because these individuals don't have a steady income, they struggle when harvests are low and money is tight; as a result, they tend to discontinue paying for this.

**I: Have you noticed any specific groups or individuals with higher enrollment rates?**

R: The majority of our identified users have chronic illnesses such as hypertension and diabetes. They require regular follow-ups and monthly medication prescriptions, making them a valuable demographic. Even during annual renewals, these patients are the most diligent in reminding us. They deeply understand the importance of CBHI and are our most active users.

**I: How does this impact the CBHI pool?**

R: Yes, chronic illness has a significant impact. The yearly visit rate for People with chronic illnesses is 17. Consequently, the cost of their care comprises a significant portion of the total expenditure. The annual cost for the care of a single person with a chronic illness can amount to 70,000 to 80,000 birr. These are the challenges we face with the Community-Based Health Insurance (CBHI) program.

**I: okay my other question is, do the users/ members use the CBHI appropriately?**

R: When it comes to health insurance awareness, it's important to understand that not all members will necessarily become ill and utilize the benefits. Contributions are pooled together, and those members who do face health challenges will be the ones to benefit. However, some members complain that despite paying 600 birr, they have remained healthy and haven't utilized the insurance. As a result, they tend to visit health centers for minor issues or even when they feel completely well, demanding medications. They think that their insurance will expire if they don’t use it. This behavior poses another challenge for the system.

**I: How does CBHI cater to the healthcare needs of women in your community?**

R: When it comes to women's health, it's important to acknowledge that women are more biologically susceptible to getting sick. The CBHI program allows women to easily access health services when they encounter any health problems. In the past, people would resort to extreme measures like selling their livestock or getting into debt to pay for healthcare when they or their family members got sick. However, with the CBHI program in place, urgent health services are readily available whenever someone falls ill. Nowadays, when a mother faces a health issue, all she needs to do is present her CBHI card at the health center to receive the necessary care, without having to worry about finding money for healthcare. This program has been widely embraced by mothers and has significantly improved access to healthcare for them.

**I: you have raised that the majority of the people use CBHI, how does the community perceive the benefits of CBHI?**

R: Their awareness is getting better day by day. Based on our woredas's performance, in 2012 our coverage was 2.5%. In 2013, it was 27%. In 2014, it was 50.4%. And this year, in 2016, it reached 71%. This shows that people's awareness of CBHI is improving and becoming clearer over time despite the challenges we faced before. The issues they raise are that with the money they paid, others get treated and they haven't gotten sick the whole year, so they feel they didn't get their money's worth. But we think that the situation is getting better with time.

**I: okay in 2014-2016 it clearly shows a growth in CBHI use. So, what was done for this?**

R: First, we tried to raise awareness in the community through various platforms such as social media, local advocacy, meetings in neighborhoods, and other channels. This was aimed at informing people about the services available and encouraging them to share their experiences. We also conducted awareness campaigns in churches and other religious places, with the participation of political leaders. We used these different options to increase participation.

**I: what do you think should be done further to increase the number of users or decrease the dropouts?**

R: First, it is important to focus on the preparedness of health centers, especially regarding medical supplies. The second aspect is the opinion of healthcare professionals on this matter. Improving these areas would benefit the community and increase healthcare coverage. The primary focus for the government should be on preparing health institutions for potential challenges.

**I: you’ve mentioned that the amount they pay is one of the reasons for discontinuations, what do you think should be done?**

R: For those who cannot afford to pay, the government should allocate enough budget. Cutting down the payment is not an option, as the payment increases due to medication expenses and medical supplies. The cost might increase to 800 to 1000. Therefore, the government should allocate a budget for those identified as the poorest of the poor.

**I: regarding women’s health what setbacks are there for women not to use CBHI?**

R: we haven’t encountered that many problems. We haven’t identified any issues regarding women using CBHI.

**I: okay we’ve talked about reasons for withdrawal, what issues are there for others who haven’t enrolled yet? what do you think should be done to solve these?**

R: The first issue is awareness problems. Some people tend to associate it with religion, stating "Thinking that I am going to be sick is like planning for myself to get sick. God will protect me." Some groups believe that health insurance is useless. To address these issues, we are collaborating with religious institutions and engaging in discussions with religious leaders. We have organized meetings with religious leaders in local communities to encourage them to incorporate our message into their teachings. Additionally, we are encouraging individuals to make monetary contributions at religious places and to donate on behalf of those who are unable to afford it.

**I: can you tell us about the hierarchy of the CBHI**

R: The office states have a defined hierarchy led by a group of leaders. However, there are nationwide hierarchical issues as the CBHI should be independent. In this current system, the health sector buys and sells health services. To address this, the CBHI should become its institution and manage itself. From the federal level to woredas, there are concerns about the lack of organization. Health centers lack focal persons and users do not receive necessary assistance because of this. Despite these hierarchical issues, the CBHI currently operates under the control of the health bureau.

At the federal level, CBHI is an autonomous organization reporting to the Ministry of Health, while at a regional level and levels below that it is not a standalone organization but administered under the health offices. From regions to woredas, there are clear hierarchical issues. There have been raised laws in parliament regarding hierarchical arrangements that were not executed.

**I: what problems did you face given there are hierarchical problems?**

R: The problem we face is that the health sector both sells and buys health services. If there were an institution that sold health services, the health sector would have been better prepared to handle it. Agreements with the health facilities are made without a clear understanding of the available services and the price of the services. The facilities are not adequately prepared in terms of stocking medications, lab equipment, reagents, and the preparedness of health workers. If there were an institution involved, there could have been an agreement signed between the two based on the services and price, resulting in better services for the community. Currently, the agreements are signed without common understanding and member complaints (CBHI users) face problems due to these.

**I: how do you reach the community after the woreda level?**

R: Members are recruited by us, and we receive support from other stakeholders such as political and religious leaders. The health sector has direct contact with the community. We recruit members, collect money, and issue IDs.

**I: how do you think leadership impacts CBHI?**

R: The performance of the leader is reviewed by a sting committee to identify obstacles and other issues. The committee is led by the Woreda administration, with the office administrator serving as the vice chair. We review the challenges and provide solutions for them. Performance is also assessed on a daily basis. The woreda administrator and regional leaders get to decide on issues being raised.

**I: I have finished from my side, is there anything you would like to raise?**

R: No, thanks.

**I: Thank you very much for your time**

**KK3: Key informant interview 3**

**Key**I: Interviewer

R: Respondent

I: Please can you tell me a few things about yourself and your current position?

I: My name is XX, eh…my position is head of agency at X cluster. Eh… I have been working at this office since the establishment of health insurance, now it is around 10 years. Formerly, the office was called a health insurance agency, however, after three years onwards it is called a health insurance service.

I: Would you please tell me your views about CBHI, before we move on to the main questions?

R: Eh… when we say CBHI, eh…it encompasses non-regular income groups/economic classes of the community; eh… those who do not have regular income from government or company in the form of salary or pension. Eh…farmers, livestock farming, eh…those people create their income in urban settings. Eh…therefore, in my view, CBHI, though the community did not well understand it as it is the newest, is a means you expend a small amount of money and get treatment for your whole family. Eh…CBHI solves major health problems of the community that the community visits the health facility and acquires health care services. Eh…the people, specifically, farmers do not have regular income that they have at some period while they do not have income at another time. Eh…farmers get the money during harvesting season; whereas, they lack money after that period eh…they are unable to get appropriate healthcare services at that time eh…interrupting treatment, using traditional drugs eh…and enter into the poverty stage. Eh… or else, the community may fall into a state of begging. Eh...CBHI usually limits the community’s suffering from death due to the absence of healthcare services. CBHI has great benefits for the community by preventing severe disease and death.

I: What do you mean by the community not well understood CBHI?

R: Eh… most of the time, people learn things from experiences eh…educated persons learn from international point of view that they can better learn by reading, hearing or observation, however, at farmers’ levels, in most of the cases, things being understood by observing practically or liven experience. Eh…people do not save money for the sake of his/her own health but also those who are educated are not doing so, eh…we face with problems when we are experiencing unhealthy condition, even those who have income.

Therefore, the illness will not come with plan and we could not estimate the expense for it and difficult to guess the amount of money needed. Therefore, planning ahead, before one gets sick that he/she could face illness requires awareness. Eh… therefore, creating awareness is mandatory and the solution for this is saving money when they have on their hand and being fully insured for their own health is needed. Eh… for example, in insurance people buy it for the safety of their cars or equipment not to gain money for it, eh…similar to this people do not think in the same way for their own health insurance that their lack of health insurance does not create fear in them and this needs awareness creation in our country. In developed worlds, health insurance is a big issue; however, in our setup it requires exposure and learning from one another, eh…people are getting more experience on CBHI than what they hear from the media.

I: How does the community view CBHI benefits?

R: Eh…CBHI issue is positively progressing. Eh… during the establishment period, people even refused to accept the idea of CBHI that they should not plan about their future health condition and give their health issues to GOD and have hope in GOD. Yaw…the community started to believe that people can get sick by the will of GOD and GOD’s person could get sick. The CBHI idea has progressed a lot in the last 10 years and now it is in good condition and people are well aware of it.

I: What are the reasons for well understanding of CBHI by the community?

R: Yaw…there are two to three reasons for this, first, we use different media including social media and public media as well such as FM radio, TV, and regular programs especially, when the windows for health insurance open in two months or based on income from the local community context and we use different alternative media accordingly. Another is we use mentors, gatherings eh… with the help of banners & pamphlets at various conferences. Another issue is eh…informal discussion made among people who got the CBHI services in the area, eh…they ask each other how they received the health services, eh…improvement from illnesses that those who benefited from CBHI share experiences.

I: What are the benefits of CBHI for mothers/women?

R: Eh… from the mothers' perspectives, eh…CBHI is bought for the sake of all household members and benefits all family members in that there are no differences for males and females; however, benefits it has for females are in this area, and in Ethiopia in general women wait for their husband’s response to get healthcare services, this is completely changed in the era of CBHI that any women having CBHI ID card can visit health facility without the will of anybody. Eh…even when there is no money on their hand or their husband is not able to pay for health care or else, when the decision to seek health services expected from their husband, for example, in rural areas a husband may have two to three wives, but no need of support of their husband to visit health facility because of CBHI.

I: Do you think CBHI empowered women than others?

R: Yes, CBHI increases health health-seeking behavior of mothers. Eh…though no studied evidence on my hand, I expect that the health-seeking level will increase for everybody, for example, those who do not have money for health care, for a card, etc. will easily use the services. Above all, mothers’ interest in visiting health facilities increased a lot.

I: What do you think created favorable conditions for households to enroll in to CBHI program?

R: Eh…first of all, health insurance is simple on its own, for example, any member of the kebele who has an ID card or even if he/she has no ID card and the officials have trust in him can enroll into the program. It is convenient for all community members as there is no complex criterion for joining CBHI. Eh… no extraordinary opportunities created by CBHI in accessing health facilities; it is the health sector that provided them with this chance. Eh…another is a single card works for all health care services and no requirement for a card every time visiting the health facility. This is a good opportunity though it has some drawbacks, for instance, one person may misplace or forget a pocket, and other members of the family will suffer.

I: Please, do you mention any reason for facilitating women’s CBHI membership?

R: Eh… as said already there is no distinct reason for facilitating women’s enrollment in the CBHI program, however, in rural areas, there might be widowed women, eh…in the lowest poverty line even those could not able to pay CBHI contribution payments, eh…those below poverty line and their contribution covered by public expenditure, as some study shows formerly nearly 10% were unable to pay, however, at a moment 23% of the community could not pay CBHI membership fee. Hence, eh…widowed women in rural areas are prone to falling into the poverty line because agriculture requires strong labor which is not the case for women in rural areas and could not able to work hard in agriculture. Eh… some women are not able to pay for CBHI but the government supports them and there is much work to include them in the CBHI program.

I: are most of those poorer of the poor people supported by CBHI women?

R: eh… nonetheless we did not conduct a study, those people enrolled in CBHI as poorer of the poor would more likely be women eh…because they might live with their husbands or if they are widowed, they will be included as poorer of the poor.

I: It is believed that those with chronic illness or serious health problems are more likely to enroll in CBHI. What do you think on this point?

R: Eh…yaw, this issue is not only on women, but they are the first to come for the services. Eh…people with chronic illnesses can calculate their yearly expenses for drugs and treatments and they are faster in deciding for CBHI enrollment. Therefore, they are the first to join CBHI when they hear the start of the community-based health insurance program. Eh…you cannot prevent them from joining by observing their health status as this is not for profit program established at the public level and it is their right to join CBHI. Therefore, to prevent such a problem we set criteria to establish a CBHI in a given community that at least 50% of those eligible individuals should join it.

This is to sustain the program in the given community because; including only a few people will affect the financial future of the program. Eh…therefore, more than 50% of the people should be enrolled in the CBHI to minimize the problem because not everybody included in the program may get sick. Similar to vehicle insurance that there are cars not will be damaged at the end of the year while those damaged will be paid from the contribution of others. Likewise, CBHI expects that not everybody in the program gets sick and those people with illness will be paid from the contribution of healthy ones. Those with chronic illness will be benefited more than others as about 20, 000-30,000 birr may be required to cover their expense.

I: you rightly said the CBHI contribution is collected during some seasons considering income; however, the government may collect tax and other things, three or four things at one time. Is there such an issue in your area?

R: Eh…yaw, tax and health insurance and all bodies collect money during better income period in the community because it creates favorable conditions for their work. Eh…those who collect the money are administrators in the peripheral levels and this is done during better income season and could be similar for types of revenue collection. Eh…there is tax revenue, different expenses for developments, eh…regular sports, etc. will be asked at the same time and this will produce unfavorable conditions for CBHI contributions. Eh…sometimes payment for the security of the country, eh…another time for supporting famine-affected communities in other parts of the country and likewise community expenditure increases and this hinders their payment for CBHI, and sometimes we encounter the complaint. However, health insurance got a higher priority in regions like Amhara and some parts of Oromia and people refused to pay for other purposes before CBHI. Therefore, CBHI is well understood in some areas than others. Eh…asking for all payments at the same time creates a problem.

I: What prevents people from enrolling in CBHI, as you have said already some associate the illness with the will of GOD. What other challenges?

R: eh…there are barriers; one of these eh…understands problems. However, the major barrier is the health services delivery issue at the health facilities which is not at expected levels. In another area, there is a working system called pocket replacement, for example, eh… as an experience though not helpful in Amhara region, individuals not getting required services in the public facilities could able to receive health services in private sectors and brings receipt and will be compensated for their expenses.

Finally, they are slowly avoiding it and looking for another mechanism due to wrongful utilization of the system. However, in our area, there is no such system and there is a dropout from CBHI as some people in one year may not continue in another year. This is because services at the health facilities are not complete, for example, they may not get some services or some drugs and are told to buy from private facilities though they have health insurance licenses and expect that their health expenditure would be covered by public health facilities, and may not have money on their hand. Eh…sometimes people talk about minor services received from outside the public health facilities though a majority of the health care services given to them. Eh…they do not estimate the amount of money expended on them at public health facilities, but rather, they bother about the money expended from their pocket.

For example, a client who received 500 birr for services at a public health facility with CBHI and also expended 100 birr outside to buy some drugs, /boldly talked at coffee and other places about this issue eh…either deliberately or unknowingly as he may not know how much registered there. Eh…indeed no member of CBHI should spend from his/her pocket for health care services and this is one of the obstacles. Eh… secondly, kebele leaders may not ask for CBHI contribution expenses in a timely eh…not giving proper services, not providing kebele ID cards, or not giving CBHI receipts in a timely. Eh… for example, for new members, they give new ID cards and for permanent residents, they renew the ID card. Eh… sometimes they collect money for CBHI from the community but they delay providing receipts to the individuals or else the people themselves delay bringing the receipt to the insurance agency. For example, they collect the money however, not deposit it in the bank, and the renewal is performed at the woreda level and individuals delay the services as their money fails to be deposited to the bank.

I: what challenges in facilitating CBHI from the leadership, or services from the agency point of view? In general, what challenges are there regarding CBHI implementation?

R: Eh…regarding the CBHI some enrollment issues are not complete; eh…some establishments are complete. Eh… there is no current law governing CBHI except the 2014 proclamation, therefore, regions are passing some governing rules by themselves. Eh…there is a national proclamation passed during 2014 that contains administrative structures and eh…there is a national council at the national level that meets once yearly to hear on the health insurance report and passes strategic agendas. Then, there is a regional council at the regional level meeting twice a year and passing decisions based on the national council agendas. Eh…then, at the Health Bureau levels, there is a CBHI office that has its structure and leadership. Eh…there is a CBHI council at the zonal and woreda levels and at woreda, the council has two members from each Kebeles. Ehh… at the lower levels, there is a CBHI scheme consisting of three members, a health professional, an accountant, and IT personnel. Eh…they usually report on the CBHI status of the members and financial report quarterly, bi-annually, or annually.

All the financial collection, members’ registration, and giving orders are being done at the woreda level, eh…our office role is facilitating the process by providing favorable conditions, giving training, eh…making supportive supervision. Eh…reporting and auditing our activities with the main office at the end of the year. Eh…challenges from our side might be weakness in mobilization and awareness-creating activities. Eh…we transfer all the money collected through CBHI to the health facilities at the woreda level and we do not have a role in service delivery.

I: as explained earlier about the structure and flow of activities of CBHIs in detail, how much do you think this method is effective? Eh…in making decisions?

R: the CBHI structural functionality is incomplete, for example, there is no CBHI council at regional levels because the law itself was approved in 2014 (2022), however, we would have moved faster.

I: What reasons can you mention for the delay in the regional council establishment?

R: eh…the delay comes as a result of the length of the procedures that the national council itself began functionality last year and the prime ministry is the leader of the council. Eh…no recent council meeting was held. However, we are expecting a meeting of the council shortly, then the regional council will be established. Eh…we are waiting for the higher officials’ response and the health bureau is responsible for facilitating this issue. Eh… not much problem because of the regional council's absence as the previously formed structure is working very well, for example, the woreda councils are working even though there are some interruptions. However, the woreda CBHI scheme is working properly, but the regional council presence will strengthen the system very well.

I: How are the CBHI services established in the health sector?

R: it is just like the other departments in the health system, for example, the Disease Prevention and Control department, the CBHI department, or unit forms in the health offices.

I: what did the previous trend of CBHI form look like?

R: until this day CBHI functions as a desk

I: What is your response on the CBHI members’ frequency of health facility utilization, for example, coming repeatedly, visiting with minor or without illness?

R: Eh…such things usually happen due to a shortage of awareness, despite reducing at this time. It is not a big issue at the moment. Eh…the HF standards were not changed and they have their working standards to provide health care services to patients, they know how much it takes for a patient to improve from illness. Eh…sometimes, some patients expect improvements immediately even in the absence of CBHI, and they come back the next day complaining of illnesses without completing prescribed drugs for a week.

However, what makes the current one different is, that formerly patients have patience for the illness for fear that they will spend a lot, but at this time they do not expect expenses for the health care treatment services as they have CBHI membership ID. However, the health care provider (HCP) at the health facility follows his/her standard of care through the patients visiting for minor illnesses. Eh…another point is there is an awareness creation session at the health facility in the morning that some CBHI members should not visit repeatedly. Eh…there is a trend of pretending to have coughing and treated and giving the drugs to his/her neighbors, so he/she is affecting his/her association. There are a few such problems in the community though not common.

I: what opinion do you have on the lower level of CBHI leaderships or focal persons?

R: Eh…persons supporting or leading CBHI, more commonly woreda administrators are usually working on CBHI, they provide support and leadership activities up the party level. Eh…secondly, kebele leaders, and thirdly, health extension workers by mobilizing the community towards CBHI. Eh…in some areas employed kebele administrators are working on CBHI. Eh…these bodies have their demerits, eh…at kebele level delay collected money deposition to the bank as they usually give priority to other competing problems. Eh…at the woreda level, despite limited access to collected money for CBHI, there is a tendency to delay CBHI program implementation for the fact that some are not well informed or they may not have benefits of CBHI as there is high shifting of position.

Eh…affecting the CBHI as they do not conduct the work properly or do the work inappropriately, delaying the CBHI money, eh…using the money for their private issues, etc. We, most of the time, provide supportive supervision and monitoring on how much money they have collected, what numbers of CBHI members registered, and what they did with the collected money at the woreda levels to address the problem. Eh…we arrange some review meetings and make evaluations with the head office; eh…woredas with poor CBHI practices will be identified and inquired for their failure that is the role of our office.

I: What impact this leadership flapping will have on the CBHI's effectiveness?

R: This will have an influence/effect on the CBHI effectiveness, in addition to what I mentioned so far on the incompleteness of the services at health facilities, eh…sometimes people delay in getting health care services even if they paid for the CBHI memberships that rather than visiting HF when their children get sick, they blame the leadership. Because of these issues number of CBHI memberships become stagnant, for example, in the Sidama Region only 60% of those eligible joined the association.

Hence, 30% of eligible individuals failed to join the CBHI. Eh…not only were those without knowledge of CBHI who are not joining it but also those members dropped out of the memberships. for example, there was a 19% dropout rate in the last two to three years in this area. The reasons could be these people were not satisfied with the health insurance services and decided to stay away from it. Eh…we had expected 100% joining of CBHI, however, individuals might not be interested in the services at the health facilities due to less availability of required services or the way health care workers approach patients, at the woreda/kebele administration, and so on.

I: What is/are the main reason for the failure of CBHI enrollment, you stated that there was a 19% dropout rate.

R: The main reason is problems at the health facilities, eh…healthcare workers unfriendly approach and lack of drugs at the HFs. Eh… the shortage of drugs has been common for the last two to three years as the dollar exchange problems and some factors in the country stopped production of drugs. Eh…drugs imported from abroad may not arrive on time. Eh…there is an occasion when the government has a budget shortage or lack of drugs in the presence of an adequate budget.

I: as per the complaints from the health facilities as a major challenge for the dropout of members of CBHI, there is a delay in delivering CBHI income to the facilities until the last quarter. The HFs use this money to replace drugs as they should be recycled. What do you say on this issue?

R: By the way, the problem is less significant in our city, however, at woreda levels the premium, money collected from individual members, is inadequate because it is a trial so that people will join more. This is for the fact that in rural settings people usually have low incomes so they may not be able to pay in case more money is requested. Last year, the membership fee was 400 birr, but this year increased to 600 birr. Eh…the lowest payment by members is the Sidama Region, which is 600 birr, the rest regions collect a minimum of 800 birr, 900-1100 birr even more than this per year for the memberships.

Therefore, the main problem in this area is an adverse selection of members so that they are the first to utilize the HF services and to renew their memberships. Eh… when we close the budget of tir/January it will be empty throughout and may not refill until the end of the year to pay for the HFs. Eh…there is a lack of trust between woreda and HFs in some areas on the amount of money requested saying it is too much money. However, in most cases, the woreda administrators withhold or delay money when there is a minimal amount in their bank account. This is not good practice. As a solution increasing the premium amount is necessary from time to time. Another point is that HFs have their problems such as asking for extra money, eh…ordering more than affordable levels so that members are forced to buy from outside the HFs; eh…there is a tendency to register exempted services already paid for by government; eh…HFs want to increase their income as well. In general, collected money could not be able to cover the whole year's health care insurance fee. Eh…HFs and health offices need to be monitored as they buy and deliver services. I mean they buy drugs themselves and provide health care services as well; however, the woreda has no role in buying drugs. Eh…we are trying to close gaps in reducing corruption among buyers and sellers. In the future, there will be a separate body facilitating buyers and sellers as a regulatory body to negotiate between these two sectors.

I: What to be done to improve CBHI enrollment, in addition to what is stated above, things that could bring change in enrollments?

R: Eh… the major issue is improving services at the HFs. Eh…correcting managerial issues at the HFs, sustaining drug supply, eh…should be functional in all aspects. For example, if someone comes to us complaining about HF or the absence of the services, there should be evidence to demonstrate to the person that the services are there and we should improve our HF standards up to this level.

I: Who has more power in deciding the CBHI?

R: Eh…more power is in the hands of Woreda administrators as they have full power to collect and increase the amount of money, eh…if the account gets low, he can increase by collecting from the community or transferring from another source. Hence, it is the woreda leader who can increase the budget and sustain the program. Eh…the woreda head has everything on his hand because, he holds the account, and eh…he is board council; eh…mobilizing the public to contribute to the CBHI and giving priority for collecting CBHI contribution from the people; eh…he can control HFs, eh…monitoring misuse of drugs at the HFs, eh…currently he has the power to control overall activities regarding CBHI.

I: Does Woreda apply another strategy rather than what your office recommends?

R: Yes, he can. Eh…yes for example, he can decide on the budget in October or November, eh…there was support for the poor of the poor people before the CBHI, and were treated freely by giving yellow cards, and the woreda paid for what they have used. Currently, this mechanism has ceased because of CBHI. Similarly, there is a budget for poorer people planned and paid for the HFs called targeted subsidy. This targeted subsidy means the budget being allocated for the poor and their ratio taken into consideration making it sustainable and included in the CBHI. Eh… some leaders take control measures on the health facility leaders or the scheme leaders and change them with appropriate individuals. Therefore, there are woreda leaders who make decisions with their board and take measures on their own than what we recommend.

I: do you remember any strategy designed specifically for women to enroll them in the scheme, or any experience you can share with us?

R: Eh…not yet.

I: do you have anything you think worth mentioning on CBHI?

R: Eh…in general the CBHI in Sidama regional state health facilities should be improved. Eh…we suffer a lot to increase the number of members enrolled in the CBHI every year, and every year there is dismissal of administrators in the process of increasing the CBHI members. Eh…growing members in the region is as difficult as pushing the mountain and is the procedure everybody board with, therefore, this issue should be studied well. Eh…I expect that people come in groups to buy health insurance in the Sidama Region if only challenges are solved. One of these challenges is poor health care services at HFs.

I: Can you explain health facility maltreatment/abuse of patients?

R: Eh…when I say this, I am not saying health care workers, but those who work in the card room and accountants. Eh…patients do not get cards easily as they will provide cards for both non-CBHI members and CBHI members, two cards at a time and there is a trend of no searching for the cards while they provide the new card. Eh… consider CBHI users as help seekers maltreating them and discourage them. Another issue is that in the financial room area, people are naturally interested in touching money and those daily cash collectors expect something from patients and nothing being paid by CBHI members and delays their services or maltreat them.

I: Thank you, it is over

R: Thank you!

**KKI4: Key informant interview 4**

**Key**I: Interviewer

R: Respondent

I: We had asked lower structure on factors affecting the CBHI scheme and we gathered responses as health facility-related, poor hospitality, poor services, shortage of drugs from community financial problems, etc. Now we are more interested at higher levels in discussing CBHI structure and functionality on its effectiveness.

R: Ok…in our level, the CBHI is under RHB organized as one of the health care teams, eh…there is a regional community health structure assigned personnel considering the biography of the regional community. Eh…we have a structure in our office and three experts working on this CBHI case team. Eh…these people are working to fulfill government strategies on CBHI and I do not see other things, however, issues you stated are expected at the community level though there are variations from place to place or from structure to structure, experts of CBHI in this office and agency work together and will solve those issue stated based on the rules and regulations contained in the CBHI guidelines and there are additional support provision. Eh…by additional support I mean financial support to strengthen schemes at various levels, eh…benefiting poor of the poor and the regional government covers 70% of the financial support. Eh…to preserve the standard services at the peripheral levels we fulfill infrastructure at our office.

I: Do you think many manpower in your office is adequate to support CBHI at the lower levels?

R: Eh…study on manpower needed in this region was not taken into consideration the regional structure and is inadequate. For example, we have four zones and one city administration, but manpower assignment would have considered this and the structure and manpower needed at the peripheral level. Eh…seeing from this viewpoint, additional manpower is needed in our region.

I: How much does CBHI structure or hierarchical arrangement from federal to Woreda levels help or hurt CBHI's effectiveness? Operational effectiveness?

R: Eh…I believe that the presence of the CBHI scheme at all levels helped the program. I mention because there was no this structure when we were at the zonal level, eh…when we see the functionality, it was led by a political leader and HB head, and only when the direction given to others would involve in CBHI, and there was about 10-15% achievement rate during Sidama Regional structure formed. Eh…persons assigned in the CBHI scheme plan and create agendas on leaderships and at the current time our achievement is 69-70%.

This means, the presence of structure nearby, created a sense of ownership of the program and has brought this good achievement. Eh…there is a plan for next year's CBHI activities and expectations through self-monitoring and evaluation of the program which is done hierarchically and publishes in Broacher. Therefore, I believe that the presence of the structure of CBHI is very helpful. However, there are gaps in structural functionality and effectiveness. For example, CBHI's structure being holistic in the early identification of alternatives to accomplish its objectives has some weaknesses. Eh…I think some additional work is required to improve this.

I: Please explain the political agenda of CBHI.

R: Eh… at that time there was no adequate and organized manpower in the CBHI system, therefore, it was a political agenda organized under the Woreda administration. Eh…here at the regional level there was no structure in in CBHI scheme looking for experts that support and mobilize it. The achievement was low as a result of the irregular and intermittent meetings of political leaders and no daily follow-up; however, it has become a routine activity at present and they support work and come up with input for evaluation so that any organization that wants to intervene may utilize their reports.

I: Regarding CBHI, would you tell us the sector with more power in decisions on health insurance issues?

R: Eh…the office taking a lion's share of the CBHI program is the health sector, then the political wing means regional to woreda level administrators direct the board members and organize CBHI monitoring and evaluation.

I: Can we say the CBHI activities are as a campaign?

R: Eh… it has some period considering harvesting seasons (agro-ecology), for example, in Sidama Region, the collection period is from October 1-January. Therefore, the society will get money by this period and can contribute to CBHI membership. So, this is not a campaign, but it is an accomplishment period.

I: What view do you have on CBHI structure, from higher to lower-level hierarchies?

R: Eh…the leadership gives more emphasis to CBHI than any other agendas. Because the structure focuses on serving all types of communities without discrimination and enrolling all types of people such as those who can pay, those who will be supported by the scheme, and no one to be spared. Another point is our political party has given due focus to CBHI as we have a vision to create a healthy, developed, and prosperous community. Eh…our communities are reluctant to expand for their health and give less attention to their health status, therefore, CBHI membership and contribution will solve this problem and increase health-seeking behavior so morbidity and mortality will be minimal and productivity will improve. Hence, political views have created health ownership and changed our communities’ health conditions.

I: How does the society view CBHI?

R: We classify this into two. The first groups are those people with chronic diseases such as hypertension who utilize more of the resources as they stay for weeks or months in the hospitals and the referral system is organized up to a higher level, I know a patient who went through all these stages and treated at black lion hospital, these types of people talk positive things about CBHI. In another view, there are members in the community who contributed the money but did not get sick throughout the year who complain about their lack of CBHI utilization ignoring common values and supporting one another so that they fail to pay a renewal fee.

I: Is there any distinct move to enroll women/mothers into CBHI in this area?

R: Eh…women who have household status will be enrolled as other households and there is no separate work to enroll them when they have a husband.

I: Are there barriers preventing women from enrolling in CBHI?

R: Eh…may be a barrier from women's side are their poor educational status in rural areas that they are unable to differentiate prior payment and treatment seeking after illness is not acceptable, and hence, they usually reluctant to join CBHI. The second group of women is those who live separately from their husbands will be considered as a household and others whose husbands shift to other areas may be psychologically and economically affected, and they might not easily decide to join CBHI. Therefore, these women are unable to make decisions and financially incapable of paying.

I: What strategy do you suggest in improving CBHI enrollment rate as a general and particularly among mothers?

R: Eh…as a strategy we should make harmony with the society, which means mounting the benefit of CBHI and that we are improving it to the community. Another eh…is improving facility-level services to increase the enrollment levels. Eh…people will not come back when the service is poor or lacking when the referral is high, and may not accept the CBHI program. Eh…constructing additional HFs and equipping them with human power is mandatory for CBHI enrollment to improve. Eh…we should encourage tradition of supporting each other and the government should allocate adequate budget and timely release for the HFs.

I: What does the budget-releasing mechanism for HFs look like at the moment?

R: Eh…regarding the budget release, it is almost good because they release 75% or more of the total budget, despite not being timely. Eh…making a budget release periodical is a must. Eh…quarterly evaluation and release of budget to health facilities are mandatory, however, auditing and timing have some drawbacks. It is the problem of sluggishness of our structure in making earlier audit and budget release not the problem of financial inadequacy because it is already deposited to the bank. There is a leadership problem in pre-auditing putting direction and working collaboratively with woreda-level managers to do so. We know how much HFs should get, maybe 100K or 200K of maximum payments from woreda and I do not think asking for extra payments from the CBHI scheme is an issue.

I: What is a delay in decision-making by leadership?

R: Eh…not making timely auditing of the financial report by HFs. Eh…auditing is a working mechanism and to be timely audits can filter any problem in financial discrepancies and give feedback to HFs.

I: in your opinion, how best the CBHI be structured to be effective? From the top level, federal to the lower level, the community?

R: structurally, at the moment additional manpower is needed at all hierarchical levels, and more personnel is required at the *kebele* level. There should be a CBHI coordinator at each kebele because, currently, health extension workers are doing this job, and eh…kebele administrative who are doing CBHI activities. To develop a strong structure for CBHI these things are important, especially, establishing a CBHI structure at a kebele level that increases community awareness, then educated people pay for CBHI membership during the off period, a time when CBHI contributions are not collected, other than October to January.

Eh…others will not teach about CBHI, therefore, if the CBHI structure is established at a *kebele* level where individual households contribute to CBHI memberships these personnel will teach the community, eh…teach by taking better users as a good example of CBHI members, eh…always mobilization will be made, always awareness creation activities will be implemented. Hence, I support if manpower is added at the lower, *kebele*, level in the CBHI structure.

I: Would you mind adding points not mentioned so far, regarding leadership, structure, etc.?

R: Eh…I suggest leadership activities should improve early mobilization activities so that many people will benefit from the CBHI scheme. Eh…timely mobilization, and timely awareness creation to embark on actual work of CBHI is recommended. Eh…those at the lower level of the CBHI structure create favorable conditions and reminding them to progress more is important. Another is organizational readiness is very important. Eh…members of CBHI who paid money should get treatment where are living rather than referring for better services. Members not be disappointed, and lose trust in the CBHI program which may delay accomplishing our objective. Therefore, fulfilling inputs and correcting faults has a significant role.

I: Thank you very much for taking the time and reply

R: I also thank you!

**KK5: Key informant interview 5**

**Key**I: Interviewer

R: Respondent

I: The interview was led by XX. Thank you very much for your willingness. Before I move to the main questions, please tell me your age, current position, and the CBHI-related experience you have.

I: My name is XX, age 35, second-degree holder/MPH. I am XX Hospital CEO. I have around 20 years of experience. Eh... regarding CBHI, I worked in the pilot woreda as there were some woredas in the Sidama region selected for a pilot study, Hula woreda, and have more/less experience and skills in CBHI.

I: Could you tell me what you know about CBHI??

R: Eh… when we say CBHI, we mean community-based health insurance. When we see CBHI from its objective side, one…it is designed to equip the society with insured health status. Eh… for example, take societal issues such as *Idir (a traditional savings method for bereavement such as a funeral)*, community contribution for the sorrow of their death, and Ikub *(a traditional savings method to mobilize resources)*, which is for economic values. Eh…Ikub is for trading, and Idir is after your death to serve your guests not for yourself, however, CBHI is for the living person, eh…you or your family members want to visit a health facility to use health services eh…you may not have money to buy some drugs say paracetamol.

Therefore, he will have insurance for his illness which means it differs from those mentioned above. Eh…one point is that society saves what will help get priority in treatment and support. Eh… secondly, it is a matter of supporting one another for example, in a given woreda 10,000 people may live and all could not get sick at the same time, say 400 individuals may contribute money but not all of them will get sick eh…if the cost of treatment for one person is 20,000 you are supporting him with the contribution of healthy people.

So CBHI is to help you before you die, unlike Idir that you save to serve your guests in your earthly absence. Eh…the third and the main objective of CBHI is to increase the potential of the health sector's financial capability as health expenditure…as we all know our health system mostly relies on donors’ money and if this donation gets interrupted then CBHI will solve this problem. Eh…if all people in Ethiopia enrolled in CBHI, think how many billions of birr we could collect. Hence, the health sector will be financially empowered and we can keep this for emergencies. In other words, the health sector will have sustainable financial capacity; eh…the health sector will cover the gaps that could be created by donors’ cut donations with the help of CBHI.

I: Would you please tell me about CBHI enrollment and dropout rate?

R: eh…this is when people enrolled last year fail to continue with the membership. Eh…this has happened before, whereas, by this time the enrollment is improving. Eh…dropout rate means assume that we had ten thousand members last year but only 9,000 of them renewed their license so we have 1,000 dropouts. Whereas enrollment is the new membership being enrolled as a new. Eh…if we see factors for previous dropout problems, for example, lack of awareness about CBHI could be one factor. Some (enrolling individuals) even educated them as if membership is once and health service is forever {laughing}…that is problematic.

The second and major factor is health services provided at the HFs, for example, people with the CBHI card visiting HF come only with their cards and all services at the HFs such as cards, lab investigations, drugs, and all needed investigations will be given and gets registered without payment but payment will be dealt later by CBHI…the problem is that they {HF workers} see them as if they are free services, however, this will not leave unpaid as woreda, zone, or kebele will pay later on. There is a misconception that CBHI members are free users or assumed to if donation beneficiaries. Eh…they give more priority to cash payers and focus on them whereas, CBHI members are assumed as a free user. Eh…if you create such differences at health facilities the dropout rate increases.

I: Would you explain what you mean by “CBHI members are seen as free users or assumed as donation beneficiaries”?

R: when I say free services it is not free but it will be paid to them later as the treatment is a contractual agreement. Free service users are already known, for example, there are drugs like TB or MCH services that are given for free for all patients whether they are members or not, and they equate CBHI members with such free users. Eh…CBHI patients are regarded as loan patients and previously there was a problem as the old trends sustained for years. There is a high tendency to want cash.

However, recently there has been improvement in managing CBHI users even high priority given to them. Eh…but now high numbers of health facility visitors are becoming health insurance users. This is because covering living expenses by itself is becoming difficult and people turning to CBHI membership. Since the community understands the benefits of health insurance, eh…majority of users in our hospital are CBHI members. Eh…the dropout rate will still be high if health care services such as lab, drugs, and investigations are inadequate. Eh…I mean a low renewal rate, but new membership may increase still. Eh…HF plays a major role in CBHI enrollment. If services such as lab investigation, other investigations, and drug supply improve at HFs, CBHI enrollment will get higher.

This is just like you wanted to buy something from a shop, but one shop is full of materials for sale while the other one is empty, and you will prefer the one with all the needed materials. CBHI is aimed at reducing patients suffering by looking for money for treatment and getting what they need in a timely, however, HF is the main factor for the dropout rate of CBHI memberships. We usually conduct service satisfaction assessments, and we improve services based on the findings, for example, drugs prescribed outside the health facilities should be minimized.

I: In your opinion why do people join CBHI? Is it because they are interested in CBHI or the increased cost of treatment at health facilities, living expenses, and the community could not afford it?

R: We can see it in two ways…one…In the early days of CBHI people enrolled by the information feed by the kebele or health professionals…. like the community accepted and believed in the information and objective of CBHI. Eh…(laughter), if you see the current tendencies eh…payments in private facilities are not affordable and living expenses are unimaginable. Eh…when we say health insurance it considers low-income groups, farmers.

So, one of the reasons for improvements in CBHI enrollment is cost of living is increasing; the second reason is health care services delivery, people prefer to stay at their homes than visit HFs without adequate services. For example, people like to get all services paying 400 or 600 birr at the CBHI memberships. What can you do when you are sent for every service to the outside/private facilities though you have paid 600 birr? Therefore, the services we provide, the present cost of living, and thirdly interest determine the CBHI enrollments.

I: What other factors facilitate, think of mothers in reproductive age groups, motivate people to join CBHI?

R: Ay…there may be several factors helping reproductive age groups to join CBHI services. One of these factors is the increasing number of family members and this could lead to a lack of treatment for all family members. Eh…if your two children get sick you may not treat them both due to your financial incapability. CBHI encompasses all family members, if you pay 600 birr then all members of the family will be benefited. Therefore, one mother who had a CBHI card, even if, she had a husband, once she paid the money secured her utilization.

Another factor is economic issues eh… some individuals are unable to pay even 400 birr and suffer from diseases at home they are called poor of the poor (indigent family), however, if they are 10,000 out of 50,000 community members the government will cover health insurance for these poor of the poor. Eh…the poor community enrolls in CBHI after carefully screening and enrolling where they live, those in the woreda at woreda level, kebele at kebele levels, and urban at urban settings, and no poor will be left without support. Therefore, both the poor and those who are capable of contributing money will be included and this method will help increase enrollment in CBHI. For example, we sometimes see people who complain about the treatment cost because either they do not have adequate money or CBHI memberships, when asked for CBHI they are not informed of it and later they come with memberships and mobilize others to join. In the end, those who pay for health care services from their pocket will gradually be pushed to health insurance as long as we improve services at HFs.

I: would you tell me other factors preventing people from joining CBHI?

R: First one is economy, eh…second one is lack of awareness; eh…another point is religious belief saying GOD protects me from disease, not the money although this is not a bad idea. People say *am I not a Christian?* They say why I joined the ‘Ikub’ for my tomorrow’s health. Another point we believe is the biggest factor is the health facility services problem. Eh…some services were not included in CBHI services, for example, dialysis, eh…cosmetics, a person may want to polish his tooth, eh…procedures like renal transplantations which are very expensive not included, however, it will be revised in the future, I think so. Hence, religion, being low in economy, cost of living, services at health facility…this (HF service) takes huge share, CBHI package… some people say what does it cover if it does not cover that and this like the dialysis…and lack of awareness determines enrollment. The money we collect from members can’t cover the aforementioned service’s cost.

I: Would you explain moral hazard (the tendency of insured individuals to overconsume healthcare services because the costs are covered by the CBHI insurance)?

R: eh…CBHI members ' health-seeking tendencies have increased and this is the main gain and success, because, nationally and globally everyone is expected to have a medical checkup twice a year. But eh… some CBHI members practice frequent visits to the health facilities as they are not expected to pay for services. For example, a person with a headache that may subside by itself shortly could come to HFs as he thinks the treatment is free and also, he has CBHI cards. This is good in one way because health-seeking behavior improves, whereas, it will cause repeated visits, and looking for drugs more time, for example, DM patients taking insulin drugs revisit HFs to take more insulin as it will also be used for other purposes. Eh…he may sell it for others with money.

I: what has to be done to control this (people taking and selling the drug) issue?

R: we are making the control mechanism more electronic, for example, when a drug is prescribed for a patient, it is by the system not with paper that shows when the patient comes, what drugs were taken, why it was over, etc. This overutilization of HFs comes as a result of the CBHI system because people believe that it is for free or else the CBHI scheme would pay for them. Eh…CBHI recommends patients first get health care services at health centers, then primary hospitals, and through a referral system health center to primary hospitals, primary hospitals to general hospitals, and general hospitals to referral hospitals hierarchically. Eh…what we are observing is bypassing health centers for simple illnesses and visiting hospitals; eh…we also observing even by passing both health centers and primary hospitals to visit general hospitals. Eh…the issue of repeated visiting of HF is also a problem in our hospital, but you cannot say you have no illness for the person complaining of illness, however, we are checking for the frequencies and drugs taken to reduce redundancy. This will create a great burden on the HFs. Eh…we check in for those coming repeatedly to manage this problem, for instance, we have identified a patient revisiting 4-5 times per month.

I: As you have mentioned above, what are the benefits of using the health tier system for CBHI?

R: For example, assume a patient at Tula comes to Adare General Hospital by passing *Tula* Health Center, *Tula* Primary Hospital, Motite Fura Primary Hospital will affect patient time, and transport cost, however, his perception that better healthcare will be given at Adare hospital is good, but he may not get services here as he bypassed the health hierarchy. Therefore, there is an impact on patients like time, transport costs, etc. Therefore, strengthening the hierarchical treatment system is mandatory as the patient will be benefited more.

I: Would you please mention CBHI benefits for mothers only?

R: Eh…CBHI has better benefits for mothers; eh…male involvement in healthcare utilization of mothers is less due to our customs. Yaw…when we see Ethiopian and our region’s condition, we can observe male dominance and the economy is in the hands of males. Eh…I want to stress that mothers are the first to reach HF to say their child may get sick in the absence of their husband because she has a CBHI card, and more burden is on mothers. You can take our hospital and most of the children admitted to our ward were accompanied by mothers. Eh…CBHI helped women seeking health care for their sick children, or when their husbands were sick or they were sick. Eh…no waiting for their husbands for money or decisions. If we move around, we can observe mothers coming with CBHI cards on their hands, be it for DM treatment, but husbands may not accompany them; it also has benefits for their health, for immunization eh…, others have better relationships with their children. By the way, when they join CBHI they may enforce their husbands; eh…they may say ‘I will sell my chicken if you fail to pay’, eh…I came across mothers saying to their husbands ‘What we can do with 300 birr?’ Another point is males usually resist new ideas, whereas, women are faster to accept them and they are closer to health such as ANC, child vaccination, buying drugs, etc.

I: What do you suggest to improve CBHI enrollment?

R: Eh…primarily, keeping regular members of CBHI, secondly, increasing new enrollments because, the current payment for CBHI is not enough to cover healthcare costs such as laboratory, imaging, drugs, etc., therefore, we should increase the enrollment rate. For example, a person pays 600 birr per year, however, he utilizes far more than this assuming he comes 4 times per quarter and costs 1000 birr per visit he will consume 4000 birr means 3400 is negative for the CBHI scheme.

Therefore, to cover this negative balance, increasing the number of new members is mandatory, not the amount of payment. Eh…hence hastening awareness creation to improve members’ enrollment and as new members enrolled our saving balance improves much. This is true as the illness level would be lower compared to many members in CBHI. If the number of ill people increases, we may have a negative balance. In addition, revising the CBHI manuals, directions, and rules and updating them with the current scenario is essential.

For example, increasing the present amount of money for members; another point is modernizing the CBHI program, for instance, a person in the *Tabor* sub-city could be identified as a CBHI member wherever in the country, I mean interconnecting with the use of technology is a good method. In summary, modernizing the system, increasing the numbers of members, and above all improving the capacity of health facilities with infrastructures, for example, a person should not be referred to Addis Ababa for the sake of CT-Scan which exposes him to extra expense and availing CT scan at Adare Hospital will improve the capacity of the facility. Eh…another big issue is developing health facilities inputs starting from health centers and health centers must be strengthened to keep the system endured. Eh…primary hospitals should be equipped with inputs and if this is more than 85% of patients will use them. Another is to let health facilities be strengthened, provide improved services, eh…modernizing their services, for example, card provision in our center is going to be paper free to reduce crowding. Generally, improving and strengthening health facilities and services and modernizing them will increase CBHI members, and this will increase the financial capacity which in turn develops into the main system.

Another point is we provide services every month, but we receive the money after three months, 5 to 7 million birr utilized by members and you have to wait for all three months, in other way drug in nature should revolve and be replaced soon that you sell and then buy it just like a shop. Therefore, the system to replace the money should be modified. What you will be paid after three months is post payment and has a long procedure to be completed as you will send the report to offices, the office checks from each sub-city, then you take to CBHI agency which also takes time, in general, you will get payment for the third quarter in the fourth one or first quarter payment in the second one, during this procedures HF will weaken.

Therefore, this system will result in weakening the health services and should be modernized. Previously, HFs were allowed to request 75% of the payments whereas, the rest will be released after auditing, however, I suggest releasing 100% of the payment at each quarter, in case the problem exists, it could be deducted from the next budget which is the case in Oromia region, so that the HF will be better in-service delivery.

The second method is designing a pre-insured method, for instance, we usually make a contractual agreement with the woreda or the sub-city and I may receive 100 thousand or 150 thousand as a pre-payment and I will prepare the report monthly and they can check it and if I used 100K and 50K left unused, no need to ask for other money and they can say use it and ask us for any extra utilization, hence, the HF will have adequate services and patients will not suffer. HF facilities should be supported earlier, when I say, that sometimes HFs not fully functional may use the money for other purposes and this could be another concern. As an example, we have two sites in Bishan Guracha, The Oromia Region releases 100% of the budget, but in Hawassa only 75% of the CBHI budget will be released for HFs at a quarterly period. Eh…we are commenting on the Agencies in the Sidama Region to follow a similar scenario of 100% release.

I: Who is the main influential body on the CBHI program?

R: Eh…CBHI agency and Regional Health Bureau. The agencies and RHB have the authority to pass the legal and guiding principles regarding CBHI.

I: What issues on Agency and/ or RHB help or affect you more?

R: Yaw…presence of agency, for example, in Oromia agency is working up to the lower level and those we have seen are very good that they conduct auditing, the observe and support technology utilization at woreda levels as they are better in education, and transfer comments and feedback received from lower levels because they are responsible persons. This is the best idea and I support it if this is shared with our region as well. However, failing to observe the lower levels and not monitoring will affect the health facilities.

Eh…I know how much our HFs suffer at the woreda level, but the agency should go and check for woredas not paid or not completed payment for HFs. Even the individuals who agreed with HFs should be contacted and discuss the services and insurance issued by the agency and RHB; sometimes it is good to hear from customers as to whether the services utilized are acceptable or not. Working together for change is better through harmony.

I: Would you explain about CBHI premium, who are being enrolled more to the program?

R: Eh…people who ever faced problems and suffered from illness are the more likely to join the scheme because they know how much it costs and know the situation at HFs. Those who were in the treatment condition joined without any hesitation. Eh…individuals with hypertension are the commonest in buying CBHI as they are unable to pay at a private level. Those who are poor the poor for whom woreda is paying are also the fastest in renewing and joining the CBHI. However, the rest community observes the conditions for joining the health insurance.

I: What impact does this type of people enrollment have on the CBHI? What positive or negative side does it have?

R: Interims of benefit these people were saved from expenses, as an example, DM patients always get priority and we provide them with the drugs from any source. Perhaps, there would have been interruptions in taking drugs before they joined CBHI, but no such gaps would exist and they would have benefited from CBHI membership. Eh…currently, people with chronic diseases like hypertension and DM make frequent visits and get checkups more, but there was an expense every time they visited HFs before health insurance. Eh…you cannot prohibit him if he wants to come twice a day. Perhaps, frequently taking drugs may result in drug dependence which will affect their health. There is a burden on HFs enrolling such people.

I: Anything worth mentioning concerning health insurance?

R: I have no more to add but to strengthen what I have stated above. However, I will stress to the society that they have to use this opportunity and the enrollment rate should be emphasized and more mobilization on the utilization of the CBHI program. This is because, at woreda levels, the administrators are in treble due to low membership enrollments, and increasing new members will solve the financial shortage, as an example, Hawassa City could collect about 20 million birr a year and this is quite adequate for CBHI that could save 5 million if expended 15 million a year. Eh… increasing public awareness, reinforcing the guidelines, and updating the system as in Hawassa where bar codes are being used which helps to scan and differentiate the sub-city type. Eh…computerizing the system from *Kebele* to HF, even up to black lion hospital that patient’s information should be accessed at all levels. Even the financial system should be interconnected and governed under a unitary system. For example, rather than saving money by sub-city, it is good to organize under zonal or one centralized center that will easily be distributed to HFs and this will be used to cover the shortage in one sub-city in case the saved account is over.

I: Thank you very much

R: I also thank you

**KK6: Key informant interview 6**

**Interviewer: thank you for your willingness. I am an RA from Hawassa University, I am going to ask you questions regarding CBHI. My first question is what you can tell me about CBHI**

KI: CBHI is a service where the communities get health service coverage with minimum payment. It helps people who can’t afford medical care to get treatment by being a member of this service. In this hospital, from the total number of people who visit this hospital in a day more than 60% are CBHI users.

**Interviewer: who are the majority of users of CBHI?**

KI: well, there are criteria set by the government to be a CBHI member. People who don’t have a stable source of income which means excluding government employees are eligible to be members. It is not our concern to determine who should and should not be members, we are service providers

**Interviewer: you have told me that more than 60% of patients that visit the hospital in a day are members of CBHI, have you noticed any discrepancy among the number of users for example based on their type of disease, economic status, and so on?**

KI: most of the participants are those who don’t have the financial status to visit private health facilities, with no stable income source, and who are in middle and low economic status. Every individual from young to old can get the service. Some might get services that cost 140birr, 500 birr, or as high as 50,000birr, 60,000, or might just visit for evaluation (checkup). Most follow-up patients are people with chronic diseases like, DM, hypertension, those heart disease, or nerve problems. They don’t have the economic status to buy the medication and since the medications should be taken for life long, they are the most users.

**Interviewer: do you think there might be a burden on the CBHI due to the majority of users being chronically ill?**

KI: As I have told you there are people who get evaluation services as well as those getting services that can cost up to 60,000 birr. I don’t think there is an additional impact on the government but this can help the community b/c the aim of health insurance is to provide service to people through financial contributions from a large number of people**.** And the people (with chronic illness) are advantageous.

**Interviewer**: **is there any limit on the types of services provided by the health insurance?**

KI: some services can and cannot be covered by health insurance. For example, a road traffic accident (car, motorcycle), that can have insurance coverage by 3^rd^ party cannot get coverage by the health insurance. Second, if a person visits the hospital for an eye checkup and glasses are ordered, the health insurance only covers the cost of the checkup but not the glasses, and if a person can have a dental checkup using the insurance the insurance will not cover new teeth implant**.** generally, cosmetics surgeries, people on dialysis, and any issues related to war are not included in the agreement. Other than this there are no limitations, anyone can get the health service without limit.

**Interviewer**: **As you have told me people who use the insurance are those with low economic status. What do you think are the reasons for people with good financial status not using CBHI?**

KI: the first reason can be because of the criteria set for CBHI membership. As I have told you the service also does not include government employees and people who have stable income. there is another system called HSI that includes them but have not been started due to different reasons. The 2^nd^ thing is people who have better income visit facilities they assume have better service. This is not due to low-quality service in government institutions but there can be longer waiting times since people are treated without any discrimination, whereas in private facilities people who can pay can have the services without waiting for long.

**Interviewer: Have you noticed any discontinuation of CBHI membership among the patients**

KI: No, we haven’t noticed that, because they are happy. They share their experiences and benefit from CBHI with others after getting treatment and getting cured. Additionally, the woredas use this person to disseminate the benefits of CBHI to other community members. I have not noticed anyone who was not satisfied with the health insurance.

**Interviewer: Have you noticed inappropriate use of the insurance by the members**

KI: that can happen. Sometimes people visit the hospital for the simplest complaints. For example, diseases that can be treated at health centers, primary hospitals, and general hospitals level visit the hospital without passing through the referral system this is due to a lack of integration in the system. We make agreements with the woredas, zones, or regions as a pool.

We are service providers. The regulations that are provided to us are not appropriately addressed to the zone hospitals or woredas. Health centers and primary hospitals cannot directly refer to comprehensive specialized hospitals. Only general hospitals can refer patients to us. This is not properly addressed to all. Some health centers that are found in very distant areas refer patients to us. The patients might face difficulties due to this. Sometimes people want to get services based on their preference and not the professional, the other thing is some people want to get services that are not covered by the insurance like those I have mentioned before. We try to get awareness to the individuals.

**Interviewer: my next question is regarding the payment system what is the CBHI payment process?**

KI: the health insurance payment is made once a year among the members in every Woreda and city. For example, a person in the Oromia region might pay 960 birr, which was 450 birr last year. This payment is made on a yearly base additionally 20% is added to the pre-family number. The payment will be collected in the CBHI account. The members will be told when to renew their membership and if there is an increment in the fee. We send reports on quarterly base to the Woreda that has an agreement with the hospital. For example, the first quarter is from Sene,1 to Meskerem, 30. We write a letter to the woredas that states the total cost including many patients and services provided during the time. Then they conduct a claim audit by crosschecking patients’ cards and the service claimed to be provided. Then payment is made to the hospital. 75% of the payment is paid when requested and the rest 25% is made after the claim audit which is called onsite verification payment.

**Interviewer: have you faced any problems concerning the payment?**

KI: the main problem of the health insurance is the payment system. The service payment is delayed. We have an agreement with around 130 woredas. They don’t pay in time; some woredas delay up to 6 months to a year affecting our medication, laboratory regent supply, and the quality of service we provide. This is the biggest problem. Also, extra time payments to the professionals cannot be made b/c of low internal revenue; affecting the morale and motivation of the professionals.

We give notice 3 times to woredas that don’t pay indicating the impact of delayed payment on the quality of service provided by the hospital and the time limit for them to pay. If they don’t pay, we stop providing the service for members who visit from the woredas.

**Interviewer: this is a referral hospital providing service for more than one region. Are all the agreements made with the woredas or due you made agreements with the regions?**

KI: there are 3 types of agreements. The concept of the agreement is the same but can be made at different levels. There is an agreement made with each Woreda, a zonal pool that contains all woredas under it, and a regional pool made by the region’s health office for the woredas found under it. The report is sent to respective levels agreement is made with.

**Interviewer: which do you think is better or does getting into an agreement with the different levels have no difference?**

KI: It is 100% better to make the agreement with the Woreda b/c you will communicate specifically with the Woreda and if the problem occurs the Woreda is accountable you will stop service only to the specific Woreda. When it is at the zonal level it will even be hard to get a meeting with the governing people. And this is worsened at the regional level. The payment system and source of money are hard to know.

**Interviewer: what strategies do you think can solve the problem related to the payment delay?**

KI: the first thing is the health insurance budget should be separated. The biggest problem is that the health insurance money is used for other purposes. If the insurance money was solely used for the insurance this problem would not have occurred. Therefore, it is better to create a system that uses the insurance money only for community-based health insurance. The other thing is there should be an autonomous organ that can improve commitment regarding the payment.

**Interviewer: how do you think the community perceives the benefits of CBHI?**

KI: from what we have noticed through people who visit the hospital, we understand that they are benefiting. Patients say that they are benefiting from the health insurance and are saved from large amounts of payment. There might be some complaints sometimes regarding medication supply, and outsourced services, for example, if the medication is not available in the hospital and the patients are asked to buy from another place they might complain. These things can be improved. We can’t do anything about that, but we take comments to improve and work on it. Other than that, some medications are imported and we face these shortages as a country.

**Interviewer: are there any additional h benefits women can get from CBHI?**

KI: Yes, there are, as you know most women in our country are economically dependent on men. Therefore, when they need medical care or are sick, they rely on their husbands. If the husband does not have money they will have to wait until he can provide, but if they have the CBHI book they can get health care in health facilities so without question it has benefits for women.

**Interviewer: what do you think are the reasons for people not to get a CBHI membership?**

KI: the first thing is not being permitted by the CBHI law (government employees), the second reason is some people relate it with politics, saying why I am not being treated. Also, some services are not covered by the insurance and others are exempted services like MCH care, and treatment for malnutrition so the rest 40% of patients are including this.

**Interviewer: what strategies do you think can increase CBHI enrollment?**

KI: disseminating information about the benefits of CBHI in different community gatherings, Media outlets, and schools, ider, preparing broachers can help. Also sharing experiences of people who had benefited from CBHI is good. For example, before CBHI, when people got sick and had to be admitted for a long time they would have to sell, their house, cars, and other assets to get treatment but now health insurance can cover medical services in the hospital without any limit starting from the woreda health center to Addis Ababa(referred). If people understand the aim, it can be increased

**Interviewer: what can you tell me about the organization of CBHI?**

KI: The members are enrolled at the kebeles and report collectively to the Woreda. Then the Woreda will report to zones. If the exact number of members is known the government also provides a subsidiary concerning the number of members. But most of the time this number is not properly reported or it can be falsified. For this reason, the agency intervenes up to the Woreda level to check the exact number of users. this will help to know the CBHI capital and help for evaluation.

**Interviewer: my next question is regarding leadership; how do you think leadership affects the implementation of CBHI?**

KI: to effectively implement health insurance the leader should first fully understand the aim then he/she can create awareness for others. If that is not the case the leader cannot lead properly. The next thing is being committed and motivated to think of others' problems as own. The third thing is being free of corruption. The money collected should only be used for health insurance and not for other political and personal uses.

**Interviewer: I am done with my questions is there anything you want to add?**

KI: health insurance is important to the community creating good awareness and increasing enrollment so leaders should work on that. Also, the service payment should be done timely so the health facilities can provide good service. The other thing is people complain about services that are not covered by the insurance the right information should be given.

**Interviewer: thank you.**

**KK7: Key informant interview 7**

Interviewer: Could you tell us what you know about CBHI?

KI፡ Thank you, Health insurance started in our kebele around 2010 E.C and when I started, we were taught the importance of community-based health insurance community now I am a member of health insurance and at the beginning, the registered community was very small, but seeing the community that used health insurance, the number of people who became members increased. In addition, the fee is fair and most of it is covered by the government. All members of the community who are eligible for health insurance can be annuity regardless of gender.

Interviewer: Is there a dropout from CBHI? Why?

KI: Thank you, In the current context of our Kebele community health insurance, some people have left and are still leaving (some people are terminated) due to the lack of quality health institutions, especially if we get a card and laboratory services after a lot of suffering (after long torture), we are ordered to buy medicine from a private pharmacy. Health professionals give priority only to those who have money in their hands. Health insurance members therefore come everywhere to get money to buy from private pharmacies. If the problems I mentioned above are solved, all the residents of our reception will be members.

Interviewer: Premium and its management

KI: Good, the collection of the CBHI money is done by the Kebele management. After giving the collected money to the health extension along with the receipt, they will deposit it in the bank and report to the district health office. However, since the collected money is not paid on time by the health institutions, we have not received services since April.

Interviewer: Is there a tendency of insured individuals to overconsume (Moral hazard)

KI: Good, we only go to medical facilities when we are sick, but even when we are sick, we often do not get services because we do not get a book (ID card) in time after paying money

Interviewer**:** Is there adverse selection

KI: Good, according to the education given by the health experts, all sections of society can enter regardless of whether they are poor or rich. But in our Kebele, the lower income group has the upper hand over the rich, because the lower income earns money only once a year

Interviewer: what is the community’s Perception of CBHI specifically concerning women?

KI: Good, Health insurance is very important and the community is very well aware of it as well as women are becoming more and more aware of the importance of health insurance in society. In addition to this in our Kebele, women and men can be members without any difference regarding membership of CBHI (both have equal opportunity to register)

Interviewer: What are the factors that you think can facilitate household enrollment and continued membership?

KI: Good, to ensure that everyone is a member of the health insurance system, the health providers will be provided in a timely manner and providing quality health services to those who need health services in health facilities and transferring the money to health facilities in time.

Interviewer: What are the barriers to enrollment in community–based health insurance?

KI: Good, what are the things that prevent everyone from becoming a member of health insurance? The fact that members don't go to health facilities and get the necessary treatment is seen as an obstacle in our kebele and prevents new members from being added. Why should I say that when people interact with each other, they exchange information? For example: - A member who goes to health facilities and gets good treatment as a member of health insurance will take it on his positive side. However, members who have gone to the health facilities and not received adequate services are negatively affected by sharing negative information, which is hindering the number of members from increasing.

Interviewer: What strategies could be implemented to increase enrollment in community-based health insurance?

KI: Good, we have been able to increase the number of CBHI members in our kebele by using those who have gone to health facilities and received all kinds of services as strategies.

Interviewer: Can you describe the organizational structure of the CBHI?

KI: Thank you, Health insurance is led by the WHO, HEW, PHCU, and the District Administrator, but strong decisions are taken by the district administrator

Interviewer: Are there any challenges or benefits associated with this hierarchy?

KI: Even though we repeatedly told them the problems we encountered during the visit to the health facilities, they were not solved by the political leaders. In general, the problem of ownership and commitment is related to the organizational structure of CBHI

Interviewer: What are your thoughts on the leadership of the CBHI?

KI: Good, in order to achieve the impact and success of the CBHI implementation, all the leadership needs to work together to enroll the entire community and monitor and supervise the health professionals.

**KK8: Key informant interview 8**

Interviewer: Could you tell us what you know about CBHI?

KI: Good, it is a system where a family can get medical treatment for the whole year by paying money only once a year and political leaders and health professionals make us a voluntary member, not a compulsion, but the service provided to us by the health organization is not consistent with what we were told.

Interviewer: Is there a dropout from CBHI? Why?

KI: Good, The people in the kebele where I live are going out of CBHI, because when the health providers see the CBHI book in our hands (ID card), they tell us that medicine is not available in the institution and send us to buy medicines outside the government institution, but people who have money in their hands can get the same medicine as our medicine in the government institution but only we got minor health care services from public health facilities.

Interviewer: Premium and its management

KI: Good, the money is collected by the receipt, and after the collected money is deposited in the bank, the slip is given to the district health protection office. In addition, payment of CBHI funds to the contracted health facility is provided through the health office.

Interviewer: Is there a tendency of insured individuals to overconsume (Moral hazard)

KI: Good, we only go to medical facilities when we are sick, but health is constant, and then the year ends without ever going away

Interviewer: Is there adverse selection

KI: Good, according to the government policy, all people can be covered by CBHI, but in our kebele, it is the low-income people rather than the rich people because the low-income people rarely get money.

Interviewer: what is the community’s Perception of CBHI specifically concerning women?

KI: Good, in our Kebele, women, and men can be members without any difference regarding membership of CBHI (both have equal opportunity to register and still I haven’t heard a negative perception from our community

Interviewer: What are the factors that you think can facilitate household enrollment and continued membership?

KI: Good, Community health workers from the village should be well trained to make the women members of CBHI and money should be given to those who do not have membership fees.

Interviewer: What are the barriers to enrollment in community–based health insurance?

KI: Good, both of them (women and men) participated equally in any health program including CBHI’s in our kebele

Interviewer: What strategies could be implemented to increase enrollment in community-based health insurance?

KI: Good, the importance of CBHI is being used as a key strategy by health providers to educate the community by using those who got quality health care at different health facilities.

Interviewer: Can you describe the organizational structure of the CBHI?

KI: Thank you, Health insurance is managed by the WHO, HEW, PHCU, and the district administrator, but strong decisions are taken by the district administrator

Interviewer: Are there any challenges or benefits associated with this hierarchy?

KI: Thank you, even though we repeatedly told them the problems we encountered during the visit to the health facilities, they were not solved by the political leaders. In general, the problem of ownership and commitment is related to the organizational structure of CBHI

Interviewer: What are your thoughts on the leadership of the CBHI?

KI: Thank you, problems arising in health facilities should be resolved as a priority for CBHI members, but if this is not resolved, CBHI members will leave this program

**KK9: Key informant interview 9**

Interviewer: Could you tell us what you know about CBHI?

KI: Thank you, As I heard from HEWs about the importance of CBHI, an HH can register only once a year and get any health service for a whole year without paying any extra registration fee. I was also a member of CBHI for the last 2 years but now I am not a member of CBHI as I am not getting any benefit

Interviewer: Is there a dropout from CBHI? Why?

KI: Good, As I have heard from health professionals, I am registered as saying that if you become a member only once a year, you will get any health services, but when I visited the health facilities, what I experienced was the opposite and that I only got card and laboratory services and We take the sick person, but we are told that there is no medicine by the public health facilities, and we take that sick person again and we come to our house in search of money because we don't have money in hand means all the prescribed medicines were bought from a private pharmacy therefore I left the program**.**

Interviewer: Would you like to rejoin CBHI again?

KI: Good, the opportunity that the government has given is good, but according to what has been said, the government should take corrective measures against the professionals who are blocking us from getting proper treatment in the health facilities. I believe medicine is not lost in health institutions, but because they open their own houses outside and send medicines and patients to their pharmacies for their use. Therefore, because we paid our money and did not get enough medical services, we stopped. If the problems that caused us to leave the top are solved by the relevant body, I will not be the only one, but I will coordinate others and I will also make them members.

Interviewer: What are the factors that you think can facilitate household enrollment and continued membership?

KI: Good, the barriers with CBHI in our kebele were dissatisfaction with health care service during health facility visits.

**KK10: Key informant interview 10**

**Interviewer**: **Could you tell us what you know about CBHI?**

**KI:** Thank you when CBHI starts in Ethiopia for the first time it is ABT Global/USAID which has taken the initiative to support all stakeholders in every aspect including training. When it was started for the first time it was piloted in 13 woredas national: 3 in Tigray, 3 in Amhara, 3 in SNNPR, and 4 from Oromia. So, when it was initiated in SNNPR Yirgalem city administration from Sidama, Gabya woreda from Kambata, and Damot woyde woreda from Wolita were the pilot woredas.

So as CBHI is a community-based insurance to improve the health of the community we have worked to raise the awareness of the community, health professionals, and other stakeholders from the start. Since it was a new idea and a new initiative there were a lot of challenges from enrolling the community to the payment system. At the time of initiation, it was the health system who was the owner of the system but it needed a lot of work even to engage those in the health system to the CBHI system. The ABT associate has recruited staff to support this whole process.

**Interviewer: Enrollment of the community in CBHI**

**KI:** In terms of enrollment the principle of CBHI encompasses every community to system. The CBHI has principles guiding principles including sustainability, accessibility, community engagement, and others. Concerning this there are two types of membership. The first is those who contribute from their pocket and are a member of the CBHI system. The second one is the poor in the community who will be a member of the CBHI system as the government will cover their payments to the system.

This second system burdened the woredas and the lower structures in the identification of these populations and covering their budget. But when we see it now the enrollment of the community has increased gradually as awareness creation campaigns continue to be done at marketplaces, churches, and other informal associations. The advantage of this system is addressing poor household women to the system. In those initial days, the community starts to understand the benefit of getting treated with a one-time premium contribution to the system the client enrollment raises. On those days the challenge was a load of patient flow to the health facilities, especially on market days and the health professionals used to consider the CBHI as a burden.

**Interviewer:** **Are there adverse selection and moral hazards?**

**KI:** There were section biases, especially in the inclusion of those for whom the government will cover their payments. The woredas had a budget quota of poor people to include in the CBHI system and there were biases in the selection of these people from the community. To deal with this issue the government has arranged a budget coverage system where 40% will be covered by the region, 30% by the zone, and the rest 30% by the woreda. Therefore, every poor can benefit from the CBHI system. This is the system we have established at SNNPR and all the regions including Sidama are following the system after the dissolution of SNNPR.

Of course, adverse selection is the challenge of every insurance and CBHI also suffers from that. Both risk and adverse selection are present. The system comes for those who are not part of the regular average community and benefits those who are merchants, farmers, and the poor. Especially in urban areas adverse selection is being seen seriously. This is because the health-seeking behavior is high in the urban community which results in overutilization of the services in the health facilities to the level the urban scheme enters to bankruptcy.

This utilization can be associated with a higher magnitude of non-communicable diseases in the urban community. The other issue as health insurance generally is that we do not check the health status of the person before enrollment to the CBHI. Therefore, we don’t know who is becoming a member of the system. As a principle, everyone should be a member and those who need treatment will get treated but if everyone who joins the system is ill then they will overutilize the system, and this will be a challenge. But the one who prevails is the adverse selection in the cities.

CBHI schemes in cities like Yirgalem, Wolaita Sodo, and Worabe are in debt and they commonly ask for the next year’s premium to pay to the health facilities. Now they are working to widen the base of the CBHI which will help them to include all the community that In turn help the increase those who are healthy to be a member of the CBHI. The number of households in the urban community is few and predisposes them to adverse selection, and bankruptcy.

**Interview: Premium contribution and its management**

**KI:** The premium is collected from the community through the kebele and the premium will come to the CBHI scheme organized at the woreda. So first the kebele leader will come to the woreda scheme and take a finance receipt to collect money from the community members upon registration for CBHI membership.

Of course, there are deficiencies in the return of the money to the finance at the scheme in the woreda but these days as those who collected the money are becoming accountable after the audit, the return of the money has been improved. After the money is collected, they will deposit it to a bank account opened by the Woreda CBHI scheme and bring the bank slip to the Woreda scheme for audit.

When we come to the process of payment to the health facilities that are contractual agreements of CBHI the client should be checked for membership and the services he gets will be registered at different spots like OPD, laboratory, and pharmacy. Based on this registration clinical and claim audits will be done. The claim audit is about the relevance of the expenses for the treatment given as the claim audit will just see the financial procedural appropriate. This way the audits will be done and the woreda or the respective governmental CBHI Scheme will pay to the facility as per the agreement.

**Interviewer**: **What is the communities’ perceived benefit of CBHI?**

**KI: T**he community started to understand the benefits of the CBHI gradually. As I have mentioned earlier it was challenging when it was initiated but later as the community member started to benefit from the package the community started to understand the benefit and the enrollment increased. But it still needs to work on the awareness of the community.

**Interviewer**: **What are the factors that you think can facilitate household enrollment and continued membership in community-based health insurance?**

**KI:** Well the first opportunity is the presence of a variety of social networks to be utilized to raise the awareness of the community. The networks can include edir, religious institutions, one to five structures, and others. Even the health extension structure is an opportunity to enroll the community members. The other is communities’ experiences of CBHI. Community members promote the system once they have got a good service. On the other hand, they may be promoted negatively if they are not satisfied with the service they have received.

**Interviewer**: **What are the barriers to enrollment in community-based health insurance?**

**KI**: There are a number of barriers from the demand side including a low level of awareness of the community, poor method of identification of the poor people from the community by the woredas, and shortage in budget allocation for the poor in the woreda, poor health information system at the woreda scheme.

From the supply side of the system, there is a poor quality of service in the health facilities including a shortage of drugs, laboratory agents, and unethical professionals. The facilities raise problems on EPSA as a cause for the shortage of drugs in the facilities but EPSA doesn’t accept mentioning that it is because the facilities do not come up with RRF that they don’t get drugs timely. This compounded by budget shortage has resulted in drug deficiencies at facilities.

**Interviewer**: **What strategies could be implemented to increase enrollment in community-based health insurance?**

**KI**: Well we have to raise the awareness of the community. But it should not be through campaigns but rather in regular awareness mechanisms so that the community will take it as other regular services like a school registration period in September. From the supply side, the facilities shall work to increase the supply of drugs and improve the service quality. The insurance and the health system should be completely separated. Because it is even difficult for accountability because both the service provider as well the service utilizer are under the health system accountability cannot be ensured. So, both the demand and supply sides are being ruled by one office by one VBHI board.

**Interviewer: Can you describe the organizational structure of the CBHI in your community and its effect on function?**

**KI**: The scheme in the woreda is the main structure of the CBHI system. The scheme has a health professional, health informatics, and finance expert. The scheme coordinates all the activity of the CBHI and plays an important role in making the poor and the needy a member of the system. The scheme in the woreda works under the woreda’s CBHI board. The boards are chaired by the city mayor or woreda administrator.

**Interviewer: What are your thoughts on the leadership of the CBHI?**

**KI:** One of the successes of the system is reaching this level from a pilot of three woredas to including all woredas in the SNNPR region. The other is a regular CBHI schedule for enrollment of the community members, premium collection and deposit are created from October 8 to January 7 this is a success from the leadership. This is true for all former SNNPR regions including Sidama. The other success due to the good leadership is an increased resource mobilization which reached five hundred million due to enrollment of the community to the system. This money is being pooled to support the health care system in addition to the regular government budget.

On the other hand, there are issues which need a better leadership. The first is the awareness creation movement is commonly done as a campaign for a limited period in some areas which is not successful and needs regular attention. This of course happens in some areas and varies from woreda to woreda with the leadership in place.

Then the other point is poor premium collection and management which goes up to taking legal accountability measures on the CBHI leadership at Woredas. The other big problem in the leadership is the leaders at health facilities who look to the CBHI as an external body to exploit financially. These frauds are seen commonly for example they will do like... mm copy-pasting the name of a community member who gets service at some month in the quarter to another month in a quarter to increase their number and the money the facility could get.

Though the sense of ownership of the leaders at the facility is good acting wrongly like this is illegal and a wrong perception towards CBHI. The other gap in the CBHI leadership is inconsistency and misses of board meetings. As a principle, the CBHI board should meet quarterly but most don’t do that and a few even don’t meet in a year. Concerning this even though the meetings are done regularly the meetings are not problem-solving. The problems we see this quarter continue to be a problem in the next quarter and beyond. These meetings show the presence of problems in decision-making and bringing solutions to the problems.

**Interviewer: Is there anything would you like to add?**

**KI:** Thank you, one issue I would like to raise is the fragmented pooling of premiums which is being solved and should be solved permanently. Previously sub-cities used to collect premiums separately and serve its community but now the premium is pooled to the city administration CBHI account. This has solved the problems of some sub-cities with a small number of members as they were suffering from deficiencies in the money which is now balanced by other sub-cities with better members. This is implemented in all sidama woredas and the pooling is now changing to zonal and regional pooling with new initiatives.

**KK11: Key informant interview 11**

**Interviewer**: **Could you tell us what you know about CBHI?**

**KI:** Thank you when CBHI starts in Ethiopia for the first time it is ABT Global/USAID which has taken the initiative to support all stakeholders in every aspect including training. When it was started for the first time it was piloted in 13 woredas national: 3 in Tigray, 3 in Amhara, 3 in SNNPR, and 4 from Oromia. So, when it was initiated in SNNPR Yirgalem city administration from Sidama, Gabya woreda from Kambata, and Damot woyde woreda from Wolita were the pilot woredas.

So as CBHI is a community-based insurance to improve the health of the community we have worked to raise the awareness of the community, health professionals, and other stakeholders from the start. Since it was a new idea and a new initiative there were a lot of challenges from enrolling the community to the payment system. At the time of initiation, it was the health system who was the owner of the system but it needed a lot of work even to engage those in the health system to the CBHI system. The ABT associate has recruited staff to support this whole process.

**Interviewer: Enrollment of the community in CBHI**

**KI:** In terms of enrollment the principle of CBHI encompasses every community to system. The CBHI has principles guiding principles including sustainability, accessibility, community engagement, and others. Concerning this there are two types of membership. The first is those who contribute from their pocket and are a member of the CBHI system. The second one is the poor in the community who will be a member of the CBHI system as the government will cover their payments to the system.

This second system burdened the woredas and the lower structures in the identification of these populations and covering their budget. But when we see it now the enrollment of the community has increased gradually as awareness creation campaigns continue to be done at marketplaces, churches, and other informal associations. The advantage of this system is addressing poor household women to the system. In those initial days, the community starts to understand the benefit of getting treated with a one-time premium contribution to the system the client enrollment raises. On those days the challenge was a load of patient flow to the health facilities, especially on market days and the health professionals used to consider the CBHI as a burden.

**Interviewer:** **Are there adverse selection and moral hazards?**

**KI:** There were section biases, especially in the inclusion of those for whom the government will cover their payments. The woredas had a budget quota of poor people to include in the CBHI system and there were biases in the selection of these people from the community. To deal with this issue the government has arranged a budget coverage system where 40% will be covered by the region, 30% by the zone, and the rest 30% by the woreda. Therefore, every poor can benefit from the CBHI system. This is the system we have established at SNNPR and all the regions including Sidama are following the system after the dissolution of SNNPR.

Of course, adverse selection is the challenge of every insurance and CBHI also suffers from that. Both risk and adverse selection are present. The system comes for those who are not part of the regular average community and benefits those who are merchants, farmers, and the poor. Especially in urban areas adverse selection is being seen seriously. This is because the health-seeking behavior is high in the urban community which results in overutilization of the services in the health facilities to the level the urban scheme enters to bankruptcy.

This utilization can be associated with a higher magnitude of non-communicable diseases in the urban community. The other issue as health insurance generally is that we do not check the health status of the person before enrollment to the CBHI. Therefore, we don’t know who is becoming a member of the system. As a principle, everyone should be a member and those who need treatment will get treated but if everyone who joins the system is ill then they will overutilize the system, and this will be a challenge. But the one who prevails is the adverse selection in the cities.

CBHI schemes in cities like Yirgalem, Wolaita Sodo, and Worabe are in debt and they commonly ask for the next year’s premium to pay to the health facilities. Now they are working to widen the base of the CBHI which will help them to include all the community that In turn help the increase those who are healthy to be a member of the CBHI. The number of households in the urban community is few and predisposes them to adverse selection, and bankruptcy.

**Interview: Premium contribution and its management**

**KI:** The premium is collected from the community through the kebele and the premium will come to the CBHI scheme organized at the woreda. So first the kebele leader will come to the woreda scheme and take a finance receipt to collect money from the community members upon registration for CBHI membership.

Of course, there are deficiencies in the return of the money to the finance at the scheme in the woreda but these days as those who collected the money are becoming accountable after the audit, the return of the money has been improved. After the money is collected, they will deposit it to a bank account opened by the Woreda CBHI scheme and bring the bank slip to the Woreda scheme for audit.

When we come to the process of payment to the health facilities that are contractual agreements of CBHI the client should be checked for membership and the services he gets will be registered at different spots like OPD, laboratory, and pharmacy. Based on this registration clinical and claim audits will be done. The claim audit is about the relevance of the expenses for the treatment given as the claim audit will just see the financial procedural appropriate. This way the audits will be done and the woreda or the respective governmental CBHI Scheme will pay to the facility as per the agreement.

**Interviewer**: **What is the communities’ perceived benefit of CBHI?**

**KI: T**he community started to understand the benefits of the CBHI gradually. As I have mentioned earlier it was challenging when it was initiated but later as the community member started to benefit from the package the community started to understand the benefit and the enrollment increased. But it still needs to work on the awareness of the community.

**Interviewer**: **What are the factors that you think can facilitate household enrollment and continued membership in community-based health insurance?**

**KI:** Well the first opportunity is the presence of a variety of social networks to be utilized to raise the awareness of the community. The networks can include edir, religious institutions, one to five structures, and others. Even the health extension structure is an opportunity to enroll the community members. The other is communities’ experiences of CBHI. Community members promote the system once they have got a good service. On the other hand, they may be promoted negatively if they are not satisfied with the service they have received.

**Interviewer**: **What are the barriers to enrollment in community-based health insurance?**

**KI**: There are a number of barriers from the demand side including a low level of awareness of the community, poor method of identification of the poor people from the community by the woredas, and shortage in budget allocation for the poor in the woreda, poor health information system at the woreda scheme.

From the supply side of the system, there is a poor quality of service in the health facilities including a shortage of drugs, laboratory agents, and unethical professionals. The facilities raise problems on EPSA as a cause for the shortage of drugs in the facilities but EPSA doesn’t accept mentioning that it is because the facilities do not come up with RRF that they don’t get drugs timely. This compounded by budget shortage has resulted in drug deficiencies at facilities.

**Interviewer**: **What strategies could be implemented to increase enrollment in community-based health insurance?**

**KI**: Well we have to raise the awareness of the community. But it should not be through campaigns but rather in regular awareness mechanisms so that the community will take it as other regular services like a school registration period in September. From the supply side, the facilities shall work to increase the supply of drugs and improve the service quality. The insurance and the health system should be completely separated. Because it is even difficult for accountability because both the service provider as well the service utilizer are under the health system accountability cannot be ensured. So, both the demand and supply sides are being ruled by one office by one VBHI board.

**Interviewer: Can you describe the organizational structure of the CBHI in your community and its effect on function?**

**KI**: The scheme in the woreda is the main structure of the CBHI system. The scheme has a health professional, health informatics, and finance expert. The scheme coordinates all the activity of the CBHI and plays an important role in making the poor and the needy a member of the system. The scheme in the woreda works under the woreda’s CBHI board. The boards are chaired by the city mayor or woreda administrator.

**Interviewer: What are your thoughts on the leadership of the CBHI?**

**KI:** One of the successes of the system is reaching this level from a pilot of three woredas to including all woredas in the SNNPR region. The other is a regular CBHI schedule for enrollment of the community members, premium collection and deposit are created from October 8 to January 7 this is a success from the leadership. This is true for all former SNNPR regions including Sidama. The other success due to the good leadership is an increased resource mobilization which reached five hundred million due to enrollment of the community to the system. This money is being pooled to support the health care system in addition to the regular government budget.

On the other hand, there are issues which need a better leadership. The first is the awareness creation movement is commonly done as a campaign for a limited period in some areas which is not successful and needs regular attention. This of course happens in some areas and varies from woreda to woreda with the leadership in place.

Then the other point is poor premium collection and management which goes up to taking legal accountability measures on the CBHI leadership at Woredas. The other big problem in the leadership is the leaders at health facilities who look to the CBHI as an external body to exploit financially. These frauds are seen commonly for example they will do like... mm copy-pasting the name of a community member who gets service at some month in the quarter to another month in a quarter to increase their number and the money the facility could get.

Though the sense of ownership of the leaders at the facility is good acting wrongly like this is illegal and a wrong perception towards CBHI. The other gap in the CBHI leadership is inconsistency and misses of board meetings. As a principle, the CBHI board should meet quarterly but most don’t do that and a few even don’t meet in a year. Concerning this even though the meetings are done regularly the meetings are not problem-solving. The problems we see this quarter continue to be a problem in the next quarter and beyond. These meetings show the presence of problems in decision-making and bringing solutions to the problems.

**Interviewer: Is there anything would you like to add?**

**KI:** Thank you, one issue I would like to raise is the fragmented pooling of premiums which is being solved and should be solved permanently. Previously sub-cities used to collect premiums separately and serve its community but now the premium is pooled to the city administration CBHI account. This has solved the problems of some sub-cities with a small number of members as they were suffering from deficiencies in the money which is now balanced by other sub-cities with better members. This is implemented in all sidama woredas and the pooling is now changing to zonal and regional pooling with new initiatives.

**KK12: Key informant interview 12**

**Interviewer: Could you tell us what you know about CBHI?**

**KI:** It is a system that helps the community to get a health service whenever they need based on a one-time monetary contribution they made for the year. You know previously a community member has to pay a lot for the health service and used to sell their assets to get the health service.

**Interviewer: Enrollment of the community especially women**

**KI:** Currently in our hospital more than 78% **of** the service utilizers are members of community-based health insurance. Of course, as CBHI includes the whole community it does not have a sex prettily. The CBHI normally recruits a family as a whole and women under that family are part of the CBHI. On the other hand in cases where women are household heads, they do become members themselves. Sometimes these women household heads need mobilization as women commonly don’t seek the CBHI service themselves. Otherwise, the CBHI has no system challenge in incorporating men or women as it recruits the whole house as a member.

**Interviewer: Is there a dropout from CBHI? Why?**

**KI:** Yes there is, some do not renew their membership. Those with chronic illness renew their membership constantly. On the other hand, a member who does not get ill and visits the facility for the whole year will tend to drop from the membership considering his initial payment as bankruptcy with no use. This is due to awareness problems and the awareness problem comes not only from the community members but also the level to which we in the health care system helped them in creating the awareness.

The CBHI scheme should inform the community when the registration and the money will be collected, what the importance of the money collection is, and its benefit. Otherwise, the community could not understand the CBHI system and its importance. To say that the woreda has reached some level of achievement in recruiting CBHI members the kebele leaders and others use different ways which are not even based on voluntarism.

Sometimes they add the CBHI payment to the tax or cut from the safety net program and receive from them other times they threaten the community members as they will close their shops uncles become members of the CBHI. Therefore generally there are no strategies for mobilization and enrollment of the community. Different persons use different ways which they think is efficient and this goes out of the voluntarism lane. What is on their mind is achieving the quota enrolment they are expected to achieve.

Low health-seeking behavior and low readiness of the health facilities are the other reasons for dropping out of the CBHI system. We need to work on the health-seeking behavior of the community through creating awareness by mentioning that even though they don’t get ill in the year their money is being used to help the neighbors and extended families in the community. We need to also to inform them that the money they are contributing to the CBHI is very low compared to the health service expense.

As I have mentioned earlier the other very important point is the readiness of the health facilities. Most of the facilities are not ready to the standard expected. The other reason for dropping out is what the community mobilizers have informed the community. Sometimes they inform them that once you have the CBHI book you are exempted from any payment for health service and will not pay anything. Which is wrong and is a reason for a dropout and will have a high expectation. We need to inform them they may pay for services that are not available at the facility. Sometimes they don’t even know the normal cost of the service and when they face paying for a single lab test or a drug that is not available at the public facility, they complain a lot. But if they have known the cost of the service, they already get from the public facility the minor costs they spent for the unavailable services would not be a big worry.

**Interview: Premium contribution and its management**

**KI:** When we think of the payment we need to see it from different angles. A service provider's payments do not get paid appropriately and on time. Second, when we provide service, we provide service to all users fairly whether they are CBHI members or not. The CBHI payment has been paid lately and the audit process is not transparent even they may not pay us all the money due to differences and disagreements in the audit. More mover due to poor efficiency of finance workers in the hospital the hospital lost money which had to be gained from the CBHI.

The health office initially pays 75% of the total payment from the CBHI in the quarter and will pay the rest 25% after the claim audit. But even before they pay us the rest 25% we run out of supplies like drugs and the money comes after months. When the money comes and you go to the market to purchase supplies like drugs, the money can’t buy what you have expected to buy due to the inflation as a result of the delay in the payment which we should have received a month ago. Therefore this delay of the payment to us in months and years we are suffering and the system has no interest system.

Those in the scheme sometimes lie that they have money in their account but they do not pay to the facilities. Because of this, it is difficult to get a facility that says they have received all the money they spent for the CBHI users. What would have been good is if the payments were given before the service. If the system is like that it will raise the availability of supplies and services in general. Otherwise, the payment will result in a cycle of problems.

**Interviewer: Are there adverse selection and moral hazards?**

**KI:** Yes, many CBHI members are utilizing beyond what they have contributed to the system. Because most of the members of the CBHI have a chronic disease. Therefore they utilize more than their yearly contribution in a single month intentionally or because they were ill. The other is the insurance itself is not profitable rather is in debut. The other important point because only poor and chronically ill people are members of the CBHI is the community’s perception that public hospitals are not well equipped and are for the poor. This is due to the historical perception of public hospitals as unclean, poorly supplied, and neglected places for the treatment of the poor as compared to private facilities.

But this perception is wrong these days public facilities have the best diagnostic machines, better supplies, and experienced human power compared to private facilities. What is wrong is the private has better promotion mechanisms than the public ones and the community believes his ear than his eyes. But sometimes the rich come to the hospitals, appreciate the service, and their mindset changes. Besides the rich shall know that the CBHI system is for shared benefits and could help others in a very limited yearly contribution to the system.

**Interviewer: What is the communities’ perceived benefit of CBHI?**

**KI: Now** the community has understood the benefit and they are utilizing the service. People see that a CBHI member is undergoing surgery and receiving many services without any payment and they start to understand the benefit of the system that way. However, the CBHI scheme is not effective in coordination. As you know the CBHI is not an independent company that selects public facilities based on its standards. It needs strategies like this. It needs to have its standards and select hospitals based on standards. But these days the scheme has even engaged in the wrong ways of achieving tasks of enrolling members like recruiting them out of the seated period of enrollment. Generally, right now it is the facilities who are leading the CBHI, not the CBHI who is leading the facilities in terms of the insurance as it gets in contact with any facility they get in nearby.

**Interviewer: What are the factors that you think can facilitate household enrollment and continued membership in community-based health insurance?**

**KI:** The first facilitator is the commitment and readiness from the government leadership is a very good opportunity. If this was not the case the system would have failed. The second opportunity is the policy and the system establishment is very good. What I think is a gap is the implementation and effectiveness of employees in the scheme. The other opportunity is the gradual improvement in the readiness of the facilities. Initiatives like system bottleneck-based reforms have increased patient services. More mover government’s initiative to bring highly standard machines to the public hospitals is a very good opportunity. The other opportunity is the public itself has started benefiting from these services and that has increased the communities' need to enroll in the system.

**Interviewer: What are the barriers to enrollment in community-based health insurance?**

**KI: Yes,** there are many challenges to the enrollment and utilization of the service from the CBHI. The first is the payment system. The payment system contributes to poor supplies of drugs and decreased quality of service. The community comes to the facilities to get services as they have paid their yearly contribution. But since the City Health offices didn’t pay us many supplies might not be available and sometimes the whole service to the CBHI users will not be available and the contract is cancelled. The other gap related to the payment is the claim audit.

The claim audit should be a system for correction not a means to subsidies the funds to the hospital. The bureaucracy of this payment is very challenging we would prefer not to be part of the CBHI contract. The suffering we have to get the money after providing the service to the community is unbearable. There are also gaps in the health information system in facilities that are needed for claim audits. However, the HIS for the claim audit in the CBHI cannot be corrected unless the whole HIS of the facility is improved as a nation.

The other is a wrong perception of the community that the CBHI users only suffer from additional payments and purchases of medications. This wrong perception as well as awareness gaps in the community as a whole is a barrier to the enrolment and utilization of the system.

**Interviewer: What strategies could be implemented to increase enrollment in community-based health insurance?**

**KI**: payment shall be pre-paid to the facilities. We cannot provide services to the community on debt because the budget itself will not allow us to do so. Therefore, the payment system needs to be changed completely. The other point is as the CBHI works with the public facilities these facilities need to be ready to deliver the service expected from them.

**Interviewer: Can you describe the organizational structure of the CBHI in your community and its effect on function?**

**KI**: The structure is not right but is good as per the current situation. As a structure, it should have been an independent institution just like other insurances and be out of the health care delivery system which recruits health facilities based on its standards and gets into contractual agreements. But as it is initiated in recent years the current structure of being under the political leadership’s direct support is the right measure. Otherwise, if it had started as an independent system and didn’t get political support it would not sustained. But I don’t think the government should let the structure be under the political leadership forever. The independence shall be piloted in some areas.

**Interviewer: What are your thoughts on the leadership of the CBHI?**

**KI**: The leadership from the political side is supporting the system but the scheme in some areas has poor efficiency and they are not fully engaged. They also have wrong perceptions like involuntary enrollments and others.

**Interviewer: Is there anything would you like to add?**

**KI**: Thank you I have nothing to add.

**KK13: Key informant interview 13**

**Interviewer**: **Could you tell us what you know about CBHI?**

**KI:** Well, thank you, community health insurance is an insurance system by which the community will pay once a year to be a member and get free health care services throughout the year without worrying whether he has money in his pocket or not.

**Interviewer: Enrollment of the community in CBHI**

**KI:** Well, it has been years since the community-based health insurance system was initiated in Ethiopia and the community has been involved in the system for years now. As the program is one of the government's flagship programs and a number of awareness creation sessions are being conducted the enrollment of the community into the system has increased. I believe this result in enrolment is due to the efforts from the health sector and other political leadership in different stakeholders.

Even though the enrollments have increased we also see communication issues that need correction. As the political leadership is becoming accountable for enrolling community members in the system and quota is assigned to them to enroll community members to the system they over-advocate the community members as the CBHI covers the expense for health services. But this is not right, the system so far does not cover the health services like dialysis. Therefore, such advocacies will be false promises that break the trust built between the community and advocators of the system and contribute to a drop out from the system.

The increase in the enrollment to the CBHI is also seen in an increased demand for medications at health facilities. Though there are additional reasons, one of the biggest reasons for the high consumption of drugs at health facilities is the increase in the number of community members who enroll in the CBHI and utilize the health care service.

**Interviewer:** **Are there adverse selection and moral hazards?**

**KI:** Mostly it is the poor who are enrolled in the system, and when members in the community see poor people getting free medical services two, three, four times a year with the money they could be paying for a card and very limited services in a private clinic the community appreciates the system and enrolls.

In my observation, the community member who has a better economic stance do not trust that the service given at public facilities has a good quality. To be frank, you know I am a civil servant, a health professional, and a leader in the health care system but I do not use public facilities I just recently started using a public facility. So most economically good people including government officials use private facilities. Therefore this is one of the reasons why there is adverse selection in CBHI users.

Associated with the moral hazard we commonly hear from health facility administrators that the day medications are purchased by the facility and stocked into the pharmacy the community will share the information among themselves and most of the CBHI users visit the health facilities in those first days.

**Interview: Premium contribution and its management**

**KI:** Ok, the government’s attention, political commitment, and continuous support to the Community health insurance is an opportunity in general but this political accountability on the other hand resulted in fear and burden among those who work on the CBHI resulting in false reports and poor data quality.

For example, the woreda might be expected to have or enroll 100 thousand and it is through advocating the insurance and convincing the community that they should enroll them. But in the problems, we see these days is a woreda report that all the 100 thousand is enrolled but those who pay to the CBHI will be 50,000.

Of course, the premium collection system itself is not right. Premium collection from the community has no common means across Woreda and is subject to fraud. The premium collection lacks transparency and is not digitalized at all. This has to be corrected otherwise data quality cannot be maintained and reporting false numbers and leakage of the collected many at different stages may persist.

The false report and poor premium collection have resulted in financial problems and bankruptcy in the health facilities. This financial problem does not stop there, the health facilities come to EPSA and take drugs on loan. The health facilities are supposed to sell drugs they take with a 25% profit. For example, if a health facility takes a 1-million-birr drug in loan it is expected to get 250 thousand profits from it and return the 1 million birr to EPSA. But this is not what happens. What is happening is the facilities will give the drugs to the CBHI users for free but they will not be rewarded with the money from woredas or region for the drugs they dispense for the CBHI users because of different reasons like the one I raised as a false report, leakage of money or other reasons. Therefore because of these reasons, the health facilities do not pay their loan money to us the EPSA. For example, currently, our hub has over 165 million birr loaned and not collected from the health facilities in the area. Nationally EPSA has loaned over 1.7 billion birr to health facilities and it is not yet returned.

The facilities in turn couldn’t buy a drug as they had no money to buy drugs. This affects the quality of the service the facilities provide as they will have limited laboratory investigations to carry out and drugs to dispense. I think we all need to work on this issue and solve the problem in premium management for the community to enroll and get the service they expect from community-based health insurance.

**Interviewer**: **What is the communities’ perceived benefit of CBHI?**

**KI:** Yes, though there are awareness gaps among the community, the community is now understanding the benefit of the system. The main reason for the community to understand the benefits is learning from others who benefited from the system. You know they will see people who are poor and do not have the money to get the service, but they get a free service due to the insurance and are cured of their illnesses. This helps everyone in the community to understand the benefits.

**Interviewer**: **What are the factors that you think can facilitate household enrollment and continued membership in community-based health insurance?**

**KI:** One of the opportunities is the government’s attention and support. The political leadership has shown a strong commitment and focus to this system and it is appreciable. These days it is a political agenda and since politics is the center everyone at each administrative level is contributing to the system. You know health insurance is a means to support each other, as many pay and a few needy use the service which they could not have gotten if there was this system. This goes with the Ethiopian culture of supporting each other.

As I have mentioned earlier noticing exemplary individuals from neighbors who benefit from the CBHI is another reason and an opportunity for others in the community to enroll into the system.

The other opportunity is an increase in the number of health facilities in the community. You know these days there are health centers and hospitals nearby the community, therefore the community will not have access issues to enroll in the CBHI. They can enroll in the CBHI and get the service at their nearby facilities which are easily accessible.

The other opportunity is the presence of a health insurance policy and guidelines in the country. It also has an organizational structure from the federal level to the bottom which facilitates to easily accomplish tasks across the levels and engage the community in the system.

**Interviewer**: **What are the barriers to enrollment in community-based health insurance?**

**KI**: You know the health service to be provided to the CBHI users includes laboratory and pharmacy services. But recently drug supply has become a challenge to the health care system and is becoming a challenge to the CBHI too because the users do not get the prescribed medications from pharmacies in the public institutions buy them from private pharmacies frequently. Of course, there is a general shortage in drugs nationally because we import more than ninety percent of the drugs, need the support of many stakeholders including the shipment, facilities do not properly quantify the drugs they know, and there is a foreign currency shortage for purchasing.

As EPSA is not a profit company and the money in EPSA works in a revolving drug fund we get challenged to buy drugs due to a delay in the health facilities to pay their debts. Therefore, we started not to loan drugs to health facilities, which in turn resulted shortage of drugs at health facilities and a financial burden on the CBHI users.

This results from a deter in the membership to the system and suffering among the community. This also results in access problems as laboratory investigations or drugs the clients need may be expensive and sometimes unavailable in private facilities in some woredas with a final negative outcome of morbidity and mortality. This when summed is significantly affecting membership to the CBHI.

The false reports in the number of people enrolled, data quality issues, and false promises especially among the lower political leadership due to fear of accountability which I have raised earlier is a problem for the management of the CBHI system which in turn results in poor financial management and planning, dissatisfaction in the service, and drop out from the system.

Poor quality of service which is not as per the standard is the other barrier for the community to enroll in the system. The supplies including laboratory reagents and drugs shall be available for the health service users unless it will continue to be a barrier for the community member to enroll in the system and rust public health facilities.

**Interviewer**: **What strategies could be implemented to increase enrollment in community-based health insurance?**

**KI**: One of the strategies we could apply which has been started in some areas is engaging institutions, companies, and people with a better income in disposing of their social responsibility by contributing to the CBHI premium. For example, our institution has covered the CBHI payment of 100 community members for this year. I have also paid the CBHI premium for 5 community members who could not afford to pay it. So through this way we could the community members to enroll to the CBHI and also help the system financially.

Moreover, the financial management system should be improved. Starting from the premium collection it should be digitalized, needs to have a similar framework of collection in every corner of the country, and needs strong management support.

More than this what I think needs to be emphasized is improving the service quality at public health facilities. If a facility is opened as a health center, then it should provide services expected from its level. If there should be a laboratory service then there should be a laboratory service. If medications have to be there then we need to avail those drugs. Right now, there is good access to health facilities in terms of number but we need to reform the quality of service and provide the supplies needed to the facilities.

**Interviewer: Can you describe the organizational structure of the CBHI in your community and its effect on function?**

**KI**: Ok, when we see our health system there are three pillars: the one who provides service mainly the health facilities, the second is the one who receives service including the health insurance service and the third is the controlling body mainly the food and drug authority (FDA).

The problem in our structure is the health service received (The health insurance service) which is the managing structure of health insurance users under the health service providing body. This is a mix in the structure. There is even a gap in structural communication between the regional and federal and regional health insurance structures. For example, here in Hawassa, there is the federal CBHI structures branch office and then there is the Sidama region CBHI regional body. The Sidama regional CBHI is under the medical services unity of the regional health bureau which is led by the deputy health bureau official of the region. You see this medical services unit is the health service provider for the general community including the CBHI users but also it is this unit that runs and controls every activity of the CBHI in the region. This makes the data quality issues I have raised earlier false reporting very prone, and accountability very ambiguous.

Concerning the above point the political leadership needs to have key performance indicators to check and balance to check discrepancies in data between the federal and regional structures and verify data from woreda and other administrative levels. We need to see what is on the ground, we need strong monitoring and evaluation meanness.

For example, X Referral Hospital has a 14-million-birr debt from EPSA. When we requested the hospital to pay the debt, they responded that they couldn’t get the money for the money service they provide service to CBHI users from the health bureau. If the bureau had enrolled all this much community who pay to the CBHI why would it be a problem to pay for the service the users utilized, this means some users are members of the system without a contribution, this needs monitoring. However, the monitoring and evaluation are complicated because the provider and the one who receives the service are embedded under the same structure.

This problem may get complicated when we include civil servants in the CBHI. As the civil servant is a community with better awareness, it will ask if we can’t provide the service we promised. Therefore the structure needs to be reformed before we add another part of the community to the service.

**Interviewer: What are your thoughts on the leadership of the CBHI?**

**KI:** The leadership’s commitment is appreciable. But the leadership shouldn’t only focus on enrolling the community rather they should also monitor the quality of service provided at the facilities and try solving the bottlenecks that cause dissatisfaction among the CBHI users. Otherwise, prolonged problems can backfire on the service provider and the government. **Interviewer: Is there anything would you like to add?**

**KI:** We have raised a lot of points. The initiation is good and appreciable. But when we enroll the civil servants can create more challenges. Therefore, all stakeholders and political leaders shall work on its overall improvement beyond enrolling the community in the CBHI system.

**KK14: Key informant interview 14**

**Interviewer**: **Could you tell us what you know about CBHI?**

**KI:** CBHI is a system by which the community contributes a limited amount of money to the system in a year and receives health care services for free throughout the year.

**Interviewer: Enrollment of the community in CBHI**

**KI: previously there** was a problem engaging the community in the system as per the plan. We plan to make at least 85% of households in the community a member of the CBHI. During the first years, the awareness in the community was low and the enrollment was not as planned. The voluntarism to join the system was low and the community would join due to the pressure from the coordinators of the system. Though there are improvements still the community’s imitation to join the system is not as needed. Some know the benefits of the system and enroll but most still do not need to join the CBHI.

The other important point related to enrollment is the period for enrollment, which is not at the right time for the community. The period of enrollment here is after the time at which the community sells its cash crops and spends its money. It would have been good if the enrollment period was adjusted to the time at which most of the community members access money. Additionally, as the principal people pay in a known specified period and receive the service from that time onwards for one year. But in our setup, the enrollment period is not fixed and people sometimes get enrolled and a different period and would like to receive service as per their payment schedule.

**Interviewer**: **women's involvement in CBHI**

**KI:** Women’s participation and enrollment are far better than men's because women in our community do not have the power to decide on the house money and properties, they choose to be a member of the CBHI and get the health care service for free without any worries. However, the enrollment of both women and the general community is low and needs additional efforts.

**Interview: Premium contribution and its management**

**KI: As** I have mentioned earlier the enrollment period is not at the time on which the community has better access to the money. As the principle, people pay in a known specified period and receive the service from that time onwards for one year. But in our setup, the enrollment period is not fixed and people sometimes get enrolled and a different period and would like to receive service as per their payment schedule. This creates a mess in schedules and creates a complaint among the community. This shows a gap in cash collection. Of course, this problem is getting solved this year as the cash collectors are changed from kebele leaders to HEWs.

The other issue related to payment is the payment system from the city administration to health facilities. We have a contractual agreement with the city administration stating that payment should be executed to the health facility within 10 days of the request of the facility. But this has never been done. What is happening is we request for the money quarterly and we expect them to pay us the 75% before the claim audit but it has been several months since we receive the fund.

For example, it has been two weeks since we ask this quarter’s payment but there has been no response from the city administration health office. In our previous experiences, it is after a month of notifying the CBHI board chairman that the payments get paid. This is making a budget shortage and purchasing drugs and supplies a challenge for the institution and when we stop the service to the CBHI users due to this, it is commonly politicized and forced to resume the service.

**Interviewer:** **Are there adverse selection and moral hazards?**

**KI:** Yeah, I have such an experience with a moral hazard. Once while is was working in OPD a patient I had treated five days ago came back and asked for a drug as if there were relapses in the disease. Then I communicated with other colleagues working in other OPDs if they knew the person and had a similar experience and they had encountered the same experience from that individual.

Then we notify the facility head and go to the client’s home to see what he is doing with the medication. We couldn’t find the medication at home and we have found out that he sells the medication and uses this act as a source of income. Such acts are common in rural areas because they find the money from selling medications as big money.

In terms of adverse selection yes there is. Normally we want all except the civil servants to be a member of the CBHI. However, the rich are not commonly a member of the CBHI because they mostly don’t trust the service given at public facilities and prefer private health care providers. Sometimes they also think that they have money and don’t want the insurance. This all is due to problems in awareness as they think that CBHI is for the poor.

**Interviewer**: **What is the communities’ perceived benefit of CBHI?**

**KI:** Yes, those community members who heard that someone got a free health care service for surgery or other health care service which would have needed a lot of money understand the benefit. On the other hand, those who don’t get ill in a year respond why will I pay for the CBHI thanks to GOD I am fine. Others replied when asked to enroll in the CBHI ‘There are no drugs at the facilities why will I be a member’; A few others also replied ‘Drugs are only given to those who pay in cash. The CBHI users do not get drugs. These replies in general show awareness gaps and failure in understanding the benefit and general system of CBHI.

**Interviewer**: **What are the factors that you think can facilitate household enrollment and continued membership in community-based health insurance?**

**KI:** The community at the urban is far better than the rural one as they have a better awareness. The city administration itself helps the poor to be a member of the CBHI. Not only the government but also the rich in the community have also helped the poor members of the community to be members of the CBHI. The other opportunity for the enrollment of the community is the effectiveness of the service given at the health facilities which has improved gradually.

**Interviewer**: **What are the barriers to enrollment in community-based health insurance?**

**KI**: First, those members come to receive fair and efficient service. However, there are gaps in the health facilities like unprofessional acceptance of the clients and shortage of supplies like drugs or laboratory services. The other is an awareness gap where they think they will get everything they want for free at the facility but the facility may have a shortage in supplies and they don’t understand this. Commonly once an individual doesn’t get the drug, he wants in the facility he may never come to the facility thinking that there is no drug in the facility.

**Interviewer**: **What strategies could be implemented to increase enrollment in community-based health insurance?**

**KI**: As a nation, there should be a standardized seated date for enrollment to the CBHI. After they enroll in the CBHI they have a right to access the service but we sometimes see clients persuading the professionals to write drugs they want and this should be corrected. The facilities should provide standardized equitable services to the clients. Moreover, the facilities shall do their best to avail all services and drugs. To make this happen the payment system should be improved and pay the funds of CBHI to the health facilities. If we do this the community will engage with the CBHI.

**Interviewer: Can you describe the organizational structure of the CBHI in your community and its effect on function?**

**KI:** I don’t have detailed information on this. But for the CBHI services, we communicate with the city health office. The service at the top is led by the CBHI board and there is the CBHI scheme. But the structure seems confusing the scheme coordinating the CBHI says we are an independent office at other times they respond as if they are under the city health office. We frequently communicate with the city health office as he is a member of the board and the scheme coordinator.

**Interviewer: What are your thoughts on the leadership of the CBHI?**

**KI:** The CBHI is a political agenda. It is led by the regional prosperity lead and the zonal leads. The commitment by the leadership from the region, and zone to the woreda is good as it is one of the indicators of monitoring for them. We have also those who are arrested for the failure of financial accountability in CBHI.

**Interviewer: Is there anything would you like to add?**

**KI:** Generally, the CBHI is a system that supports all members of the community member but our communities’ awareness is low and it would be nice if they enroll voluntarily. They shall come and see the rumors about the CBHI are wrong and everyone gets fair and equitable service. Other than this the financial audits shall be transparent and facilities need to be supported in budget.

**KK15: Key informant interview 15**

**Interviewer**: **Could you tell us what you know about CBHI?**

**KI:** Ok, thank you community-based health insurance is one of the mechanisms in the health care system that creates a fair and participatory health care delivery for the population. It allows all aspects of the population to get a health service freely on the basis of their initial enrollment into the system. The system is started in 2010 in our country. In our town, it started in 2010 as one of the pilot areas in the country.

**Interviewer: Enrollment of the community in CBHI**

**KI:** As I have stated earlier CBHI was initiated at our city in 2010. However, the intention to involve and involvement of the community is high as compared to other woredas. In terms of number 8148 members are enrolled this year in the city. Among these more than 40% are women household leaders. In terms of utilization of the service even though there is no a study conducted and commonly encounter women utilizing the service.

**Interviewer**: **women's involvement in CBHI**

**KI:**  Good**,** of course, pregnancy, birth, and immunization are not under the CBHI. However, the women and children benefit from the CBHI. Even though we do not have specific data on the number of women at hand a large number of women and children visit the health facilities in our city using the CBHI system.

**Interviewer: Is there a dropout from CBHI? Why?**

**KI:** Yes, there are dropouts, even though we don’t know the exact number/. One of their reasons is a lack of awareness. The other is poor service delivery from the health institution and dissatisfaction from the community. You know there can be problems in the facilities from professional ethics to shortage of supplies or other perspectives. Though everyone does not get ill and go to the facilities some may go and utilize the service we hear that they get dissatisfied with the service they received and they then share this bad experience with other members of the CBHI in the community. This way these individuals as well as others who are influenced by this information drop out of the service.

What we are fearing even for the next year as a challenge in our setup is as you know as an administration we have a contract with our hospital. Based on the contacts payments will be made to the hospital but some drugs are frequently unavailable at the hospital. Additionally, the hospitals refrain from providing the service for the CBHI user during the few financial processing days. For example, today the service for the CBHI user at our hospital has stopped a two-day delay in the financial process of the payment to the hospital. Factors like this contribute to the dropout of community members from CBHI.

The reason for the financial delay is there was a backlog of payments for the hospital in the last fiscal year. But we paid more than 4 million last year and we paid two-quarters payment of this year. What they have done is they requested the third quarter payment with a letter and immediately stopped providing the service. This type of approach affects the communities trust and involvement to the system. I think if all the community member receives good service and our facilities are well-equipped, we can decrease the dropout.

**Interviewer:** **Are there adverse selection and moral hazards?**

**KI:** I think there are awareness problems. People mainly enroll in the CBHI when they encounter a health problem, especially a chronic disease. For instance, in our city, more than 600 are chronically ill patients. This person actively involved in the CBHI system renews their book without external pressure. Similarly, those who are poor and get government support actively engage in the CBHI system pay their payment on time, and renew their books. Otherwise, it is those with an accident or in an emergency are the common members of the CBHI.

These chronically ill and poor people put pressure on the healthcare system. People are allowed to be a member of the CBHI at the specified time of registration otherwise one cannot be a member at any time they want. The other people are not involved and need pressure because of awareness problems. This should not be under pressure rather they should take it as an advantage for their health, they will pay 650 birr per year and will be insured for their healthcare needs.

**Interview: Premium contribution and its management**

**KI:** Very good. Payment for the CBHI is done at the start of the Ethiopian calendar year each year through community mobilization and sector initiatives in each woreda. Then members will be enrolled. There are a lot of challenges in this period. The first is since the community does not get to the system voluntarily the political system from the woreda to the kebele should conduct a community mobilization which is tiresome and sometimes not voluntary.

After the mobilization finance receipts will be will be given to the kebele leaders or HEWs. In our city, it was kebele leaders who took the finance receipts but this year for better facilitation it is HEWs who will take the finance receipts responsibly. This change in the responsibility of finance receipts from kebele leaders to HEWs has made us advantageous as the HEWs are under our command, we have controlled every process and accountability has been maintained.

When the HEWs enroll community members in the CBHI they are oriented to accomplish their task with care as some community members come with different justifications to pay half of the one-time payment and pay the rest at another time. But we do not accept such payments rather the HEWs work as per the CBHI guideline. So, they receive the money from each member and give them the receipt, prepare the CBHI book for each member, and deliver the book to each member.

So, the enrollment and payment system are tiresome and difficult to manage in a few individuals. But since this whole process is being led by all leadership structures in the woreda we are successful. After the money is collected from the community in this way it will be deposited to the CBHI account in the bank and a back slip will be submitted to the city health office finance.

The other important thing in the premium and its management is the payment system to health facilities. In the last year, there was a backlog to be paid to health facilities. In last year's audit, we found that there was a backlog to be paid to our hospital from the 2012 to 2015 fiscal year. Therefore, with the support of the city mayor, we have tried to cover this debt. This year we paid 4 million to our hospital including the debt including the first two quarters of the 2016 fiscal year. Now they have requested the payment of the third quarter and the letter for the payment is ready. 75% of the quarter’s payment will be paid now and the rest 25% will be paid to the hospital after the claiming audit.

**Interviewer**: **What is the communities’ perceived benefit of CBHI?**

**KI: Of course,** those who know the benefit get enrolled and renew their membership without pressure. As a community, we cannot say that the community has understood the benefits of the system. If they had understood the system's benefit they would come and be a member without pressure as in the case of the Western world. Of course, a large number of the community is benefiting from the system and they clearly understand the benefit.

They confess the benefit of the CBHI in different stages of meetings. They mention what and how they have benefited from the system everywhere especially those who get the service at difficult times like surgeries traumas and others. On the contrary, there are a few members of the community who do not understand and feel that it is important.

The first ones are those who are not members of the CBHI. The second are those who are members but were not ill and did not visit the health facility in the year. This is the second people feel as if they have wasted their money. The third is those who received a service but were not satisfied with the service. These all can be corrected with awareness creation and promotion by those who received the service. But still, some people thank us as if they were treated by our effort not their money. Therefore, I think most of the community understands the benefit of the system and it will be improved if awareness creation and quality of health service is improved.

Moreover, awareness creation should be conducted well. Mostly awareness creations are not conducted thinking that the community knows it. We need to have health professionals who understand the CBHI and its benefits well to create awareness in the community. The awareness creation should be with information and examples. The other way is testimony of those who benefit from the service would be the best way of creating awareness.

Sometimes community members believe and have rumors that there are unfair treatment among the cash payers and the CBHI users but it I wrong. I have helped my father get treated in black lion hospital with the CBHI. The system is very important and benefits the community. A lot of community members benefit from this service. Even there is a patient who is admitted at HUCSH from our city through the CBHI system. We can use examples like this to promote CBHI and create awareness.

Most of the community members are happy with the CBHI but they have disappointments with a shortage of drugs or unethical professionals. As an example, there was a man who had been diagnosed with BPH who had been seen at our hospital by doctors, got different investigations, was referred to HUCSH, was admitted for 30 days, and a surgery with CBHI.

But this person bought a 4000 birr medication at his discharge and because of this payment the individual used to spread misinformation as if CBHI has no importance. But I have encountered this individual and persuaded him of the benefit by comparing what he has got from the whole treatment which would be a 20,000-birr service and the 4000 birr he paid. Therefore, we need strong awareness creation sessions.

**Interviewer**: **What are the factors that you think can facilitate household enrollment and continued membership in community-based health insurance?**

**KI:** Political engagement is a very good opportunity for the communities I enrolled in the CBHI. Political engagement from mobilizing the community to awareness creation, facilitating health care support, and financial support is a good facilitator for the engagement, mostly husbands are not out of the wife’s thoughts. Therefore, the husband’s good perception of the system is a facilitator for the CBHI. The other facilitator is the presence of cash commodities like coffee and, cash among the community. This is a facilitator for the community so far and we can leverage this opportunity

**Interviewer**: **What are the barriers to enrollment in community-based health insurance?**

**KI**: One is we don’t plan for health. This is the biggest barrier our community does not have a saving habit in general and specifically for health, The community sells all it has for the coverage of expenses to receive health care service. Therefore, awareness created mainly among women could solve this and I think we need to push on that.

The other barrier in our community is the timing of the enrollment period for the CBHI. Even though it needs a decision from higher bodies I think the community could enroll more if the enrolment period be changed from November to February, to August to December this is the period at which the community will have cash at hand from its cash crops.

The other barrier is poor community mobilization in some areas. The community will not get enrolled unless there is a good community mobilization and it needs to be improved. Additionally, those I raised earlier like poor health care delivery system and dissatisfaction of the community from the service they received due to unethical professionals and poor supply of drugs a big barrier in the CBHI system.

**Interviewer**: **What strategies could be implemented to increase enrollment in community-based health insurance?**

**KI**: we need strong community mobilization and awareness creation sessions. this can be done with awareness creation and promotion sessions by those who received the service. The awareness creation should be with information and examples. The other way is testimony of those who benefit from the service would be the best way of creating awareness. The other point of improvement is strengthening and supporting the health facilities with supplies like drugs. If we cannot do that and even support them financially the communities’ dissatisfaction cannot be addressed. The payment collection shall be taken away from HEWs and would be nice if agents like the one being done for electric service would be a good option and reduce the burden of HEWs

**Interviewer: Can you describe the organizational structure of the CBHI in your community and its effect on function?**

**KI:** CBHI is led by a board. The board chairperson is the mayor and members are the health office. Women and children office. Culture and tourism office, and the finance office. The CBHI scheme coordinator is the secretary/ The CBHI scheme has three employees which include the coordinator, finance expert, and data clerk. This scheme is under the health office of the city administration. This scheme members work with the HEWs for enrollment and payment collection from members.

The structure does not go to the community level and has human resource problems. The CBHI’S tasks. Everything including the community mobilization is budgeted. But I think the insurance shall standalone from the health system nationally even though we cannot predict its success as it is standing now with the support of the political leadership for the future it should function independently. Now it is under the health office.

**Interviewer: What are your thoughts on the leadership of the CBHI?**

**KI:** There is great leadership in everything from enrollment to budgeting it is led by the mayor of the city and I think this has played a very important role in the success of the system. There is continuous monitoring by the mayor, the board, and the health office. Therefore, I think the system is functioning till this time due to strong leadership support from the regional leaders, zone, and woreda to kebele.

**Interviewer: Is there anything would you like to add?**

**KI:** Not much. As CBHI is a very important system for the community we need to address those barriers on the ground.

**KK16: Key informant interview 16**

**I: thank you for your participation. I am RA from Hawassa University. This research is about community-based health insurance. As you are the leader of this kebele I was hoping to gate information about CBHI coverage at this kebeles.**

**What you can tell me about CBHI starting from when it started**

R: thank you. Community based health insurance started in 2003 E.C. I have been a member since the beginning. The program started in 2 Woreda namely in Yirgalem and Wolayita Demboya when it was under SNNPR. I know well about the use of CBHI since I was a board member during the initiation and also a user of CBHI. At the start, not many people knew about the advantages of CBHI but currently, there are many users.

**I: what is the CBHI coverage in this *kebele*?**

R: this year we have around 690 members of CBHI. Previously we had to visit home to home to collect payments but Nowadays people know well about the advantages of CBHI people willingly pay without being asked. As I have told you the program started in two kebeles now it is implemented in all Woreda and the number of members has increased. There are some community members like government employees who are not members of CBHI otherwise a larger portion of the community is a member. The cost increased this year to 600 birr for those already enrolled and 650 for new members, which has caused some complaints from the users but still, they are well informed about the use so they continue their membership.

**I: is there any dropout (discontinuation of CBHI membership)**

R: the dropout rate is not much; last year 29 members discontinued their membership. The main reasons are the change in residence and due to a rise in the payment, some members don’t have the economic capacity to pay that much. To tell you from my experience my wife is diabetic she refills her medication using CBHI for the whole year, if we try to buy the medication without insurance it is very expensive. This is not only my experience but of many. There are many people with chronic diseases like hypertension heart cases and DM. so most people are advantaged and don’t think of leaving the program.

**I: my next question is on how the members use CBHI. Do the members use their membership appropriately?**

R: yes, the member uses their insurance appropriately. Previously it was difficult to get medication in health centers and hospitals. But now the medications are available and people are using their insurance appropriately. Some medications are not available in the health centers for example if a patient is prescribed 4 medications, he/she might not be able to get 1 out of 4 medications (not due to a problem in CBHI, but supply of the medication).

**I: as you have told me there is proper use of CBHI because you provide adequate service. What about in regards to users; have you ever encountered inappropriate use of service by members just because they are insured?**

R: no, we have not encountered this kind of problem. members use their insurance appropriately to seek treatment for their illnesses.

**I: which members of the community are most users of CBHI?**

R: the majority of the users are community members with low socio-economic status. People who have the economic capacity can seek treatment anywhere they choose. Most users are those who have been struggling to get treatment.

**I: what do you think is the reasoning behind people with good socio-economic status not using CBHI?**

R: those communities are also members of CBHI; they pay their payments accordingly and timely. They sometimes also use their insurance. But when compared the majority of users of the insurance are those with low economic status. The reason might be that people with good economic status can seek health services anywhere they want. We also support community members who cannot pay for the insurance. Currently, there are around 190 members who are supported by the kebeles and city administration who can get health services without any payment.

**I: what is CBHI coverage in regards to women?**

R: most of the members are women. We motivate women to be members and they are high users of the service.

**I: what is the communities’ perception about benefits of CBHI?**

R: it is good! It is very good! The community has a very good knowledge of the benefits of CBHI. As I have told you before if a person has money he/she can get health care anywhere they want. The poor community can’t afford medication since it is very expensive. But by being a member they can afford medication. They are even very willing to pay in time so they are highly well-informed of the benefits. The health center gives health information at each, kebeles by setting meetings with community members also city administration and kebeles officials participate in providing information and training to the community.

**I: how does the community perceive the benefit of CBHI specifically for women**

R: women are highly beneficent and the community also understands the importance of CBHI for women. Women can access health care without direct payment.

**I: what benefits does CBHI provide to women?**

R: - some women were not able to get healthcare due to financial issue. There are even some who died because of this. Now that there is CBHI they seek health care early stage of the disease before it complicates. So there is a high benefit to women in accessing health care. They even say this program is especially useful for us (the women).

**I: you have previously mentioned that government employees are not members of CBHI what is the reason?**

R: this is not because of us but due to the government’s decision not to include the government in CBHI. The program was started by excluding gov’t employees. People who are on retirement pensions are also not included in CBHI there have been questions raised by these community members and we have already reported this to regional administrators. They have told us they are working on finding solutions which will be implemented shortly.

**I: what strategies do you think can be done to increase communities’ use of CBHI?**

R: the first thing is allowing all members of the community to be members of CBHI. As I have told you government employees cannot be included. This should be improved; they face difficulties in getting healthcare and medication because they can’t afford it.

They even made suggestions to be included in paying more than what other community members do. The other thing is regarding the time of payment. In the current trend payment from the members is collected in Tikemt. This has caused some problems, when people seek health at the start of the year they are told the insurance could not cover their costs because payment was not made. So the money from the community should be collected at the start of the year (Meskerem).

**I: could you tell me about the payment system**

R: After the members receive health service the bills will be collected by health extension workers. Then payment will be made to the health facilities after auditing.

**I: what could be done to improve the enrolment of women especially mothers in CBHI**

**R:** most mothers in this kebeles are members. However, giving training and information about CBHI benefits early can be helpful.

**I: How is CBHI organized?**

R: there are 9 villages in the *kebeles*. We conduct meetings at each village and give information to community members about CBHI. We also work with religious sectors and Idirs to disseminate information. Additionally, there are 3 health extension workers residing in the *kebele* that give training to mothers.

**I: how can leadership affect CBHI implementation?**

R: good leadership can facilitate the implementation of CBHI they gave their time and effort for the success. We have not faced any problems regarding leadership.

**I: finally, is there anything you want to add**

R: not many, I want to add that there should be more work done to improve the supply of some medication

**I: thank you for your time.**

R: thank you.

**KK17: Key informant interview 17**

**Key: -**

**M**: Moderator

**KI**: Key Informant

**M: Could you tell me what you know about CBHI?**

**KI:** I believe CBHI is a life guarantee and insurance for health services. It creates the opportunity for cooperation and support among the community. It has great benefit to the people.

At the beginning time, there was an awareness problem among the community. They said, “how do we save money predicting that I will become ill? This is not a good plan to be ill”. Still now this type of thinking is not completely mitigated. We expect 100% engagement after the awareness problem is solved.

**M: How about CBHI enrollment?**

**KI**: We achieved 82% coverage in the city. By now the share is 650 ETB per household. For this achievement, government support has a significant impact.

**M: What about dropout status?**

**KI**: Until the awareness problem is solved, dropout is expected. Contributors consider themselves as not benefited if not treated in the contribution year. Supporters dropped out because of the phase-out of projects. Previously the premium amount was 400 ETB but now increased to 650 ETB which could impose negative feelings among members.

**M: Premium and its management?**

**KI**: Money was collected by the financial system of the government. It is also auditable. We have a transparent system to expend the collected money under the control of the mayor of the city administration. We reimburse health facilities when they request based on the audit of the provided service.

**M: Moral hazard (the tendency of insured individuals to overconsume healthcare services because the costs are covered by the CBHI insurance)**

**KI**: There is minimized reflection regarding this, but there is limited tendency.

**M: Adverse selection (high-risk individuals being more likely to enroll, leading to higher costs and premiums for the CBHI pool)**

KI: Poor households who couldn’t afford the share got membership for free. They are 1992 households benefited from this opportunity.

**M: How about specifics related to women?**

**KI**: We encourage all community members except government employees. So, we are committed to engaging more women. Our plan also shows more numbers of women.

**M: How does the community perceive the benefits of community-based health insurance?**

**KI**: They perceive that CBHI is very useful for them. We used different structures to mobilize the community to be engaged.

**M: What is the community’s perception of CBHI, specifically concerning women?**

**KI**: All community members prioritize women to get health services because anybody has good awareness about the vulnerability of women to different health problems.

**M: What are the factors that you think can facilitate household enrollment and continued membership in community-based health insurance?**

**KI**: Medicines and investigations availability with professionals' motivation and awareness to serve CBHI beneficiaries equally with customers who pay out of their pocket.

**M: What about among women of childbearing age?**

**KI**: Service availability is very important. All women need timely and accessible healthcare services.

**M: What are the barriers to enrollment in community-based health insurance?**

**KI**: Stock out of medicines and interruption of some medical services could affect the engagement. There shall be maintained medical supplies availability.

**M: What about among women of childbearing age?**

**KI**: If medical services are absent, women are frustrated and hesitate to be members of CBHI.

**M: What strategies could be implemented to increase enrollment in community-based health insurance**?

**KI**: firstly, community mobilization is very important.

Second, trust building among community members.

Third, a specific plan is very important

Fourth, including all population segments to be engaged.

**M: Are there any strategies specific to women?**

**KI**: I think the same strategies to be used for women. May the be approach very good for women.

**M: Can you describe the organizational structure of the CBHI in your community?**

**KI**: It is not independently organized. There should be CBHI-specific focal persons in the health system. Transparency, responsibility, and accountability delegation are very important. There should be a clear controlling mechanism.

**M: How are decisions made within this structure? Who holds the most influence or power?**

**KI**: The most power is in the hands of the mayor to control the financial system. The technical part is the responsibility of the regional health bureau.

**M: How does the hierarchy within the CBHI affect its operations and effectiveness?**

**KI**: The existing hierarchy to manage the financial system is good. For me, it is effective to control money. But still, it needs a specific structure for technical support.

**M: Are there any challenges or benefits associated with this hierarchy?**

**KI**: The current hierarchy is effective in managing money, but cannot address the very technical part of the services given and missed to the members.

**M: What are your thoughts on the leadership of the CBHI?**

**KI**: I think local and regional government is committed to CBHI implementation and success. The health bureau should fully support the technical part of the implemented system.

**M: How does the leadership impact the functioning and success of the CBHI?**

**KI**: The leadership is very helpful for the current coverage achievement of the CBHI scheme. All stakeholders are supporting the implementation of the scheme.

**M: Is there anything would you like to add?**

**KI**: The government should work on the sustainability of the scheme through awareness creation among

Structural modification is needed. A scientific research-based decision is very helpful for the success of the scheme.

**M: Thank you very much!**

**KK18: Key informant interview 18**

Interviewer: Hello

**Interviewer: My first question is what do you know CBHI,**

KI: Please tell me about CBHI in the health center

**Interviewer: what do you know about it? About how many users there are and everything in general?**

KI: I don't know the exact number of users, but recently there has been a campaign to encourage everyone to join the CBHI by visiting households and raising awareness about different events. As a result, the majority of people are becoming members of the CBHI.

**Interviewer**: **Have you observed any instances of people discontinuing their CBHI membership? If so, why?**

KI: Yes, some people are quitting the program because of the lack of money. Others said, that they didn’t think they would get sick and stopped using it, but now they want to continue. However, the majority are still members. Some people neglect to continue, and in some cases, when a patient goes to the hospital and is prescribed four medications, one of the medications might not be available at the pharmacy. This causes frustration because they were told or believed that every medication would be readily available. We try to stock up on medications as much as possible, but sometimes when there's a shortage, people get discouraged from using CBHI.

**Interviewer: how is the payment process? And what impact did the payment issue have on the health center?**

KI: They pay well, although there are some issues with delayed payments. It's improving, but not completely solved. At the health center, the funds are used for various purposes. In the past, when payments were not going as planned, there were instances of budgetary constraints affecting operations, but the impact was not significant.

**Interviewer: okay. Who are the majority of users of CBHI?**

KI: CBHI includes everyone except for government employees. Despite their economic status, everyone interested can use CBHI. Anyone who can pay does so, although it's known that some struggle to make the payment. However, 650 Birr is not much to pay for those who can afford it. Therefore, anyone who wants to use it can do so.

**Interviewer: There could be part of the society that does not want to use CBHI, why do you think that is?**

KI: yes, some of them. It's well known that we don't plan to get ill, so most people only become conscious about their health when they get sick. In the past, when laboratory tests, medications, and other services cost only a few birr each, it was manageable. However, now healthcare is expensive. If a patient pays 600 Birr for Community Based Health Insurance, they will be covered for a whole year. This amount also covers the healthcare needs of the entire family for a year. People need to consider this before deciding not to use the CBHI. People who have good economic status might also not use CBHI and prefer to be treated in private facilities.

**Interviewer**: **Have you noticed any specific groups or individuals with higher enrollment rates? How does this impact the CBHI pool?**

KI: Yes, some people with chronic illnesses, such as those who take lifelong medications for conditions like hypertension, diabetes, and epilepsy, are familiar with the advantages and disadvantages of using them. As a result, most patients with chronic illnesses who have follow-up appointments here use CBHI, although they are few in number (people/cases with chronic disease); almost all of them use CBHI.

Most of the time, complex cases are not handled at the health center level. Inpatient services are referred to other medical centers. The primary services offered at the health center level are for acute febrile illness (AFI), pneumonia, and other infectious diseases. Therefore, patients diagnosed with these conditions avail the services provided at the health center.

**Interviewer:** **Have you ever noticed users utilizing CBHI inappropriately? Why do you think their reason is?**

KI: Many members visit the health center even when they feel slightly unwell. If the condition is not serious, they may receive advice, but some people want to get medication because they have paid for their insurance. However, it is not possible as the professionals follow protocols and it would not be ethical. Although we are uncertain, some individuals may use the prescribed medications for other purposes. In general, medications are administered based on the decision of the healthcare workers, not the patients. Many people prefer to request medications based solely on their symptoms, but physicians do not make decisions solely on that basis. They conduct physical and laboratory examinations to confirm the diagnosis before prescribing medications.

**Interviewer: As mentioned earlier, the majority of society uses CBHI, but those with more financial resources prefer private healthcare. Why do you think that is?**

KI: It's a matter of preference. For example, many people prefer to receive their medication through injections. They believe that by seeking treatment at private institutions, their preferences will be met. This preference extends to specific medical examinations, but the decision ultimately lies with the physician and may or may not be fulfilled. By seeking private healthcare, individuals believe they can access any service they want. However, it's important to note that private healthcare does not offer unique services; it simply caters to people's preferences.

**Interviewer**: **how does the community feel about CBHI?**

KI: It can be challenging to implement new strategies, including CBHI and other interventions. Over time, however, they will become more widely adopted as their importance is recognized in the community. While I can't say exactly how many people in our area are using CBHI, I believe that the majority of the people in our catchment area are enrolled in the program, as we tend to see them when they come to the health center.

**Interviewer**: **Specifics related to women: How does CBHI cater to the healthcare needs of women in your community?**

KI: In this culture, the majority of women are reliant on their husbands for various services, such as healthcare. They visit health facilities frequently not only for themselves but also for their families, including antenatal care follow-ups, childbirth, and their children's health check-ups. With the CBHI, they have the freedom to access these services without waiting for their husbands or other support, as the expenses are already covered. This allows them to seek care whenever they need it. Even though it’s important for everyone I think it’s very much important for women.

**Interviewer**: **How can we prevent discontinuation of membership and enroll new members?**

KI: There are meetings about health and related issues. In order to emphasize its importance and promote awareness, it is crucial to provide information at all times. I believe that raising awareness will encourage more people to become members of the CBHI.

Those who were already members know the importance of the program and its neglect that is causing them to discontinue. Some of them use the money intended for payment for other purposes. However, for everyday living, they should have given more attention to their health, so providing education on the importance of health and wellness could help them take better care of themselves on a daily basis."

**Interviewer**: **You previously mentioned that members discontinue due to a lack of medical supplies. Can you explain more**?

KI: mmm it’s been two years since I’ve started here and I can say that there are good enough supplies of medications. Some patients get prescribed 3 or 4 medications and some medications might not be available even in private facilities. The medication in the health center setup is bought in the program so there might be medication stock out. As I mentioned those medications might not also be available in the areas outside the center so there is nothing you can do at such times. Efforts are being made every day but it's not a shortage issue (not due to the health center but supply by the government).

**Interviewer: Is it a budget issue?**

KI: No, we stack up the essential medications since they are a priority. What I love about the system is that you don’t just buy a medication whenever you run out or something, it's bought by analyzing what each department needs. They submit their list and by comparing that with medications at hand, that’s when medications are bought. We try to buy every crucial medication.

**Interviewer: Another reason you mentioned dropout is financial issues, how do you think we can solve that?**

KI: mmm that one cannot be solved by the health center solely, the majority of the time there are arrangements for the so-called poorest of the poor. The finance teams try to understand the situation, by identifying them and setting ways to help them. it’s just that this issue is bigger than the health center. Participating stakeholders is crucial for such issues. There was this organization (NGO) that helped this kind of people.

**Interviewer: What were the areas of work of the organization (the NGO)?**

KI: mmm Am not quite sure but people claimed that this organization paid for their CBHI. So maybe such ways can be set for other people as well.

**Interviewer: The next question is, what hindering issues would make women use CBHI in society?**

KI: It’s the government's priority to participate women but as I said before women might not participate in paying jobs. So, it's a financial issue. The other one is undermining them even though it's not common now and cultural suppressions (women shouldn’t participate in such matters).

**Interviewer**: **What can you say about the hierarchal arrangement of CBHI?**

R: it’s based on want Number of users/areas, member/kebeles

**Interviewer**: **who organizes this… membership, awareness… inclusiveness?**

KI: The whole CBHI isn’t something to be handled by a single body. For example, the HEWs reach out to the community, the health center workers give support, and awareness is given by Kebele leaders. Other community organs like religious institutions and women's forums are also involved. Since the majority use media nowadays media organizations also participate like FM radio.

**Interviewer: What contributions does the health center have to make everyone inclusive and others?**

KI: the health center staff contributes a lump sum of money and helps the communities with very low income. They also enrolled to set an example for the society.

**Interviewer: How is the payment circled to the health center once the member has utilized the services**

KI: Payment is audited and then reported even though am not sure with what frequency that is done since I am not in mandate but issues with payment are getting improved through time.

**Interviewer: CBHI leaders what is their impact/effect?**

KI: It’s to make consciousness happy, help those in priority, so they have a clear and clean conscience

**Interviewer**: **what do you think is the impact of lack of good leadership on CBHI?**

KI: First it affects members' perception of CBHI. Second, it would lead to many dropouts from the program. Plus, members wouldn’t get adequate and quality health care on time.

**Interviewer:** **I have finished from my side, is there anything you would like to raise?**

KI: Not much but I want to emphasize giving awareness to those not enrolled in the program.

**Interviewer**: **Thank you very much for your time**

KI: Thank you.

**KK19: Key informant interview 19**

**Interviewer**: **Could you tell us what you know about CBHI?**

**KI:** Well, thank you, community health insurance is an insurance system by which the community will pay once a year to be a member and get free health care services throughout the year without worrying whether he has money in his pocket or not.

**Interviewer: Enrollment of the community in CBHI**

**KI:** Well, it has been years since the community-based health insurance system was initiated in Ethiopia and the community has been involved in the system for years now. As the program is one of the government's flagship programs and a number of awareness creation sessions are being conducted the enrollment of the community into the system has increased. I believe this result in enrolment is due to the efforts from the health sector and other political leadership in different stakeholders.

Even though the enrollments have increased we also see communication issues that need correction. As the political leadership is becoming accountable for enrolling community members in the system and quota is assigned to them to enroll community members in the system, they over-advocate the community members as the CBHI covers the expense for health services. But this is not right, the system so far does not cover the health services like dialysis. Therefore, such advocacies will be false promises that break the trust built between the community and advocators of the system and contribute to a drop out from the system.

The increase in the enrollment to the CBHI is also seen in an increased demand for medications at health facilities. Though there are additional reasons, one of the biggest reasons for the high consumption of drugs at health facilities is the increase in the number of community members who enroll in the CBHI and utilize the health care service.

**Interviewer:** **Are there adverse selection and moral hazards?**

**KI:** Mostly it is the poor who are enrolled in the system, and when members in the community see poor people getting free medical services two, three, four times a year with the money they could be paying for a card and very limited services in a private clinic the community appreciates the system and enrolls.

In my observation, the community member who has a better economic stance do not trust that the service given at public facilities has a good quality. To be frank, you know I am a civil servant, a health professional, and a leader in the health care system but I do not use public facilities I just recently started using a public facility. So most economically good people including government officials use private facilities. Therefore this is one of the reasons why there is adverse selection in CBHI users.

Associated with the moral hazard we commonly hear from health facility administrators that the day medications are purchased by the facility and stocked into the pharmacy the community will share the information among themselves and most of the CBHI users visit the health facilities in those first days.

**Interview: Premium contribution and its management**

**KI:** Ok, the government’s attention, political commitment, and continuous support to the Community health insurance is an opportunity in general but this political accountability on the other hand resulted in fear and burden among those who work on the CBHI resulting in false reports and poor data quality.

For example, the woreda might be expected to have or enroll 100 thousand and it is through advocating the insurance and convincing the community that they should enroll them. But in the problems, we see these days is a woreda report that all the 100 thousand is enrolled but those who pay to the CBHI will be 50,000.

Of course, the premium collection system itself is not right. Premium collection from the community has no common means across Woreda and is subject to fraud. The premium collection lacks transparency and is not digitalized at all. This has to be corrected otherwise data quality cannot be maintained and reporting false numbers and leakage of the collected many at different stages may persist.

The false report and poor premium collection have resulted in financial problems and bankruptcy in the health facilities. This financial problem does not stop there, the health facilities come to EPSA and take drugs on loan. The health facilities are supposed to sell drugs they take with a 25% profit. For example, if a health facility takes a 1-million-birr drug in loan it is expected to get 250 thousand profits from it and return the 1 million birr to EPSA. But this is not what happens. What is happening is the facilities will give the drugs to the CBHI users for free but they will not be rewarded with the money from woredas or region for the drugs they dispense for the CBHI users because of different reasons like the one I raised as a false report, leakage of money or other reasons. Therefore because of these reasons, the health facilities do not pay their loan money to us the EPSA. For example, currently, our hub has over 165 million birr loaned and not collected from the health facilities in the area. Nationally EPSA has loaned over 1.7 billion birr to health facilities and it is not yet returned.

The facilities in turn couldn’t buy a drug as they had no money to buy drugs. This affects the quality of the service the facilities provide as they will have limited laboratory investigations to carry out and drugs to dispense. I think we all need to work on this issue and solve the problem in premium management for the community to enroll and get the service they expect from community-based health insurance.

**Interviewer**: **What is the communities’ perceived benefit of CBHI?**

**KI:** Yes, though there are awareness gaps among the community, the community is now understanding the benefit of the system. The main reason for the community to understand the benefits is learning from others who benefited from the system. You know they will see people who are poor and do not have the money to get the service, but they get a free service due to the insurance and are cured of their illnesses. This helps everyone in the community to understand the benefits.

**Interviewer**: **What are the factors that you think can facilitate household enrollment and continued membership in community-based health insurance?**

**KI:** One of the opportunities is the government’s attention and support. The political leadership has shown a strong commitment and focus to this system and it is appreciable. These days it is a political agenda and since politics is the center everyone at each administrative level is contributing to the system. You know health insurance is a means to support each other, as many pay and a few needy use the service which they could not have gotten if there was this system. This goes with the Ethiopian culture of supporting each other.

As I have mentioned earlier noticing exemplary individuals from neighbors who benefit from the CBHI is another reason and an opportunity for others in the community to enroll into the system.

The other opportunity is an increase in the number of health facilities in the community. You know these days there are health centers and hospitals nearby the community, therefore the community will not have access issues to enroll in the CBHI. They can enroll in the CBHI and get the service at their nearby facilities which are easily accessible.

The other opportunity is the presence of a health insurance policy and guidelines in the country. It also has an organizational structure from the federal level to the bottom which facilitates to easily accomplish tasks across the levels and engage the community in the system.

**Interviewer**: **What are the barriers to enrollment in community-based health insurance?**

**KI**: You know the health service to be provided to the CBHI users includes laboratory and pharmacy services. But recently drug supply has become a challenge to the health care system and is becoming a challenge to the CBHI too because the users do not get the prescribed medications from pharmacies in the public institutions buy them from private pharmacies frequently. Of course, there is a general shortage in drugs nationally because we import more than ninety percent of the drugs, need the support of many stakeholders including the shipment, facilities do not properly quantify the drugs they know, and there is a foreign currency shortage for purchasing.

As EPSA is not a profit company and the money in EPSA works in a revolving drug fund we get challenged to buy drugs due to a delay in the health facilities to pay their debts. Therefore, we started not to loan drugs to health facilities, which in turn resulted shortage of drugs at health facilities and a financial burden on the CBHI users.

This results from a deter in the membership to the system and suffering among the community. This also results in access problems as laboratory investigations or drugs the clients need may be expensive and sometimes unavailable in private facilities in some woredas with a final negative outcome of morbidity and mortality. This when summed is significantly affecting membership to the CBHI.

The false reports in the number of people enrolled, data quality issues, and false promises especially among the lower political leadership due to fear of accountability which I have raised earlier is a problem for the management of the CBHI system which in turn results in poor financial management and planning, dissatisfaction in the service, and drop out from the system.

Poor quality of service which is not as per the standard is the other barrier for the community to enroll in the system. The supplies including laboratory reagents and drugs shall be available for the health service users unless it will continue to be a barrier for the community member to enroll in the system and rust public health facilities.

**Interviewer**: **What strategies could be implemented to increase enrollment in community-based health insurance?**

**KI**: One of the strategies we could apply which has been started in some areas is engaging institutions, companies, and people with a better income in disposing of their social responsibility by contributing to the CBHI premium. For example, our institution has covered the CBHI payment of 100 community members for this year. I have also paid the CBHI premium for 5 community members who could not afford to pay it. So through this way we could the community members to enroll to the CBHI and also help the system financially.

Moreover, the financial management system should be improved. Starting from the premium collection it should be digitalized, needs to have a similar framework of collection in every corner of the country, and needs strong management support.

More than this what I think needs to be emphasized is improving the service quality at public health facilities. If a facility is opened as a health center, then it should provide services expected from its level. If there should be a laboratory service then there should be a laboratory service. If medications have to be there then we need to avail those drugs. Right now, there is good access to health facilities in terms of number but we need to reform the quality of service and provide the supplies needed to the facilities.

**Interviewer: Can you describe the organizational structure of the CBHI in your community and its effect on function?**

**KI**: Ok, when we see our health system there are three pillars: the one who provides service mainly the health facilities, the second is the one who receives service including the health insurance service and the third is the controlling body mainly the food and drug authority (FDA).

The problem in our structure is the health service received (The health insurance service) which is the managing structure of health insurance users under the health service providing body. This is a mix in the structure. There is even a gap in structural communication between the regional and federal and regional health insurance structures. For example, here in Hawassa, there is the federal CBHI structures branch office and then there is the Sidama region CBHI regional body. The Sidama regional CBHI is under the medical services unity of the regional health bureau which is led by the deputy health bureau official of the region. You see this medical services unit is the health service provider for the general community including the CBHI users but also it is this unit that runs and controls every activity of the CBHI in the region. This makes the data quality issues I have raised earlier false reporting very prone, and accountability very ambiguous.

Concerning the above point the political leadership needs to have key performance indicators to check and balance to check discrepancies in data between the federal and regional structures and verify data from woreda and other administrative levels. We need to see what is on the ground, we need strong monitoring and evaluation meanness.

For example, X Referral Hospital has a 14-million-birr debt from EPSA. When we requested the hospital to pay the debt, they responded that they couldn’t get the money for the money service they provide service to CBHI users from the health bureau. If the bureau had enrolled all this much community who pay to the CBHI why would it be a problem to pay for the service the users utilized, this means some users are members of the system without a contribution, this needs monitoring. However, the monitoring and evaluation are complicated because the provider and the one who receives the service are embedded under the same structure.

This problem may get complicated when we include civil servants in the CBHI. As the civil servant is a community with better awareness, it will ask if we can’t provide the service we promised. Therefore the structure needs to be reformed before we add another part of the community to the service.

**Interviewer: What are your thoughts on the leadership of the CBHI?**

**KI:** The leadership’s commitment is appreciable. But the leadership shouldn’t only focus on enrolling the community rather they should also monitor the quality of service provided at the facilities and try solving the bottlenecks that cause dissatisfaction among the CBHI users. Otherwise, prolonged problems can backfire on the service provider and the government. **Interviewer: Is there anything would you like to add?**

**KI:** We have raised a lot of points. The initiation is good and appreciable. But when we enroll the civil servants can create more challenges. Therefore, all stakeholders and political leaders shall work on its overall improvement beyond enrolling the community in the CBHI system.

**KK20: Key informant interview 20**

I: For transcription purposes, I’ll be recording our conversation, is that OK?

R: Yes, it is OK. No problem.

I: Thank you for joining us today. We're eager to delve into the intricate workings of Community-Based Health Insurance (CBHI) with your expertise. Firstly, could you shed some light on CBHI enrollment trends and the challenges associated with dropouts from the program? Moving on to the financial aspect, could you elaborate on premium contribution mechanisms and how they're managed within the CBHI framework? We've heard discussions regarding moral hazard, wherein insured individuals may over consume healthcare due to covered costs; could you outline its impact on CBHI operations? Additionally, how does adverse selection, where high-risk individuals are more inclined to enroll, affect the CBHI pool and its sustainability? Lastly, could you provide insights into specific considerations related to women within the CBHI context? Your expertise in navigating these nuanced aspects would greatly enrich our understanding.

R: CBHI was very good because one can use it only once when he feels pain. There are many benefits for those who are treated outside of the pelvis, for example, they get treatment without spending anything out of their pocket without the cost of bed and medicine.

It is because the health center in our district does not provide us with proper services, but it reduces the motivation of the people, but CBHI has a lot of importance.

I am not a CBHI user and I want to have one, but what made me not take it out here was when I saw that when people went with CBHI books and they did not give them good service, I gave up taking it out here. When he goes to be treated here, he is told to take medicine without any blood or other tests. So, if things are fixed, everyone will be a CBHI user.

I: How many people benefit from your Kebele?

R: In 2014, some people entered, but in 2015, more than half of the people entered, and when people go to get services, there is no medicine, it is called outside, the road is not comfortable, it is a forest, there is not even a place to eat. This person says that we will get medical treatment with the money we have saved, why is this happening? Therefore, people's motivation is reduced by lack of service and reception from health professionals.

I: Besides what you just said, what is it that keeps people from entering?

R: Nothing else. If a person who joined CBHI uses it, he will encourage others to do the same, but if a person comes in and does not get service and is treated like the other, he says, how can a person pay for it?

I: Does the community know the benefits?

R: Yes, as soon as he came, he took out the money that many people had saved for the time of trouble, and then they saw the problem in the service and reduced it, but he had a great initiative. A person I know went to our city and was treated and because he is a CBHI user, he was treated at a reasonable cost, but for better treatment, he was ordered to be treated at Hawassa Yanet Hospital, where a bed for one day is 3000 birr, but in our, he was treated for 11 nights with free medicine and treatment, but it was private. He had sold the property.

Since we are on the border, it would be good if we could use the nearby health center because of its proximity and service.

I: What about mothers or women aged 15-45?

R: It has a great advantage; our sisters or mothers see what they need during pregnancy and give advice and services related to her health if she is bleeding. Now not with us, pregnant women in Y district are given great care from pregnancy to delivery.

I: Does anyone walk without feeling any pain?

R: It's not like I told you before, even a sick person who is not sick is very difficult to walk because the road is far and it is not convenient even by motorbike.

I: What kind of person is a CBHI user in this area? Is he a sick person or is he rich and poor?

R: I use everyone. For example, someone thinks that he might get sick tomorrow, and everyone is coming in to fulfill a mission sent by the government.

I: Do you know who is running this CBHI service?

R: I don't know, but I think the district will bring it and send it to the HEWs, then from the HEWs to the Kebele chairman and his cabinets, and they will tell us and gather the people and tell them that something like this has happened and that they should register and use the service.

I: Which of the bodies you mentioned now has the largest share?

R: They are health extension workers (HEWs)

I: So do you think that the people you have just appointed will help CBHI to function well?

R: Yes, for example, a person who manages a nation can inspire. They deliver the money they collected from the people on time. They are doing a lot of work. The problem is with the health professionals. But sometimes it takes a long time before the money comes in and the year ends without us using it.

I: Where do you keep the money? Is it the district level or the kebele level?

R: It is in the district. They work with receipts every day. So there is no problem with my keel.

I: So, what do you think should be done to improve the problems you have raised so far and to strengthen CBHI from where it is now so that the community can get good services?

R: From 200 birr, 600 birr has been received, so if the money is adjusted, secondly, if the health professionals give the necessary treatment and medicine if they treat them properly, and thirdly, it is the distance from the road, because they are the ones who prevent the public from using it.

I: If there is anything else you would like to add or if there is an idea that should not be mentioned?

R: Nothing, that's it

I: Okay, thank you

R: Thank you too
