# Supplementary material for: Barriers and enablers of community-based health insurance enrollment in the Sidama national regional state, Southern Ethiopia, 2024: A qualitative study
Source: PLOS Glob Public Health. 2025 Sep 11;5(9):e0004310. doi: 10.1371/journal.pgph.0004310 (PMC12425181; doi:10.1371/journal.pgph.0004310)
Supplement: S4 Checklist — (DOCX) [file pgph.0004310.s004.docx]

Inclusivity in global research

PLOS’s policy on inclusivity in global research aims to improve transparency in the reporting of research performed outside of researchers’ own country or community and ensures that PLOS publications reporting global research adhere to high standards for research ethics and authorship. Authors of relevant research articles may be asked to complete the questionnaire below, which outlines ethical, cultural, and scientific considerations specific to inclusivity in global research. This questionnaire may be requested when researchers have travelled to a different country to conduct research, if research uses samples collected in another country, research with Indigenous populations or their lands, or if research is on cultural artefacts. Researchers travelling to another country solely to use laboratory equipment will not normally be required to complete the questionnaire. However, the questionnaire can be requested at the journal’s discretion for any submission – if you have been requested to complete this questionnaire by the PLOS journal you submitted to, please do so.

Please complete the questionnaire below and include this as a Supporting Information file with your manuscript. Note that if your paper is accepted for publication, this checklist will be published with your article in the supporting information files. Please ensure that you reference the checklist in the main body of your manuscript. We suggest adding a subsection ‘Inclusivity in global research’ to your Methods section and adding the following sentence: “Additional information regarding the ethical, cultural, and scientific considerations specific to inclusivity in global research is included in the Supporting Information (SX Checklist)”

The questions have been designed to be applicable to a wide range of study types, and there are subsections for both human subjects research and non-human subjects research. If any of the questions are not relevant to your research please mark them as “N/A” as appropriate.

**Ethical considerations, permits and authorship**

*This section is applicable to all research types.*

Provide details as to who granted permissions and/or consent for the study to take place in the Methods section of your manuscript. This should include the names of **all** ethics boards, governmental organizations, community leaders or other bodies that provided approval for the study. If individuals provided approval refer to these people by their role or title but do not list their name(s).

Reported on page number: 15-16

If there were any deviations from the study protocol after approval was obtained please provide details of these changes in the Methods section of your manuscript.
Did this study involve local collaborators that are residents of the country where the research was conducted or members of the community studied? If you do not have any authors from said communities, please provide an explanation for this below.

Reported on page number: N/A. It was conducted as per the approved protocol without any deviations.

Local stakeholders, including the regional health bureau, health offices from woreda/city administrations, health extension workers, kebele leaders, and community leaders, played a vital role in the following ways; briefing about the local context. Some were supported in identifying study study area & sample, and others were supported as local guides. However, these entities were not involved in any part of the study's design, data collection, curation, analysis, interpretation, or report writing.

Everyone listed as an author should meet PLOS’ criteria for authorship and all individuals who meet these criteria should be included in the author byline, rather than the acknowledgments. For further information please see the journal’s Authorship Policy.

**Human subjects research (e.g. health research, medical research, cross-cultural psychology)**

Did you obtain written informed consent from a representative of the local community or region before the research took place? How did you establish who speaks for the community? Details of written informed consent obtained from study participants should be reported separately in the Methods section of your manuscript.

Written informed consent was obtained from each study participant before data collection activities, as the letter was granted by the Institutional Review Board (IRB) of the College of Medicine and Health Sciences at Hawassa University.

To validate the representativeness of local community consent, we conducted preliminary engagements with community leaders and stakeholders, including representatives from local health organizations and government institutions. These interactions enabled us to identify key informants who have recognized roles in decision-making processes and facilitated the collection.

3. **Did this study involve local collaborators? If not, provide an explanation.**

- **Answer:** Yes

**Explanation:** Local collaborators participated in data collection, analysis, and interpretation to ensure cultural relevance and appropriateness.

4. **Did all authors meet PLOS’ criteria for authorship?**

- **Answer:** Yes

**Explanation:** All listed authors contributed significantly to the study and met PLOS' authorship criteria.

---

## Human Subjects Research

1. **Was written informed consent obtained from a representative of the local community? How was this person identified?**

- **Answer:** Yes

**Explanation:** Written informed consent was obtained from representatives identified through consultations with local leaders and stakeholders.

2. **How did members of the local community provide input on research aims, methodology, and outcomes?**

- **Answer:** Yes

**Explanation:** Community members were consulted during the planning phase to align research aims with their priorities and provided feedback on methodology and anticipated outcomes.

3. **How were informed consent documents made understandable for local stakeholders?**

- **Answer:** Yes

**Explanation:** Documents were translated into [local language(s)] and reviewed by local collaborators to ensure clarity. Verbal explanations and visual aids were also provided.

4. **How will findings be shared with stakeholders in an understandable format?**

- **Answer:** Yes

**Explanation:** Findings will be shared through community presentations, summary reports in [local language], and accessible publications.

---

## Non-Human Subjects Research

1. **Did permission include agreements on access to outputs and benefit sharing?**

- **Answer:** Yes

**Explanation:** Permission agreements included provisions for fair benefit sharing and access to research outputs as per the Nagoya Protocol.

2. **If materials were imported, provide details on permits:**

- **Answer:** Yes

**Explanation:** Materials were imported in 2023 under Permit #12345 issued by [relevant authority]. All necessary import/export permits were secured.

3. **If archival specimens were used, how were they acquired?**

- **Answer:** Yes

**Explanation:** Archival specimens were housed at [Name of Institution] and originally acquired under Permit #67890 issued by [relevant authority].

4. **How was cultural significance considered in research design? Were Indigenous peoples/local researchers involved?**

- **Answer:** Yes

**Explanation:** Cultural significance was addressed through consultations with Indigenous representatives and local researchers who actively participated in the study design and fieldwork.

5. **Were permissions obtained for photographs of human remains (if applicable)?**

- **Answer:** No (if applicable) / N/A (if not applicable)

**Explanation:** This study did not involve photographs of human remains OR permissions were not required as no such materials were used.

Written informed consent was obtained from each study participant before data collection activities, as granted by the Institutional Review Board (IRB) of the College of Medicine and Health Sciences at Hawassa University.

To validate the representativeness of local community consent, we conducted preliminary engagements with community leaders and stakeholders, including representatives from local health organizations and government institutions. These interactions enabled us to identify key informants who have recognized roles in decision-making processes and facilitated the collection.

How did members of the local community provide input on the aims of the research investigation, its methodology, and its anticipated outcome(s)?

Our study team consulted with local leaders and government representatives. They provided valuable input on the study aims, methodology, and anticipated outcomes.

When engaging with the local community, how did you ensure that the informed consent documents and other materials could be understood by local stakeholders?

The study guide, protocols, & consent form were translated into the local language (*Sidamu Afoo)* and reviewed by bilingual experts to ensure clarity.

Will the findings of the research be made available in an understandable format to stakeholders in the community where the study was conducted (e.g. via a presentation, summary report, copies of publications, etc.)? Please provide details of how this will be achieved.

Findings will be shared through community presentations, summary reports in the local language (*Sidamu Afoo*), and accessible publications

**Non-human subjects research using specimens/ animals collected as part of the study, or those housed in archival collections. Examples include archaeology, paleontology, botany and zoology.**

Did the permission you obtained from a local authority to perform the study include an agreement on access to outputs and benefit sharing? This may include procedures to enable fair distribution of the benefits and resources arising from the research performed. Please include any details of Prior Informed Consent and Benefit Sharing Agreements obtained. These may be required by field-specific regulations, for example the Convention on Biological Diversity (CBD) and the associated Nagoya Protocol.

N/A,

If the material used in your study was imported, please A) provide the year it was imported and B) indicate whether permits were obtained to import/export the materials used, C) provide details of any permits obtained. If this information is not available, please indicate this.

N/A

If you used archival specimens, please state how the material used in your study was acquired by the institute it is held in and provide details of any permits obtained for the original excavations/ sample collection. If this information is not available, please indicate this.

N/A

How was the potential cultural significance of the materials collected in your study to local communities considered in your research design? Were Indigenous peoples and/or local researchers and institutions involved with archaeological excavations/collection of specimens? If so, please provide a description of their involvement.

N/A

If your manuscript includes photographs of human remains please indicate whether authors obtained permission from descendants or affiliated cultural communities to do so.

N/A
